# Access to Genetic Testing in Patients With Suspected Genetic Kidney Disease in Europe

**DOI:** 10.1016/j.ekir.2026.107070

**Published:** 2026-09-08

**Authors:** Margriet E. Gosselink, Femke Buining, Jens König, A. Titia Lely, Henk Groen, Laura R. Claus, Kerry Leeson, Laurence Heidet, Sophie Saunier, Franz Schaefer, Max C. Liebau, Tess Harris, Albertien M. van Eerde

**Affiliations:** 1 Department of Genetics, University Medical Centre Utrecht, Utrecht, The Netherlands; 2 Department of Obstetrics and Gynecology, Wilhelmina Children’s Hospital Birth Centre, University Medical Centre Utrecht, Utrecht, The Netherlands; 3 Department of Epidemiology, University Medical Centre Groningen, Groningen, The Netherlands; 4 Department of General Pediatrics, University Children’s Hospital, Münster, Germany; 5 Alström Syndrome UK (ASUK), Torquay, Devon, UK; 6 Department of Pediatric Nephrology, Hereditary Kidney Diseases Laboratory, Reference Center for Inherited Renal Diseases (MARHEA), Necker-Enfants Malades University Hospital, Assistance Publique Hôpitaux de Paris (AP-HP), Inserm UMR 1163, Imagine Institute, Paris Cité University, Paris, France; 7 Laboratory of Hereditary Kidney Diseases, Institut Imagine, Paris, France; 8 Division of Pediatric Nephrology, Heidelberg University Center for Pediatrics and Adolescent Medicine, Heidelberg, Germany; 9 Department of Pediatrics, University Hospital RWTH Aachen, Aachen, Germany; 10 PKD Charity UK, London Office, PKD International, London, UK

**Keywords:** Chronic kidney disease, barriers, genetics

## Abstract

**Introduction:**

A genetic diagnosis can transform care for patients with monogenic kidney disease—guiding treatment, prognosis, family-planning, and care for relatives. Despite technological advancements, nephrologists still experience barriers to implementing diagnostic genetic testing in daily practice.

**Methods:**

We surveyed 240 pediatric nephrologists, adult nephrologists, and clinical geneticists across 39 European countries on the accessibility of and barriers to genetic testing for suspected genetic kidney disease. Using the Consolidated Framework for Implementation Research (CFIR) model, we assessed barriers at national, hospital, individual, and implementation-process levels. Results were stratified by region (North, East, South, West Europe, and non-Organization for Economic Cooperation and Development (OECD) countries). Binary logistic regression identified factors associated with barrier experience.

**Results:**

Overall, 97% regarded genetic testing an important tool to improve care, yet 50% experienced barriers in daily practice. Median barrier severity score was 4.0 (interquartile range [IQR] 4, scale 1–10). Non-OECD countries showed higher scores (6.8 (IQR 3.6), *P* < 0.001). Most reported national-level barriers were lacking clear professional guidelines and concerns about posttest insurability. Hospital-level barriers included insufficient clinic’s resources (costs, time, technical facilities, and staffing), missing protocols for eligible patients or workflow and turnaround time (nonurgent: 12 weeks [IQR 12], urgent: 3 weeks [IQR 2]). Ranking barriers by region revealed a gradient from fewest barriers in Northern Europe, followed by West, South, East, and most in non-OECD countries. Lack of external funding and non-OECD origin were associated with barriers.

**Conclusion:**

We identified barriers that must be addressed at a European level and within regions. We provide recommendations to promote equitable care for patients with suspected hereditary kidney disease in Europe.

## Graphical abstract

**Figure:**
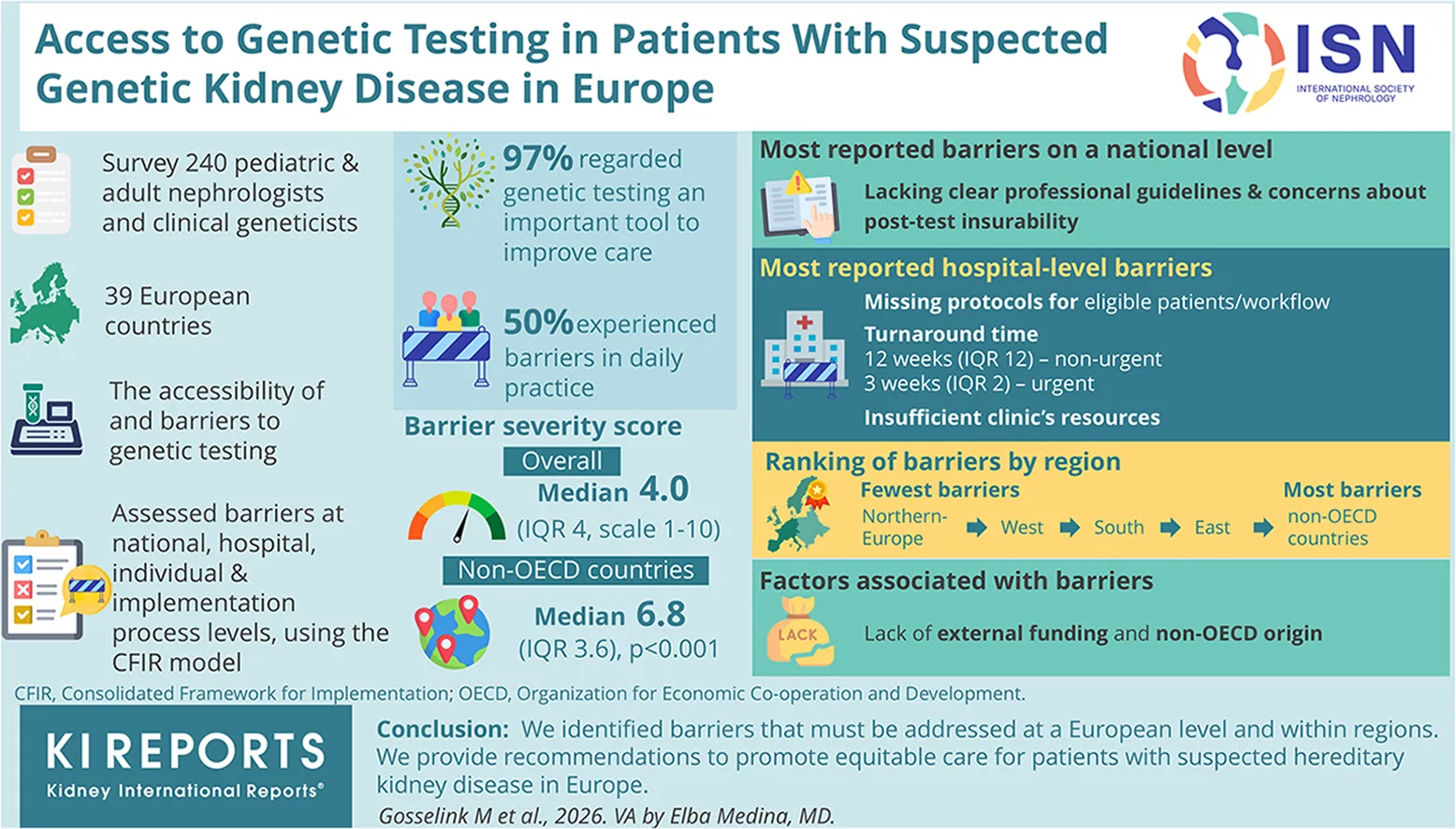

Monogenic diseases are a significant cause of chronic kidney disease (CKD), contributing to approximately 70% of kidney failure cases in children and 10%–15% in adults.^1^, ^2^, ^3^, ^4^, ^5^, ^6^, ^7^ Despite the lower proportion in adults, their higher CKD prevalence results in a greater absolute patient burden.

Genetic testing has become an invaluable tool in the diagnosis of kidney diseases, often offering insights for management and treatment that traditional diagnostic methods cannot provide.^8^ Advances in genetic sequencing and data analysis have greatly improved the diagnostic yield of genetic testing for kidney disease, with over 600 associated genes now identified.^9^ Detecting disease-causing genetic variants in patients with CKD can inform prognosis and enable personalized care, including kidney protection strategies, targeted therapies, and transplant decisions, and is essential for genetic counseling and family planning.^8^

Although monogenic diseases are prevalent as a group, each individual condition is rare.^7^^,^^10^^,^^11^ In 2017, the European Union (EU) established European Reference Networks (ERNs), focused on care for rare or low-prevalence complex diseases. The declared goal of ERNs is to facilitate the exchange of scientific knowledge and specialized expertise within and across networks internationally, aiming to improve access to highly specialized health care at local, regional, and national levels.^12^ For rare kidney diseases, the European Rare Kidney Disease Reference Network (ERKNet) was established.^2^

Despite recent technological advances described above, nephrologists still experience barriers to implementing routine use of diagnostic genetic testing in clinical practice.^13^^,^^14^ In Europe, data indicate that the number of laboratories offering clinical tests does not correspond to the population size they serve. In addition, the range of tests available varies significantly between countries, influenced by a West-East geographic gradient.^15^ Furthermore, previous research showed significant disparities in staffing levels per capita in clinical genetics units across Europe. Because EU funding for rare disease research is not evenly distributed, in Europe inequities in the care of patients with rare disease exist.^16^ However, a European-focused study investigating barriers to the implementation of genetic testing for patients with suspected genetic kidney disease is still lacking.

To assess inequities in genetic kidney diseases, we conducted a survey study involving pediatric and adult nephrologists as well as clinical geneticists to investigate the accessibility of genetic testing for patients with suspected genetic kidney disease across Europe.

## Methods

### Survey Design

An anonymous online survey study was conducted, targeting pediatric and adult nephrologists as well as clinical geneticists across Europe (Supplementary Material S1). For reporting of this survey, we used the Checklist for Reporting Results of Internet E-Surveys and the Checklist for Reporting of Survey Studies.^17^^,^^18^ The University Medical Centre Utrecht medical ethics committee confirmed that ethical approval was not required (reference 23U-0073). Informed consent was asked at the first page of the survey, that included information on the purpose of the survey, the investigators, the length of the survey, usage of data, and withdrawal. All personal data was pseudonymized, and all data were kept in a locked file on secured UMC Utrecht servers.

Supplementary Figure S1 illustrates all steps of the survey design. The questions were designed by a review of relevant literature and established tools, including the CFIR, a widely used theoretical model in genomic medicine that supports the development, assessment, and implementation of health care interventions.^19^ With the CFIR model, survey questions were designed assessing barriers in a systematic manner on different levels—participant’s outer setting (country of residence), inner setting (hospital participant is working in), individual setting (own assumptions/beliefs), and on the process (implementation of genetic services). Also, survey questions were adapted from the “Implementing Genomics in Practice” provider questionnaire.^20^^,^^21^

### Survey Content

The survey consisted of the following 3 parts: part i) demographic questions, part ii) questions addressing logistics of genetic testing in the participant’s clinic, and part iii) identifying barriers on the 4 CFIR domains—outer, inner, individual, and process setting, by reviewing statements with a 5-point Likert-scale ranging from strongly disagree to strongly agree, including options “I don’t know” and “not applicable.”

Survey questions were developed and iteratively refined by our international research group, which included clinicians from pediatric nephrology and clinical genetics, researchers, and a patient representative. The survey was improved by conducting a pilot study among a sample of 17 international clinicians (involving pediatric and adult nephrologists as well as clinical geneticists) in the research group’s network. Based on their feedback and suggestions for improvement, the survey was finalized. Before fielding the final questionnaire, usability and technical functionality of the electronic questionnaire was tested by members of the research team.

### Survey Administration

An open-survey link was created through the online data capture tool Castor (version 2024).^22^ The survey was designed with adaptive questioning. The questionnaire was distributed across 9 pages, with a range of 3–32 questions per page. Completeness checks before submitting the questionnaire were done by Castor, including the use of mandatory questions. Respondents were able to review and change their answers before submitting the questionnaire. Multiple entries from the same individual were prevented by asking for an e-mail address; either participants’ own or a generated anonymous e-mail pseudonym. Double entries were filtered out in data analysis.

### Recruitment Process & Survey Sample

The open-survey link was distributed through the mailings lists of professional clinicians’ associations—European Renal Association (targeting mainly adult nephrologists, reaching *n* = 37500), ERKNet (targeting adult and pediatric nephrologists and geneticists, reaching *n* = 825), European Society for Pediatric Nephrology (targeting pediatric nephrologists, reaching *n* = 1600), and ERN Ithaca (targeting geneticists, reaching *n* = 71) between May 30, 2024 and October 30 2024. A total of 2 reminders were sent out. Furthermore, the open-survey link was shared with the researcher group’s professional network through social media. A map highlighting countries without survey responses was created and shared through reminders and social media to encourage participation from professionals across all European countries in the survey.

### Comparison With the European Rare Kidney Disease Registry Database

The European Rare Kidney Disease Registry (ERKReg) database collects data on patients with rare kidney diseases from 24 European countries, including clinical diagnosis and details on genetic diagnosis. For this study, we extracted ERKReg data on (i) number of patients with an indication for genetic testing per European country and region, (ii) number of truly genetically tested patients with a testing indication.^2^ Supplementary Table S1 shows a list of diagnoses and genetic testing indications.

These objective measures were compared against self-reported survey responses to assess alignment and discrepancies. To account for differences in country population sizes, we normalized numbers to population size using data from EuroStat.^23^

### Statistical Analysis

We calculated completion rates as the ratio of participants that finished the survey divided by the users who agreed to participate. Only responses including more information than general baseline characteristics were included for analysis.

Survey responses were summarized by descriptive statistics. Responses on barrier questions were reported for the total group, and in subgroups of different European regions. For subgrouping European regions, we used the most common welfare state typologies in public health and sociological research, which group OECD countries into North (Nordic), East (Central & Eastern Europe), South (Mediterranean), and West Europe (Continental & Anglo-Saxon countries).^24^ We added a fifth category for respondents originating from non-OECD countries.

Continuous variables were reported as means (SD) in case of a normal distribution. Variables with skewed distribution were reported as median with IQR.

A total of 2 survey questions were compared across European regions using χ^2^and Kruskal-Wallis tests—(i) “Do you experience barriers to genetic testing for patients with suspected hereditary kidney disease in daily practice? And (ii) if yes: “To what degree do you experience barriers (scale 1–10).” Univariable and multivariable logistic regression analyses were conducted to investigate associations with the experience of barriers. Furthermore, subanalyses were conducted for OECD regions, to identify associations specific for this subgroup. Dummy variables were created, comparing “1” versus all others (“0”) without direct category comparisons. Variables with *p* < 0.05 in univariable analyses were included in the multivariable logistic regression model. Results are presented as odds ratios (OR) with 95% confidence intervals.

For other survey questions, only trends were reported, and no statistical testing was performed. Analyses were performed using IBM SPSS Statistics version 29.0.1.0 (SPSS Inc.).

### Therapeutics for Renal Ciliopathies Consortium

This study was conducted within the Therapeutics for Renal Ciliopathiesconsortium. Renal ciliopathies comprise a large group of rare and ultrarare monogenic kidney diseases that as a group are responsible for > 10% of CKD in the pediatric population and form a significant cause of CKD for adults as well.^2^^,^^25^ Despite the huge relevance of this disease group, little causative treatment options exist so far. The Therapeutics for Renal Ciliopathies consortium was established with EU funding, to promote clinical trial readiness for renal ciliopathy patients. Amongst others, optimal accessibility to genetic testing for patients with CKD in Europe will facilitate identification of patients for future therapeutic interventions.^26^

## Results

### Response Rates

In total, 266 participants provided informed consent to the survey. A total of 26 of 266 (10%) only provided demographic information or originated outside of the European continent and were excluded from analyses. Therefore, 240 participants were included in the analyses. Completion rate of the included responses was 213 of 240 (89%).

### Baseline Characteristics

In Table 1 and Supplementary Figure S2, demographic characteristics of survey participants are shown. In total, 39 different European countries were represented in the survey (Figure 1; Supplementary Figure S3 and Supplementary Table S2 classify countries by region and list their participants). The survey sample consisted of 123 (52%) pediatric nephrologists, 85 (36%) adult nephrologists, 15 (6%) clinical geneticists, 6 (2%) professionals combining pediatric and adult nephrology, 4 (2%) combining clinical genetics and adult nephrology, and 6 (2%) having an “other” profession. A total of 207 of 240 (86%) worked in an academic setting, 137 (59%) worked in an ERKNet Reference Center or ERKNet Affiliated Center (Supplementary Figure S4A–B). Supplementary Table S3 and Supplementary Figure S5A–D show logistic details of genetic testing for survey participants stratified per European region. Most survey participants (63%) sent genetic tests to a laboratory in their own hospital. In non-OECD countries, 40% of participants sent samples to commercial laboratories abroad. Advanced nuclear DNA sequencing techniques (e.g., genome sequencing, exome sequencing [ES], and gene panel testing [enrichment and exome-based]) are available in most locations of diagnostic testing, ranging from 64% if samples were sent to hospitals abroad to 84% in participants’ own hospital.

**Table 1:** Baseline characteristics of survey participants

Variable	Frequency, total cohort N = 240
Survey completed	213/240 (89%)
Profession (including residents)	
Pediatric nephrologist	123/239 (52%)
Adult nephrologist	85/239 (36%)
Clinical geneticist	15/239 (6%)
Pediatric & adult nephrologist combined	6/239 (3%)
Clinical geneticist & adult nephrologist combined	4/239 (2%)
Other (i.e., lab specialist, genetic counselor)	6/239 (3%)
Involved in (multiple options)	
Adult care	118/239 (49%)
Pediatric care	138/239 (58%)
Prenatal care	58/239 (24%)
Participants per region	
North	14/240 (6%)
South	58/240 (24%)
West	92/240 (38%)
East	31/240 (13%)
Non-OECD	45/240 (19%)
Work setting	
Academic hospital	207/240 (86%)
General hospital	29/240 (12%)
Private clinic	13/240 (5%)
Other	4/240 (2%)
Working in clinic connected to ERKNet	
ERKNet Reference Center	105/231 (46%)
ERKNet Affiliated Center	32/231 (14%)
Center not connected to ERKNet	94/231 (41%)
Age, yrs	
20–29	6/240 (3%)
30–39	50/240 (21%)
40–49	80/240 (33%)
50–59	65/240 (27%)
60–69	37/240 (15%)
> 70	2/240 (1%)
Experience in profession, yrs	
0–5	12/240 (5%)
6–10	33/240 (14%)
11–15	48/240 (20%)
16–20	39/240 (16%)
> 20	108/240 (45%)
Days (outpatient) clinic care/wk	
1	30/240 (13%)
2	46/240 (19%)
3	39/240 (16%)
4	40/240 (17%)
5	80/240 (33%)
None	5/240 (2%)
Exposure to patient population suspect for hereditary kidney disease	
0%–10%	57/235 (24%)
10%–25%	83/235 (35%)
25%–50%	53/235 (23%)
50%–75%–90%75%	25/235 (11%)
	11/235 (5%)
90%–100%	6/235 (3%)
Frequency of requesting genetic testing or referring a patient for genetic testing	
> 1/wk	59/240 (25%)
> 1/mo	119/240 (50%)
> 1/yr	56/240 (23%)
< 1/yr	6/240 (3%)

**Figure 1:**
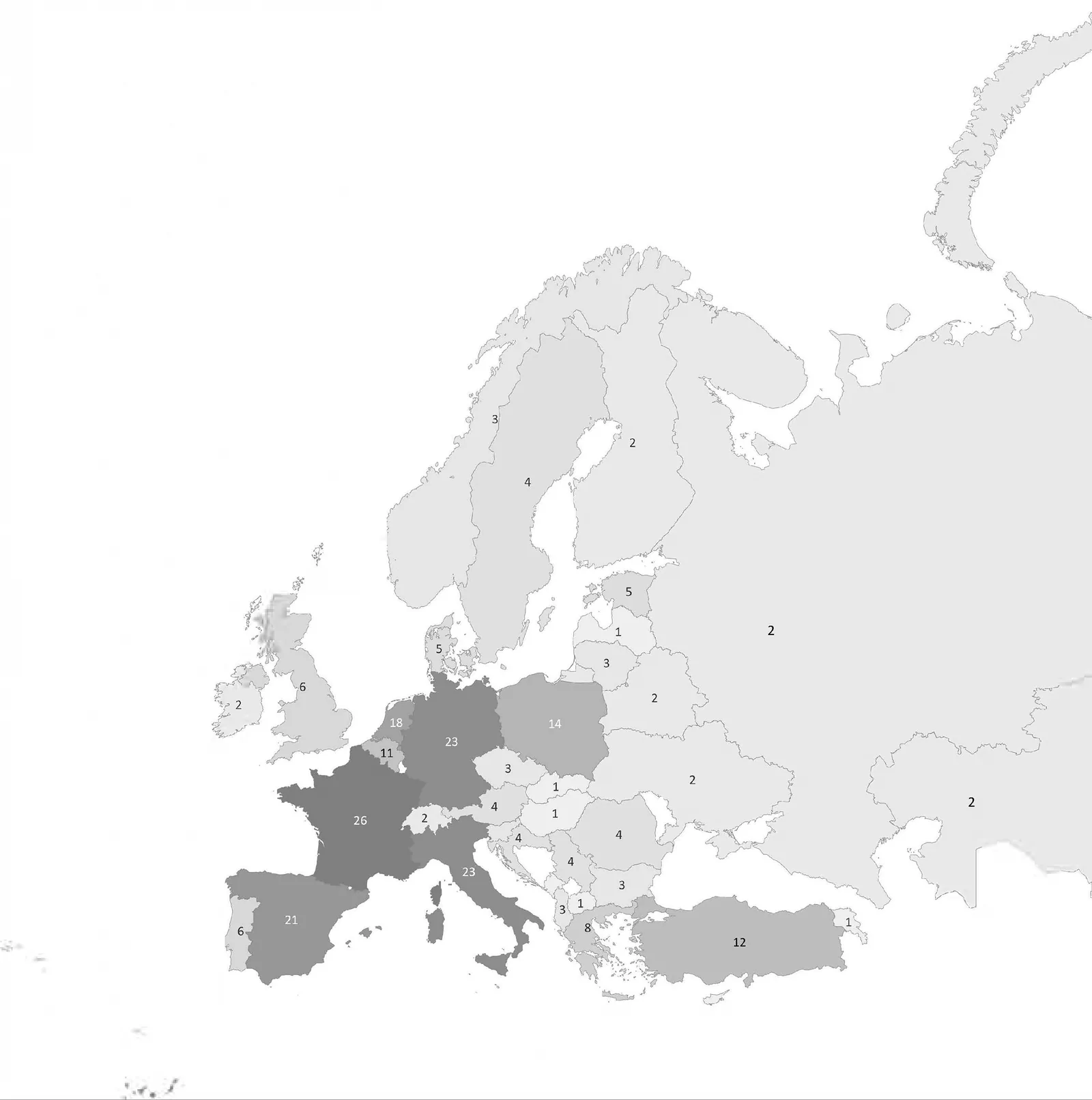
Survey respondents by country. The darker grey, the more survey responses.

### Barriers Overall

Overall, 113 of 228 (50%) of participants experienced barriers to the (further) implementation of genetic testing for patients with suspected genetic kidney disease in their daily practice. Per European region, this was 34/ of 44 (77%) for non-OECD countries, 17 of 27 (63%) for East, 23 of 56 (41%) for South, 34 of 87 (39%) for West, and 5 of 14 (36%) for North European countries (*P* < 0.001). Of 136, 60(44%) participants working in centers connected to ERKNet experienced barriers versus 53 of 92 (58%) working in nonconnected centers. Participants who encountered barriers were asked to rate the severity of these barriers on a scale ranging from 1 to 10. The median severity score was 4.0 (IQR 4, scale 1–10) and differed significantly between regions. It was highest in non-OECD countries (6.8 (IQR 3.6), corrected *P* < 0.001, Figure 2).

**Figure 2:**
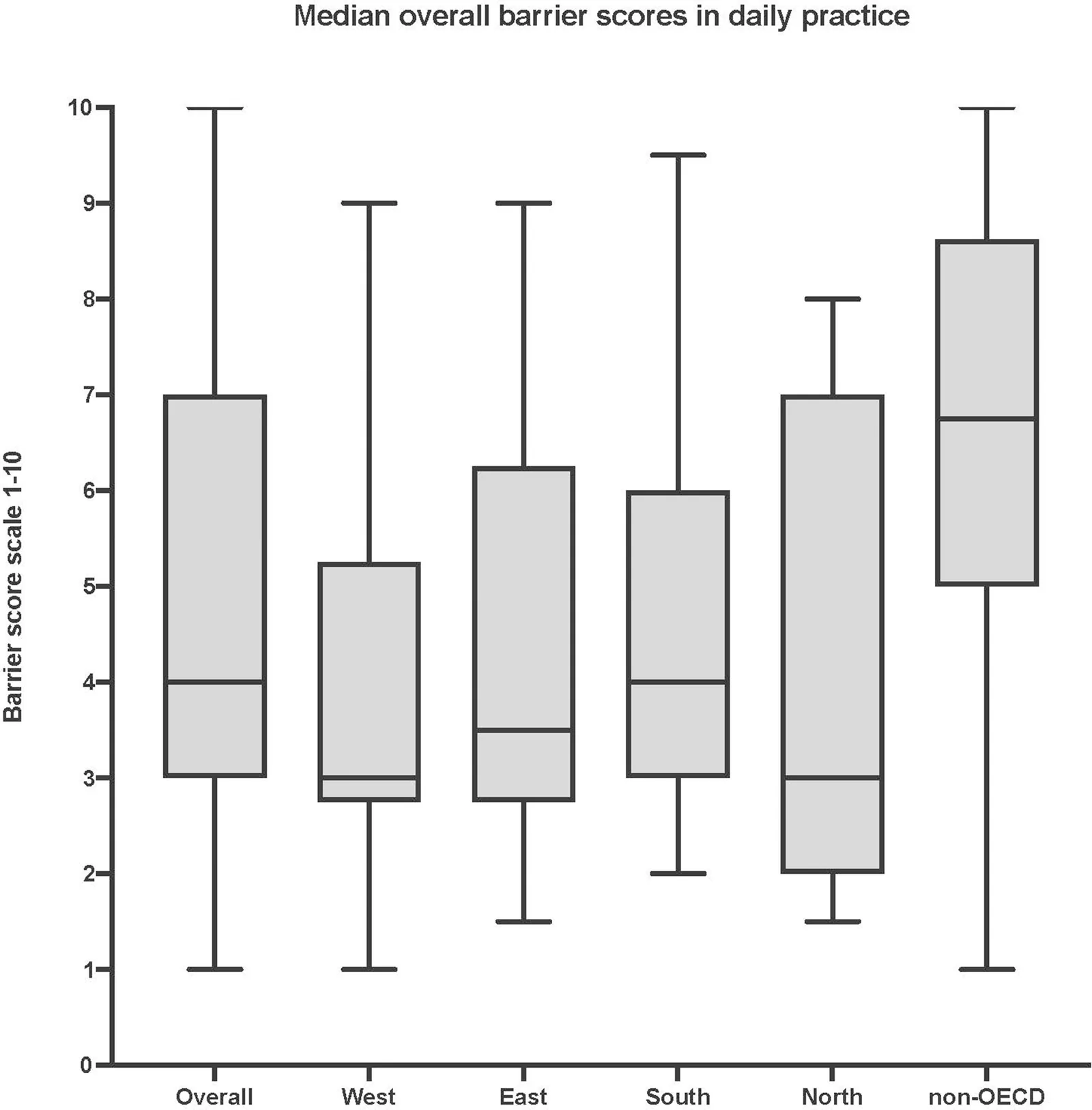
Median severity score for barriers experienced in daily practice. Overall scores and per European region (scale 1–10). Median overall score was 4.0 (IQR 4) for *n* = 113 participants, 3.0 (IQR 2.5) in West for *n* = 34 participants, 3.5 (IQR 3.5) in East for *n* = 17 participants, 4.0 (IQR 3) in South for n = 23 participants, 3.0 (IQR 5) in North for *n* = 5 participants, and 6.8 (IQR 3.6) in non-OECD countries for *n* = 34 participants (*P* < 0.001). OECD, Organization for Economic Cooperation and Development; IQR, interquartile range.

In the outer setting, 77 of 221 (35%) of participants missed clear professional group guidelines (In Figure 3 and Supplementary Table S4, the barriers per setting are displayed for all survey participants). Also, 61 of 221 (28%) were concerned about posttest insurability of patients. Most barriers were reported in the inner setting. Of 219, 84 (38%) missed a clear protocol and workflow for eligible patients. In total, 78 of 231 (34%) reported high costs and 65 of 219 (30%) considered the clinic’s financial resources insufficient. Also, 65 of 220 (30%) did not have enough time to facilitate genetic testing. In the individual setting, motivation for (further) implementation was high—211 of 217 (97%) responded that genetic testing improved ability to care for their patients and 206 of 216 (95%) believed genetic testing was an improvement over nongenetic clinical investigation. Also, participants were confident in their own abilities to use the results of genetic testing in daily practice and that their training had prepared them to care for patients at risk for genetic kidney disease. In the individual setting, turnaround time was the most reported barrier, reported as 12 weeks (IQR 12) for nonurgent and 3 weeks (IQR 2) for urgent requests. Regarding the domain of “process,” most participants were satisfied with the clarity about the designated person or team leading the (further) implementation efforts, as well as with their quality, skills, and the variety of strategies used (151/214 [71%], 134/214 [63%], and 161/214 [75%], respectively).

**Figure 3:**
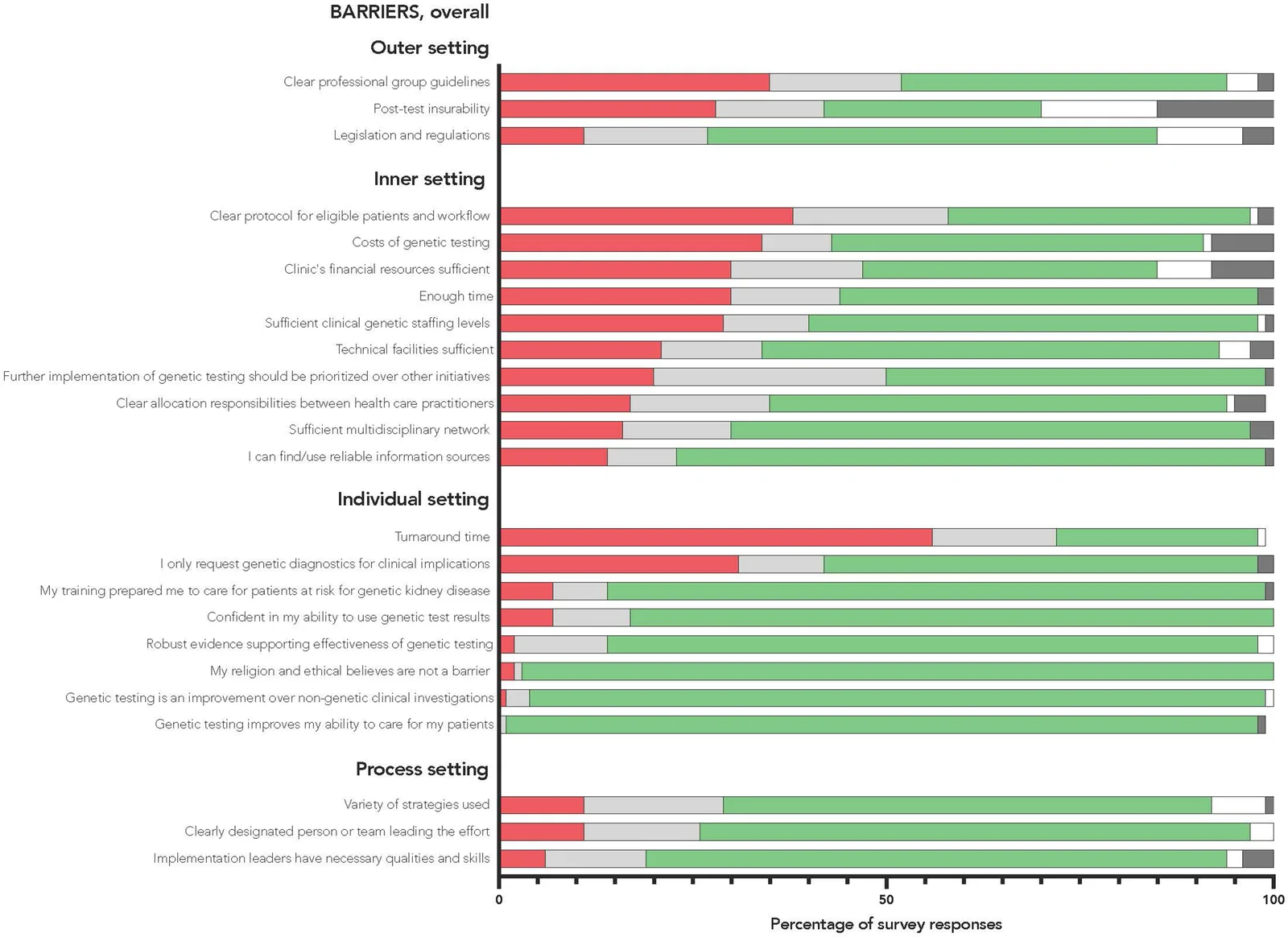
Implementation barriers for genetic testing across Europe. This figure shows overall responses to barrier statements regarding implementation of genetic testing for suspected hereditary kidney disease in 39 European countries (*n* = 214–231 survey responses). Responses are categorized as red (disagreed, indicating a barrier), light grey (neutral), green (agreed, indicating no barrier), white (I don’t know), and dark grey (NA).

### Barriers Per European Region

Ranking of most reported barriers by European region revealed a gradient, from least barrier experience in Nordic regions, followed by West, South, East, to greatest in non-OECD countries (Table 2 provides a summary of most reported barriers in different European regions).

**Table 2:** Most reported barriers stratified per European region

Barriers per setting	Overall	West	East	South	North	Non-OECD
Outer setting						
Clear professional group guidelines lacking	35%	24%	41%	37%	43%	45%
Concerns about posttest insurability	28%	36%	27%	19%	7%	29%
Inner setting						
Clear protocol for eligible patients/workflow is missing	38%	35%	27%	35%	50%	52%
Costs of genetic testing	34%	18%	21%	38%	14%	73%
Clinic has insufficient resources	30%	24%	27%	29%	14%	48%
Not enough time	30%	22%	42%	39%	29%	26%
Insufficient staffing levels	29%	21%	31%	39%	7%	38%
Technical facilities insufficient	21%	17%	12%	24%	0%	36%
Individual setting						
Turnaround time is too long	56%	57%	65%	63%	29%	50%
Specific for regions, not for overall						
A clear allocation of responsibilities between care practitioners lacking	17%	12%	23%	20%	7%	26%
Sufficient multidisciplinary network	16%	8%	15%	22%	14%	29%
Cannot find/use reliable sources of information	14%	8%	12%	21%	21%	17%

Grey = barrier reported ≥ 20%, white = barrier reported < 20%. The more frequently reported, the darker grey the barrier was.

In Figure 4a–c, Supplementary Table S5–S9, and Supplementary Figures S5–S8 barriers in different settings are grouped per European region.

**Figure 4:**
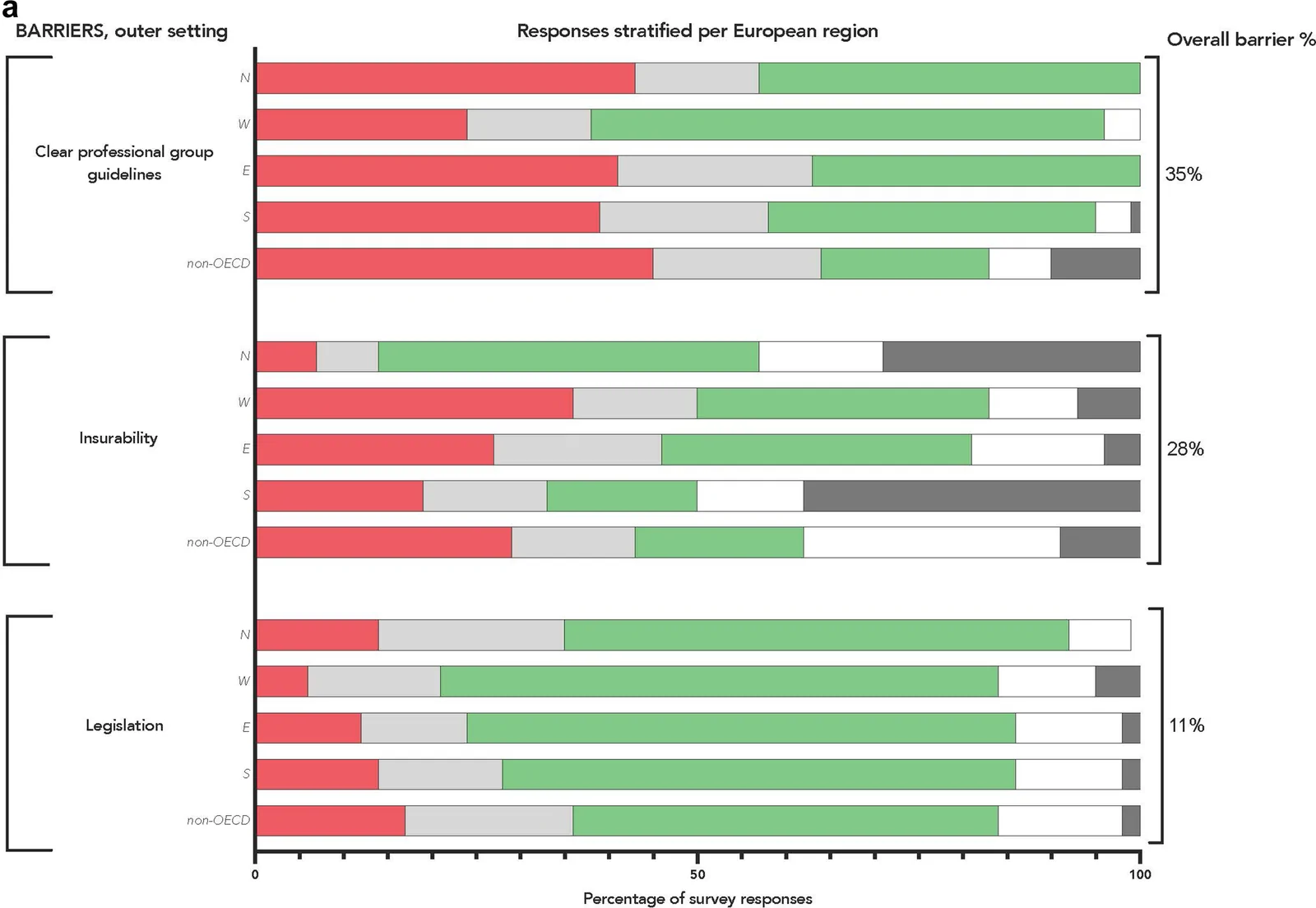

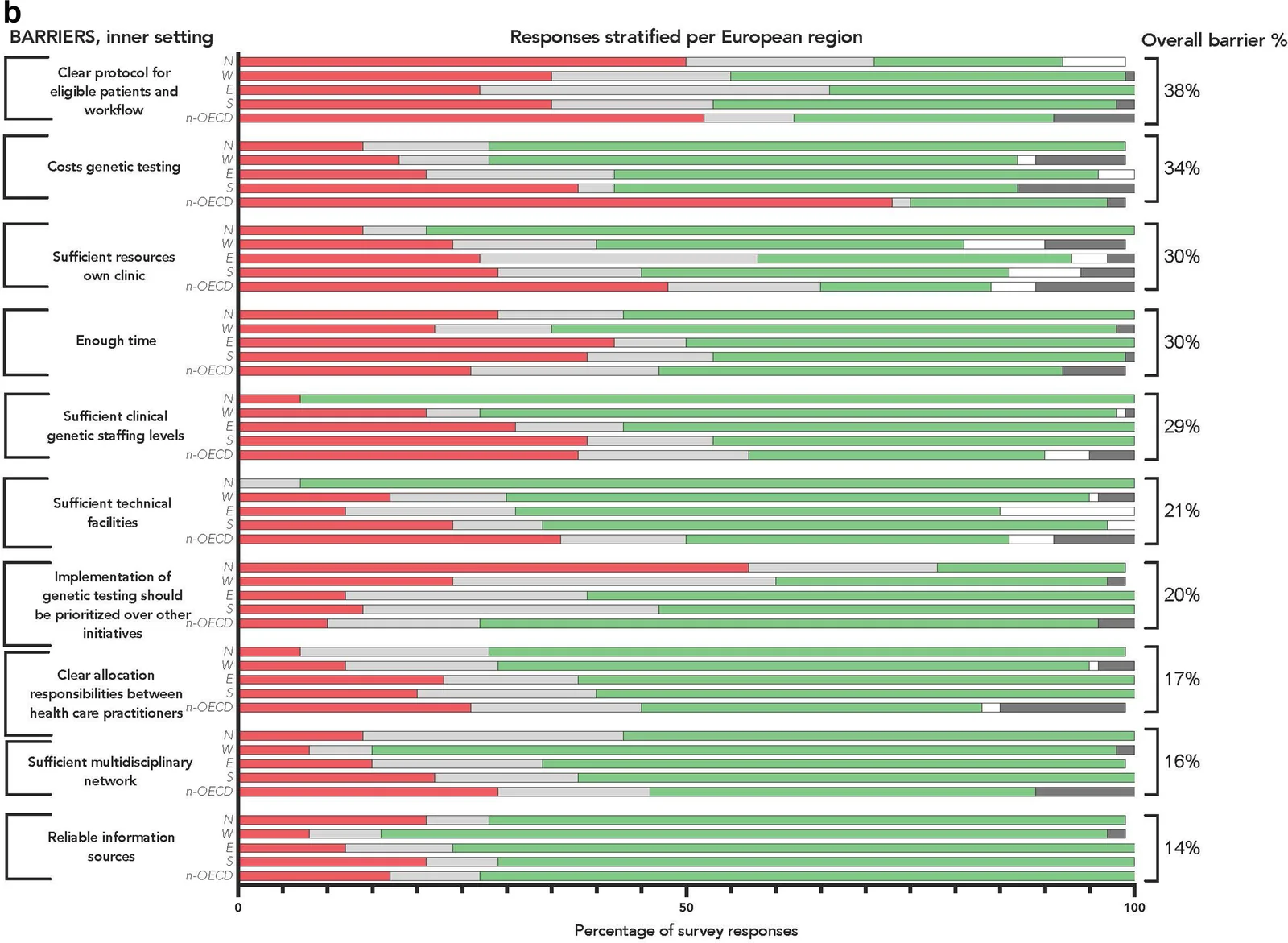

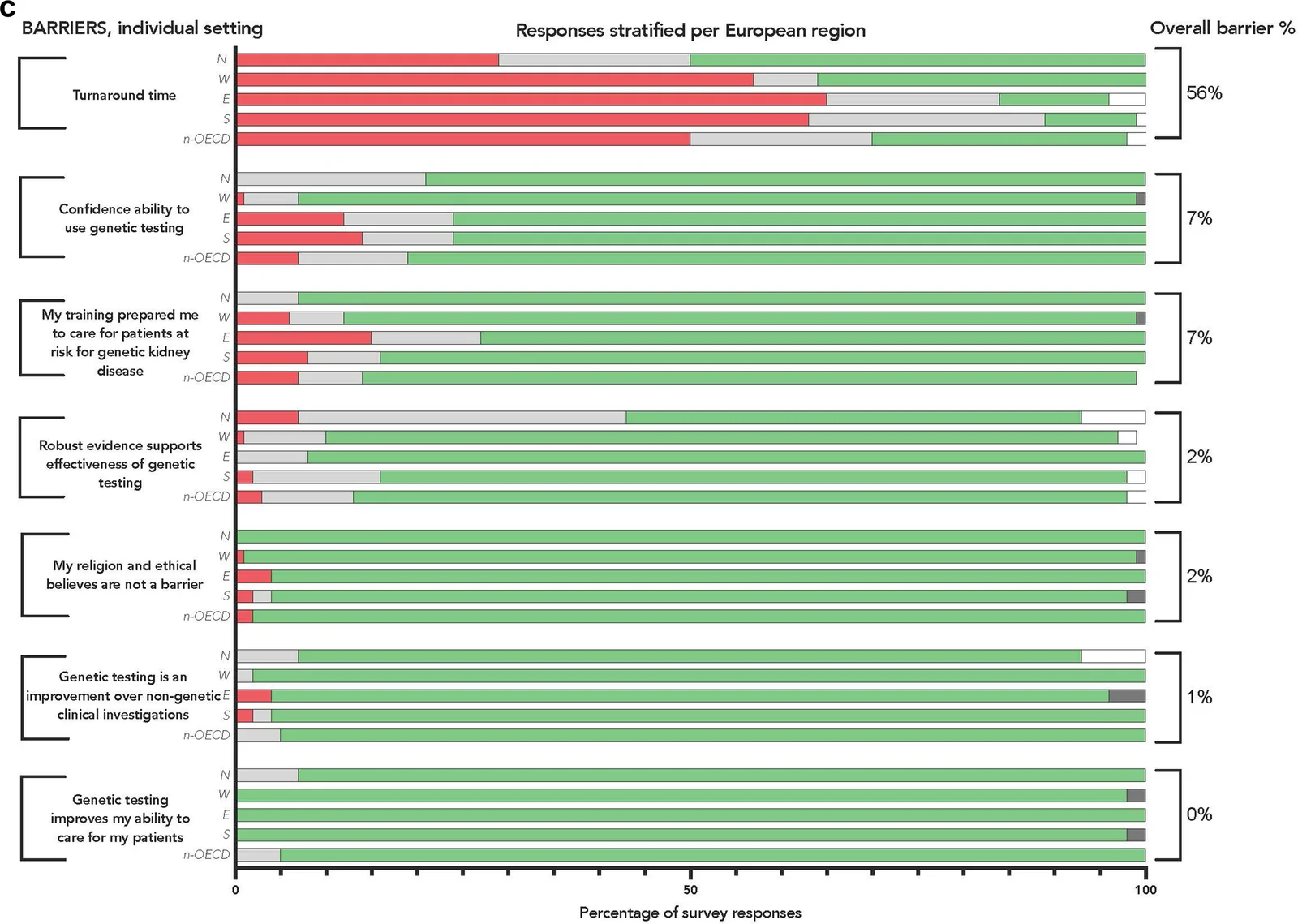
(a) Responses to the “outer setting” barrier statements regarding the implementation of genetic testing for suspected hereditary kidney disease, stratified in 5 European regions—North (*n* = 14), West (*n* = 86–87), East (*n* = 26–27), South (*n* = 52), and non-OECD countries (*n* = 42). Responses are categorized as red (disagreed, indicating a barrier), light grey (neutral), green (agreed, indicating no barrier, white (I don’t know), and dark grey (NA). (b) Responses to the “inner setting” barrier statements regarding the implementation of genetic testing for suspected hereditary kidney disease, stratified in 5 European regions—North (*n* = 14), West (*n* = 86), East (*n* = 26), South (*n* = 51–52), and non-OECD countries (*n* = 42). Responses are categorized as red (disagreed, indicating a barrier), light grey (neutral), green (agreed, indicating no barrier, white (I don’t know), and dark grey (NA). (c) Responses to the “individual setting” barrier statements regarding the implementation of genetic testing for suspected hereditary kidney disease, stratified in 5 European regions—North (*n* = 14), West (*n* = 85), East (*n* = 26), South (*n* = 51), and non-OECD countries (*n* = 40–41). Responses are categorized as red (disagreed, indicating a barrier), light grey (neutral), green (agreed, indicating no barrier, white (I don’t know), and dark grey (NA).

### Relevance of Genetic Testing for Different Diseases

All participants assigned high importance to diagnostic genetic testing for different suspected CKD diagnoses (Supplementary Table S10). For adult care, importance scores ranged from 72% (76/106) for congenital anomalies of the kidney and urinary tract and rare causes of hypertension to 95% (101/106) for tubulopathies. For pediatric care, scores ranged from 90 of 135 67% (90/135) for congenital anomalies of the kidney and urinary tract to 98% (132/135) for glomerulopathies, tubulopathies, nephronophthisis, and other ciliopathies. For prenatal care, importance for CAKUT scored 41% (21/51) versus 80% (41/51) for ciliopathies.

### Factors Associated With Barrier Experience

Location of genetic testing was univariably associated with barrier experience (own hospital testing OR 0.4 [0.2, 0.8], testing in commercial lab other country OR 4.9 [1.9, 12.5]) (Table 3 shows univariable and multivariable logistic regression analyses of the outcome variable “experiencing barriers to the (further) implementation of genetic testing”). Being a member of the ERKNet was associated with lower barrier experience in univariable analysis (OR 0.6 [0.3, 0.99]).

**Table 3:** Predictors of experiencing barriers to genetic testing

Predictor	Barrier experience (*n* barriers/total n variable)	Univariable OR (95% CI)	Multivariable OR (95% CI)
Univariable analyses			
Profession			
Pediatric nephrologist	62/125 (50%)	1.0 (0.6, 1.7)	
Adult nephrologist	45/89 (51%)	1.07 (0.6, 1.8)	
Clinical geneticist	4/18 (22%)	0.3 (0.08, 0.83)	0.4 (0.1, 1.2)
Type of care			
Pediatric	61/134 (46%)	0.7 (0.4, 1.1)	
Adult	56/110 (51%)	1.1 (0.7, 1.9)	
Prenatal	24/54 (44%)	0.8 (0.4, 1.4)	
Location of diagnostic testing			
Own hospital	61/145 (42%)	0.4 (0.2, 0.8)	0.8 (0.4, 1.6)
Other hospital in own country	55/112 (49%)	1.0 (0.6, 1.6)	
Commercial lab in own country	37/59 (63%)	2.0 (1.1, 3.8)	1.7 (0.8, 3.5)
Commercial lab in other country	24/30 (80%)	4.9 (1.9, 12.5)	1.2 (0.3, 4.0)
Lab in other hospital, other country	21/27 (78%)	4.1 (1.6, 10.7)	1.8 (0.6, 5.5)
Only through research	11/12 (92%)	12.3 (1.6, 96.9)	2.2 (0.2, 20.7)
Region			
West	34/87 (39%)	0.5 (0.3, 0.9)	0.8 (0.4, 1.7)
East	17/27 (63%)	1.9 (0.8, 4.3)	
South	23/56 (41%)	0.6 (0.3, 1.2)	
Nordic	5/14 (36%)	0.5 (0.2, 1.7)	
Non-OECD	34/44 (77%)	4.5 (2.1, 9.7)	3.5 (1.2, 9.8)
Which party usually pays for genetic testing			
Patient/family	33/37 (89%)	11.4 (3.9, 33.6)	7.4 (2.3, 23.9)
Health insurance	35/70 (50%)	1.0 (0.6, 1.8)	
Public health service	67/144 (47%)	0.7 (0.4, 1.2)	
Hospital/local budget	35/53 (66%)	2.4 (1.3, 4.6)	2.6 (1.2, 5.6)
Commercial company for free	7/11 (64%)	1.8 (0.5, 6.4)	
Research funded	33/45 (73%)	3.5 (1.7, 7.3)	2.6 (1.1, 6.3)
*ERKNet*			
ERKNet member/affiliated center	60/136 (44%)	0.6 (0.3, 0.99)	1.1 (0.5, 2.4)
EU-member, yes	88/188 (47%)	0.5 (0.3, 1.1)	

After inclusion of univariable significant variables into the multivariable logistic regression model, 4 variables remained associated with experiencing barriers—originating from a non-OECD region (OR 3.5 [1.2, 9.8]) and usually paying for genetic testing—i) the patient or family (OR 7.4 [2.3, 23.9]), ii) the hospital or local budget (OR 2.6 [1.2, 5.6]), and iii) research funded (OR 2.6 [1.1, 6.3]). Subgroup analyses on OECD-regions only (North, West, East, and South,) are shown in Supplementary Table S11. Next to the associations above, the latter analysis showed 2 additional associations with barrier experience–i)originating from an Eastern region was associated with higher barrier experience (only univariably significant, OR 2.6 [1.1, 6.1]) and ii) providing pediatric care was associated with lower barrier experience (OR 0.4 [0.2, 0.9] which did remain significant in multivariable analysis. Supplementary Tables S12–S14 and Supplementary Figure S9A–D stratify barriers per profession, showing that adult nephrologists encounter barriers across multiple domains more frequently than pediatric nephrologists and clinical geneticists.

### Comparison of Survey Responses With Objective Data From ERKReg

In the ERKReg database, the Southern and Western European regions showed a high, population-adjusted number of patients with an indication for genetic testing (data on genetic testing from the ERKReg database are shown in Supplementary Tables S1, S15, and S16 and Supplementary Figure S10, including an overview of patients with genetically tested rare ciliopathy per European country). The lowest number was seen in non-OECD countries. Of patients with an indication for genetic testing, the percentage tested patients varied from 2222 of 4089 (54%) to 29 of 38 (76%).

## Discussion

### Key Results

In this study involving pediatric and adult nephrologists, as well as clinical geneticists across 39 European countries, we investigated the accessibility of and barriers to the implementation of genetic testing for patients with suspected hereditary kidney disease. This study identified 4 key findings:

First, 50% of participants experienced barriers in daily practice. Overall, the median severity score for barriers experienced in daily practice was moderate—4.0 (IQR 4) on a scale of 1–10. However, when stratified by different European regions, non-OECD countries showed significantly higher severity scores (6.8 [IQR 3.6]; *P* < 0.001) compared with other regions.

Second, the most frequently reported barriers were lacking clear professional group guidelines, concerns about posttest insurability of patients, missing a clear protocol, high costs of testing, insufficient resources, and turnaround time (Table 2). Nonetheless, motivation for (further) implementation of genetic testing was high; 97% valued genetic testing to improve care.

Third, ranking of most reported barriers by European region revealed a gradient, from least barrier experience in Nordic regions, followed by West, South, East, to greatest in non-OECD countries.

Fourth, originating from a non-OECD or Eastern region, lack of external funding, providing nonpediatric care, location of diagnostic testing, and not being an ERKNet member (both univariable) were associated with higher barrier experience in regression analysis.

In our study, clinicians connected to ERKNet experienced less barriers than non-ERKNet connected centers (44% vs. 58%). To become part of an ERN, such as ERKNet, centers must meet European Union Committee of Experts on Rare Diseases criteria of demonstrated expertise, multidisciplinary approach, quality assurance, research contribution, education, and (inter)national collaboration.^27^ The lower perceived barriers suggest that the concept of European Reference Centers is functioning effectively. This supports the importance of referring patients to such centers, where comprehensive diagnostic services are available.

Furthermore, the ERKReg data set shows us that barriers appear to lie in reaching expert centers, with patients from non-OECD and Eastern countries being underrepresented among those with a genetic testing indication compared with Southern and Western countries. In contrast, testing rates among those seen at expert centers are relatively high (54%–76%). However, EU member states must designate expert centers linked to ERNs. In non-OECD countries, centers can join ERKReg only voluntarily. This likely leads to underrepresentation of capable hospitals in those regions. Therefore, the gaps suggest—but do not prove—access barriers. Thus, strengthening referral pathways to expert centers is especially urgent in underrepresented regions.

In addition, previous research revealed disparities in clinical genetics staffing across Europe, warning for impact on rare disease care and ERN eligibility.^16^ To address this concern, the European Rare Diseases Research Alliance (with ERKNet as a partner) has recently established “National Mirror Groups,” a partnership that works to align national and international rare disease research strategies, particularly in countries that are behind in developing and implementing national plans.^28^

Although in most European countries genetic discrimination is partially prohibited by law, especially participants in Western European countries reported concerns of posttest insurability. It is likely that these concerns primarily pertain to presymptomatic patients, mildly affected individuals or carriers, as patients with symptomatic (severe) disease are often already required to disclose their disease status when applying for insurance. This could explain the higher concerns in the West, where access to genetic testing is already quite accessible for a range of indications and genetic testing is mostly financed by health insurance or government funding. Nonetheless, we know from the literature as well as our own experience, that misconceptions about obtaining life insurance or mortgages are common, and both patients and clinicians often are unaware of the applicable regulations.^29^^,^^30^

Recent international recommendations on the use of genetic testing in diagnosing CKD have expressed the need for developing guidelines for referring nephrology patients to genetic counseling, testing, and reproductive counseling.^9^ In addition, the creation of clinical decision support tools was advised.^1^ These recommendations align with the need clinicians reported in this survey. Developing (inter)national guidelines, coupled with effective communication to the professionals implementing them, could help overcome this barrier. These guidelines should be integrated into well-defined protocols and workflows to facilitate genetic testing for patients with suspected genetic kidney disease in individual hospitals.

When asked whether the further implementation of genetic testing should be prioritized over other initiatives to improve care for patients with CKD, 29 of 42 (69%) of participants in non-OECD countries agreed versus 3 of 14 (21%) in Nordic countries. It is conceivable that the Nordic respondents are less eager to prioritize as their survey responses suggest that genetic testing is already quite accessible. Of the non-OECD countries included in this survey, 11 of 15 (73%) are either already members of the EU or have been officially nominated for membership. Therefore, it is relevant for the EU to address not only the barriers in OECD regions (South, East, West, and North) but also in non-OECD countries.

### Comparison to Other Genomic Barrier Studies

Previous studies on barriers to implementing genetic testing for kidney disease in the United States and Australia identified high costs, limited availability, inadequate staffing levels, funding, and educational resources as primary barriers.^13^^,^^14^ Among others, a study among general Dutch nephrologists also identified low genetic literacy as a barrier.^31^ Most of these findings align with our survey results, except for the need for education. Our predominantly expert sample likely explains the high reported confidence levels, potentially underestimating the educational needs for using genomics in CKD among the broader clinical community. Furthermore, use of the CFIR model in other genetic fields (i.e., pharmacogenetics and pediatric rare disease) showed a similar trend with the highest barrier scores identified in the costs, funding of genetic testing, lack of staffing, infrastructure, and in the inner setting (i.e., technical hurdles).^20^^,^^32^^,^^33^

### Strengths & Limitations

This is the first study investigating barriers to the (further) implementation of genetic testing for patients with suspected hereditary kidney disease among pediatric and adult nephrologists and clinical geneticists in Europe. Although disparities in access to genetic testing in Europe were described previously, our work systematically identifies underlying implementation barriers specified to the nephrology setting and links these to inequities across regions. We adapted questions from previous surveys investigating physician-reported barriers to implementing genomic testing, using the CFIR model.^13^^,^^20^ Applying the CFIR framework—also widely used in non-nephrology contexts—and observing similar patterns, supports the robustness and generalizability of our findings. Also, we used the ERKReg database as a valuable proxy for real-world practice, with data from 30,000 patients across Europe. Despite low sample size in some countries, the East–West gradient was objectified with this real-world data.

Despite our efforts to reach a diverse sample of clinicians throughout Europe, participation was voluntary, and it is likely that those with a particular interest or expertise in the subject were more inclined to respond to the invitation distributed through professional organizations' mailing lists. This selection bias toward a predominant expert pediatric nephrology sample (86% academics, 59% connected to ERKNet, and 52% pediatric nephrologists) increased confidence in the results, as their specialized knowledge ensured informed responses. However, a limitation of this expert sample is that the findings may underestimate barriers in general settings, making them less generalizable to broader clinical contexts. The overrepresentation of pediatric nephrologists may reflect greater engagement and more advanced skills development in this area. In contrast, engagement among adult nephrologists appears to be at an earlier stage, suggesting substantial opportunities for further implementation and capacity building. Furthermore, although comparisons are ideally made on a country-by-country basis, interpreting results by comparing 39 distinct European countries individually is challenging. To address this, we grouped countries into regions. Despite efforts to group European regions with similar welfare state typologies, not all countries in one region have identical health care systems and infrastructures. In addition, uneven per country representation has affected the reported regional percentages.

## Conclusion

We identified specific barriers that must be addressed both at a European level and within individual European countries and regions. As virtually all respondents consider genetic testing important for clinical practice, addressing these barriers becomes even more urgent. To address clinicians' needs, we recommend development of (inter)national guidelines on implementation of genetic testing into daily practice for patients with suspected CKD, effectively communicating these guidelines to health care professionals, and integrating them into hospital protocols (Table 4, recommendations). Especially for Southern, Eastern, and non-OECD countries, EU or other funding could address challenges, such as limited resources, high testing costs, and inadequate access to technical facilities. Investing in a good infrastructure to reach and communicate with centralized, specialized laboratories could enhance equitable access to advanced genetic services. In addition, where clinical geneticists are scarce, establishing resident training programmes deserves top priority. These recommendations could promote equity between EU countries and reduce inequity in access to genetic care for patients with kidney disease. Future research should focus on nonexpert samples to capture barriers within more general care settings.

**Table 4:** Recommendations

Recommendations
As 97% of respondents consider genetic testing important to improve clinical care, there is urgence to address barriers by:
• Development of (inter)national guidelines on implementation of genetic testing into daily practice for patients with CKD
• Effectively communicating these guidelines to healthcare professionals and integrate guidelines into hospital protocols
• Promoting government or regulated insurance funding for diagnostic genetic testing over selfpayment, institutional funding, or research grants
• Additional funding to address challenges such as limited resources and inadequate technical facilities
• Investing in a good infrastructure to reach and communicate with centralized, specialized laboratories to enhance equitable access to advanced genetic services
• Resident training programs for clinical geneticists should be prioritized in countries where they are underrepresented

## Disclosure

All the authors have no conflicts to disclose.

## Supplementary Material

Supplementary File (PDF) **Supplementary Material S1.** Survey on the accessibility and barriers to the (further) implementation of genetic testing for patients with suspected hereditary kidney disease in Europe. **Figure S1.** Flowchart survey development and dissemination. **Figure S2.** Graphic overview baseline characteristics survey participants. **Figure S3.** Visual overview: geographic map of Europe displaying the 5 different European regions of the survey. **Figure S4.** (A) Survey participants working in a setting connected to ERKNet, overall and stratified per European region. (B) Survey participants receiving invitation via: overall and stratified per European region. **Figure S5.** (A) Who does pretest counseling, all survey participants and stratified per region. (B) Who is involved in interpretation of genetic test results, all survey participants and stratified per region. (C) Locations diagnostic testing for patients with suspected hereditary kidney disease, all survey participants and stratified per European region. (D) Who does posttest counseling, all survey participants and stratified per region. **Figure S6.** Party usually paying for genetic testing, all survey participants and per European region. **Figure S7.** Turnaround times for genetic test results, for urgent and non-urgent situations per European region. **Figure S8.** Responses to the “process setting” barrier statements regarding the implementation of genetic testing for suspected hereditary kidney disease, stratified in 5 European regions. **Figure S9.** (A) Responses to the “outer setting” barrier statements regarding the implementation of genetic testing for suspected hereditary kidney disease, stratified for different professions. (B) Responses to the “inner setting” barrier statements regarding the implementation of genetic testing for suspected hereditary kidney disease, stratified for different professions. (C) Responses to the “individual setting” barrier statements regarding the implementation of genetic testing for suspected hereditary kidney disease, stratified for different professions. (D) Responses to the “process setting” barrier statements regarding the implementation of genetic testing for suspected hereditary kidney disease, stratified for different professions. **Figure S10.** ERKReg prevalence genetic testing indications per 1000 000 inhabitants per European region and percentage truly tested patients with an indication for genetic testing. **Table S1.** European Rare Kidney disease Registry (ERKReg) data, list of included diagnoses and genetic testing indications yes/no. **Table S2.** Overview countries of origin survey participants, including stratification into European regions. **Table S3.** Logistic aspects, for total survey sample and stratified per European region. **Table S4.** Barrier statements and responses from all survey participants. **Table S5.** Barrier statements and responses from Western countries. **Table S6.** Barrier statements and responses from Eastern countries. **Table S7.** Barrier statements and responses from Southern countries. **Table S8.** Barrier statements and responses from Northern countries. **Table S9.** Barrier statements and responses from non-OECD countries. **Table S10.** Added value of genetic testing for different suspected CKD diagnoses according to survey participants. **Table S11.** Logistic regression subgroup analyses: OECD-regions. **Table S12.** Barrier statements and responses from pediatric nephrologists. **Table S13.** Barrier statements and responses from adult nephrologists. **Table S14.** Barrier statements and responses from clinical geneticists. **Table S15.** European Rare Kidney disease Registry (ERKReg) data, details on included genetically tested individuals. **Table S16.** European Rare Kidney disease Registry (ERKReg) data, details on included genetically tested individuals, per European Region. Checklist for Reporting of Survey Studies (CROSS).

## Footnotes

Supplementary File (PDF)
**Supplementary Material S1.** Survey on the accessibility and barriers to the (further) implementation of genetic testing for patients with suspected hereditary kidney disease in Europe. **Figure S1.** Flowchart survey development and dissemination. **Figure S2.** Graphic overview baseline characteristics survey participants. **Figure S3.** Visual overview: geographic map of Europe displaying the 5 different European regions of the survey. **Figure S4.** (A) Survey participants working in a setting connected to ERKNet, overall and stratified per European region. (B) Survey participants receiving invitation via: overall and stratified per European region. **Figure S5.** (A) Who does pretest counseling, all survey participants and stratified per region. (B) Who is involved in interpretation of genetic test results, all survey participants and stratified per region. (C) Locations diagnostic testing for patients with suspected hereditary kidney disease, all survey participants and stratified per European region. (D) Who does posttest counseling, all survey participants and stratified per region. **Figure S6.** Party usually paying for genetic testing, all survey participants and per European region. **Figure S7.** Turnaround times for genetic test results, for urgent and non-urgent situations per European region. **Figure S8.** Responses to the “process setting” barrier statements regarding the implementation of genetic testing for suspected hereditary kidney disease, stratified in 5 European regions. **Figure S9.** (A) Responses to the “outer setting” barrier statements regarding the implementation of genetic testing for suspected hereditary kidney disease, stratified for different professions. (B) Responses to the “inner setting” barrier statements regarding the implementation of genetic testing for suspected hereditary kidney disease, stratified for different professions. (C) Responses to the “individual setting” barrier statements regarding the implementation of genetic testing for suspected hereditary kidney disease, stratified for different professions. (D) Responses to the “process setting” barrier statements regarding the implementation of genetic testing for suspected hereditary kidney disease, stratified for different professions. **Figure S10.** ERKReg prevalence genetic testing indications per 1000 000 inhabitants per European region and percentage truly tested patients with an indication for genetic testing. **Table S1.** European Rare Kidney disease Registry (ERKReg) data, list of included diagnoses and genetic testing indications yes/no. **Table S2.** Overview countries of origin survey participants, including stratification into European regions. **Table S3.** Logistic aspects, for total survey sample and stratified per European region. **Table S4.** Barrier statements and responses from all survey participants. **Table S5.** Barrier statements and responses from Western countries. **Table S6.** Barrier statements and responses from Eastern countries. **Table S7.** Barrier statements and responses from Southern countries. **Table S8.** Barrier statements and responses from Northern countries. **Table S9.** Barrier statements and responses from non-OECD countries. **Table S10.** Added value of genetic testing for different suspected CKD diagnoses according to survey participants. **Table S11.** Logistic regression subgroup analyses: OECD-regions. **Table S12.** Barrier statements and responses from pediatric nephrologists. **Table S13.** Barrier statements and responses from adult nephrologists. **Table S14.** Barrier statements and responses from clinical geneticists. **Table S15.** European Rare Kidney disease Registry (ERKReg) data, details on included genetically tested individuals. **Table S16.** European Rare Kidney disease Registry (ERKReg) data, details on included genetically tested individuals, per European Region. Checklist for Reporting of Survey Studies (CROSS).
