# Supplementary material for: Access to Genetic Testing in Patients With Suspected Genetic Kidney Disease in Europe
Source: Kidney Int Rep. 2026 Sep 8;11(11):107070. doi: 10.1016/j.ekir.2026.107070 (PMC13692152; doi:10.1016/j.ekir.2026.107070)
Supplement: Supplementary file 1 [file mmc1.pdf]

SUPPLEMENTARY MATERIAL

Supplementary Material S1. [Survey on the accessibility and barriers to the (further) implementation of genetic testing for patients with suspected hereditary kidney disease in Europe]

Supplementary Figure S1. [Flowchart survey development and dissemination.]

Supplementary Figure S2. [Graphic overview baseline characteristics survey participants].

Supplementary Figure S3. [Visual overview: geographic map of Europe displaying the 5 different European regions of the survey.]

Supplementary Figure S4A. [Survey participants working in a setting connected to ERKNet, overall and stratified per European region.]

Supplementary Figure S4B. [Survey participants receiving invitation via: overall and stratified per European region.]

Supplementary Figure S5A. [Who does pretest counseling, all survey participants and stratified per region.]

Supplementary Figure S5B. [Who is involved in interpretation of genetic test results, all survey participants and stratified per region.]

Supplementary Figure S5C. [Locations diagnostic testing for patients with suspected hereditary kidney disease, all survey participants and stratified per European region.]

Supplementary Figure S5D. [Who does posttest counseling, all survey participants and stratified per region.]

Supplementary Figure S6. [Party usually paying for genetic testing, all survey participants and per European region.]

Supplementary Figure S7. [Turnaround times for genetic test results, for urgent and non-urgent situations per European region.]

Supplementary Figure S8. [Responses to the 'process setting' barrier statements regarding the implementation of genetic testing for suspected hereditary kidney disease, stratified in 5 European regions].

Supplementary Figure S9A. [Responses to the 'outer setting' barrier statements regarding the implementation of genetic testing for suspected hereditary kidney disease, stratified for different professions.]

Supplementary Figure S9B. [Responses to the 'inner setting' barrier statements regarding the implementation of genetic testing for suspected hereditary kidney disease, stratified for different professions.]

Supplementary Figure S9C. [Responses to the 'individual setting' barrier statements regarding the implementation of genetic testing for suspected hereditary kidney disease, stratified for different professions.]

Supplementary Figure S9D. [Responses to the 'process setting' barrier statements regarding the implementation of genetic testing for suspected hereditary kidney disease, stratified for different professions.]

Supplementary Figure S10. [ERKReg prevalence genetic testing indications per 1000 000 inhabitants per European region and percentage truly tested patients with an indication for genetic testing.]

Supplementary Table S1. [European Rare Kidney disease Registry (ERKReg) data, list of included diagnoses and genetic testing indications yes/no.]

Supplementary Table S2. [Overview countries of origin survey participants, including stratification into Europea n regions.]

Supplementary Table S3. [Logistic aspects, for total survey sample and stratified per European region.]

Supplementary Table S4. [Barrier statements and responses from all survey participants].

Supplementary Table S5. [Barrier statements and responses from Western countries].

Supplementary Table S6. [Barrier statements and responses from Eastern countries].

Supplementary Table S7. [Barrier statements and responses from Southern countries.]

Supplementary Table S8. [Barrier statements and responses from Northern countries.]

Supplementary Table S9. [Barrier statements and responses from non-OECD countries.]

Supplementary Table S10. [Added value of genetic testing for different suspected CKD diagnoses according to survey participants.]

Supplementary Table S11. [Logistic regression subgroup analyses: OECD-regions].

Supplementary Table S12. [Barrier statements and responses from pediatric nephrologists].

Supplementary Table S13. [Barrier statements and responses from adult nephrologists].

Supplementary Table S14. [Barrier statements and responses from clinical geneticists].

Supplementary Table S15. [European Rare Kidney disease Registry (ERKReg) data, details on incl uded genetically tested individuals.]

Supplementary Table S16. [European Rare Kidney disease Registry (ERKReg) data, details on included genetically tested individuals, per European Region.]

Cross checklist: Checklist for Reporting Of Survey Studies (CROSS).

(PDF)

Supplementary Material S1. Survey on the accessibility and barriers to the (further) implementation of genetic testing for patients with suspected hereditary kidney disease in Europe.

Available via: https://surfdrive.surf.nl/files/index.php/s/VNAM4nQjqEP1qGX

Supplementary Figure S1. Flowchart survey development and dissemination.

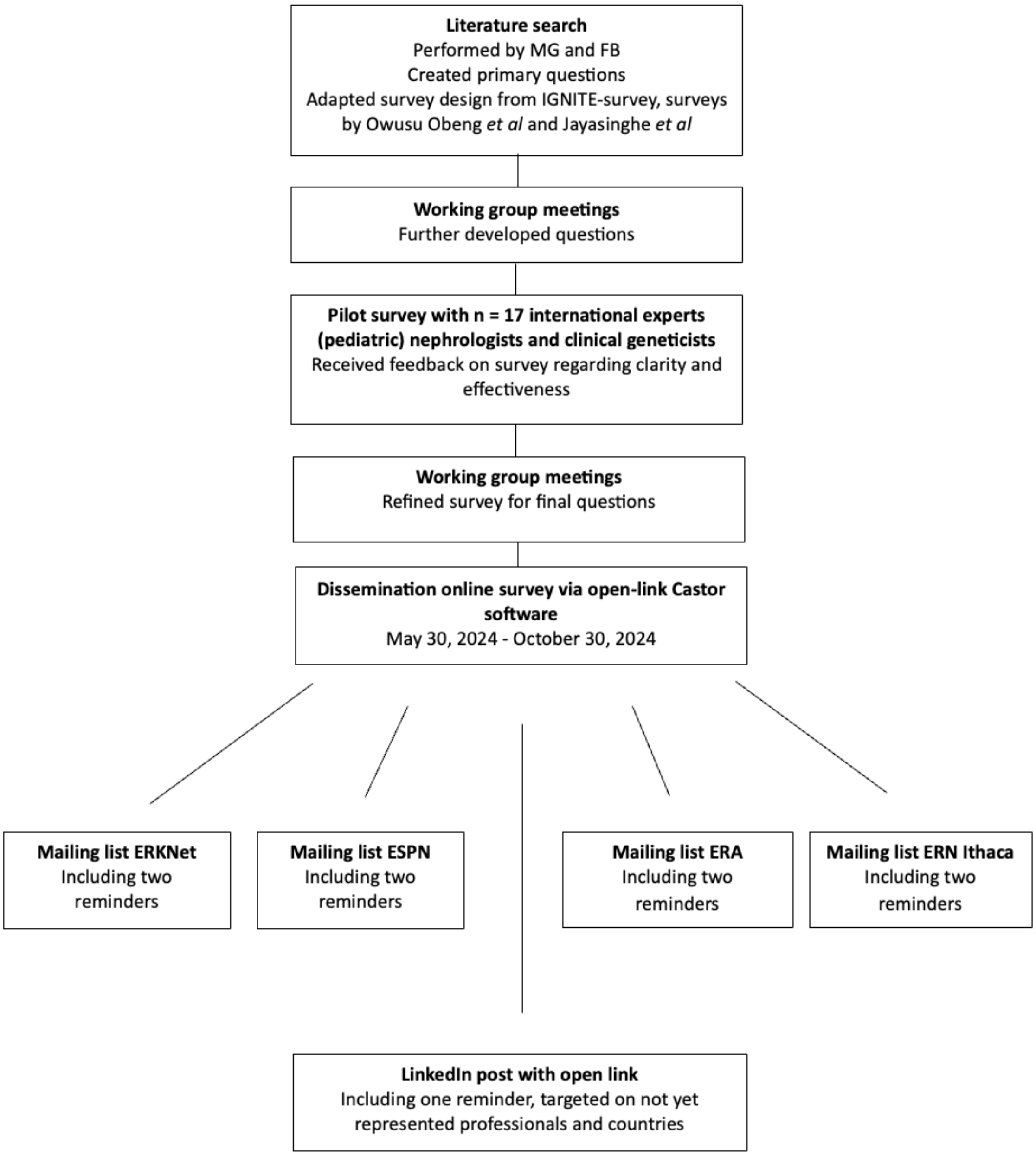

Supplementary Figure S2. Graphic overview baseline characteristics survey participants.

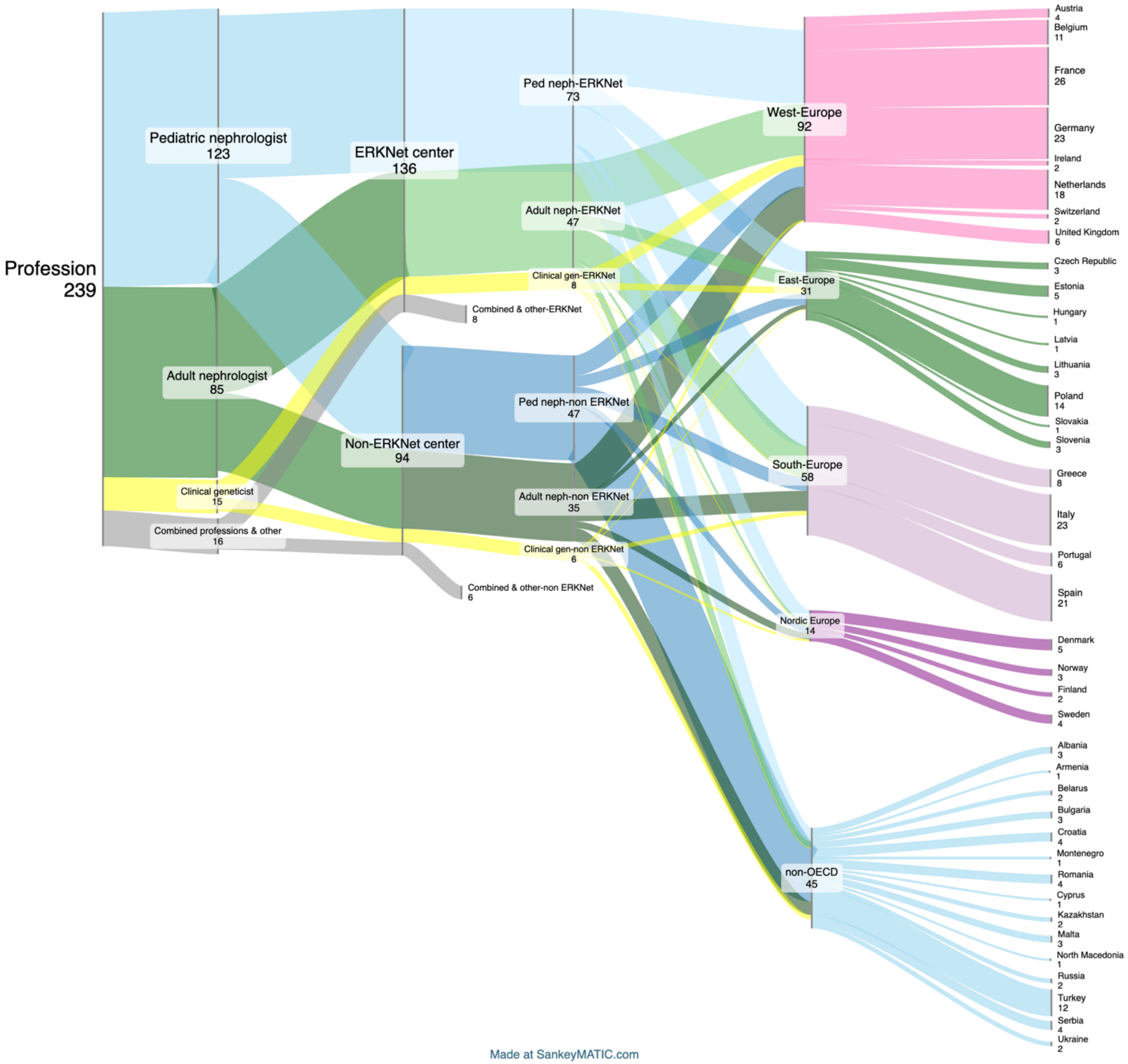

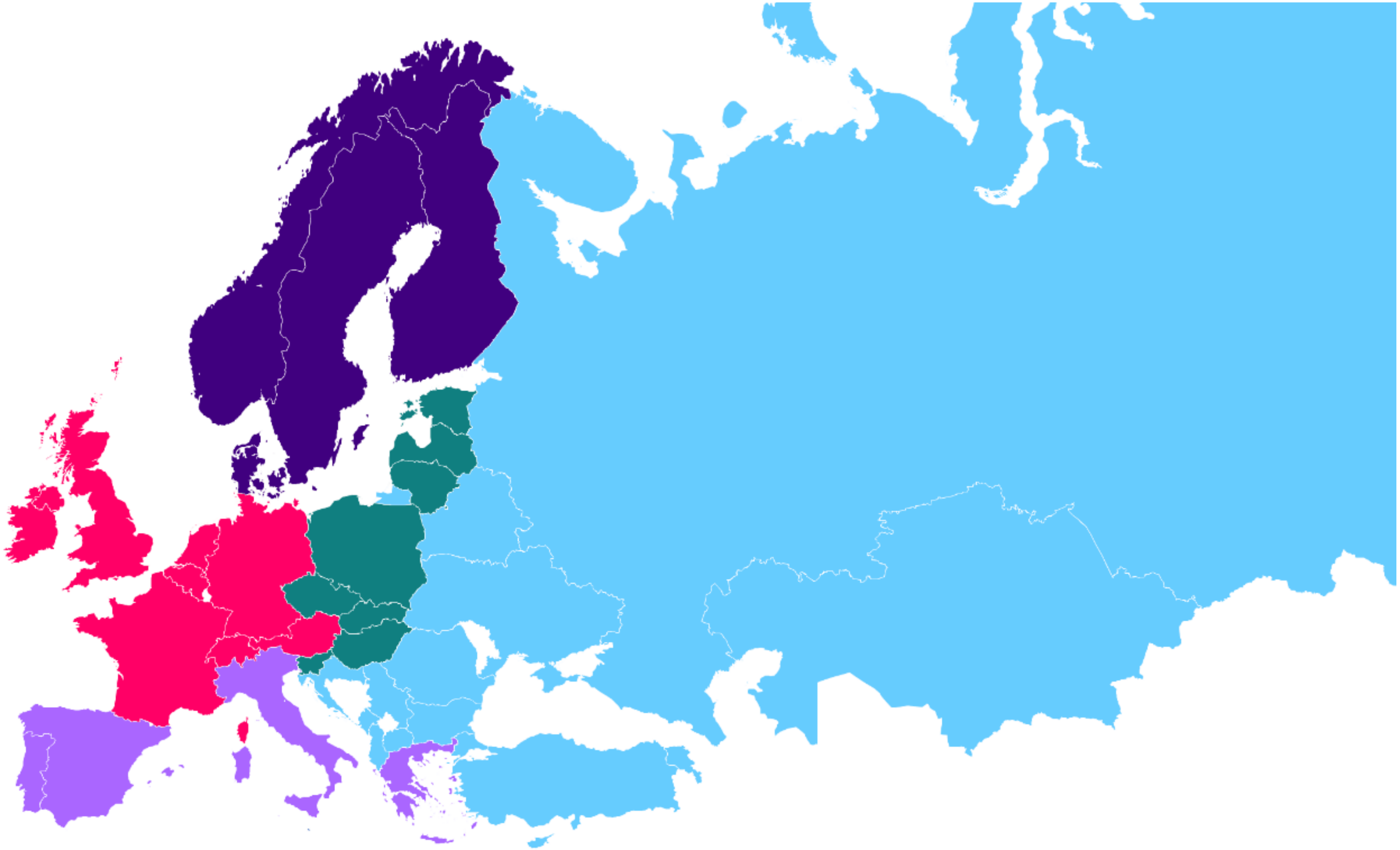

Supplementary Figure S3. Visual overview: geographic map of Europe displaying the 5 different European regions of the survey. Pink = West, Lilac = South, Green = East, Purple = North, Blue = non-OECD.

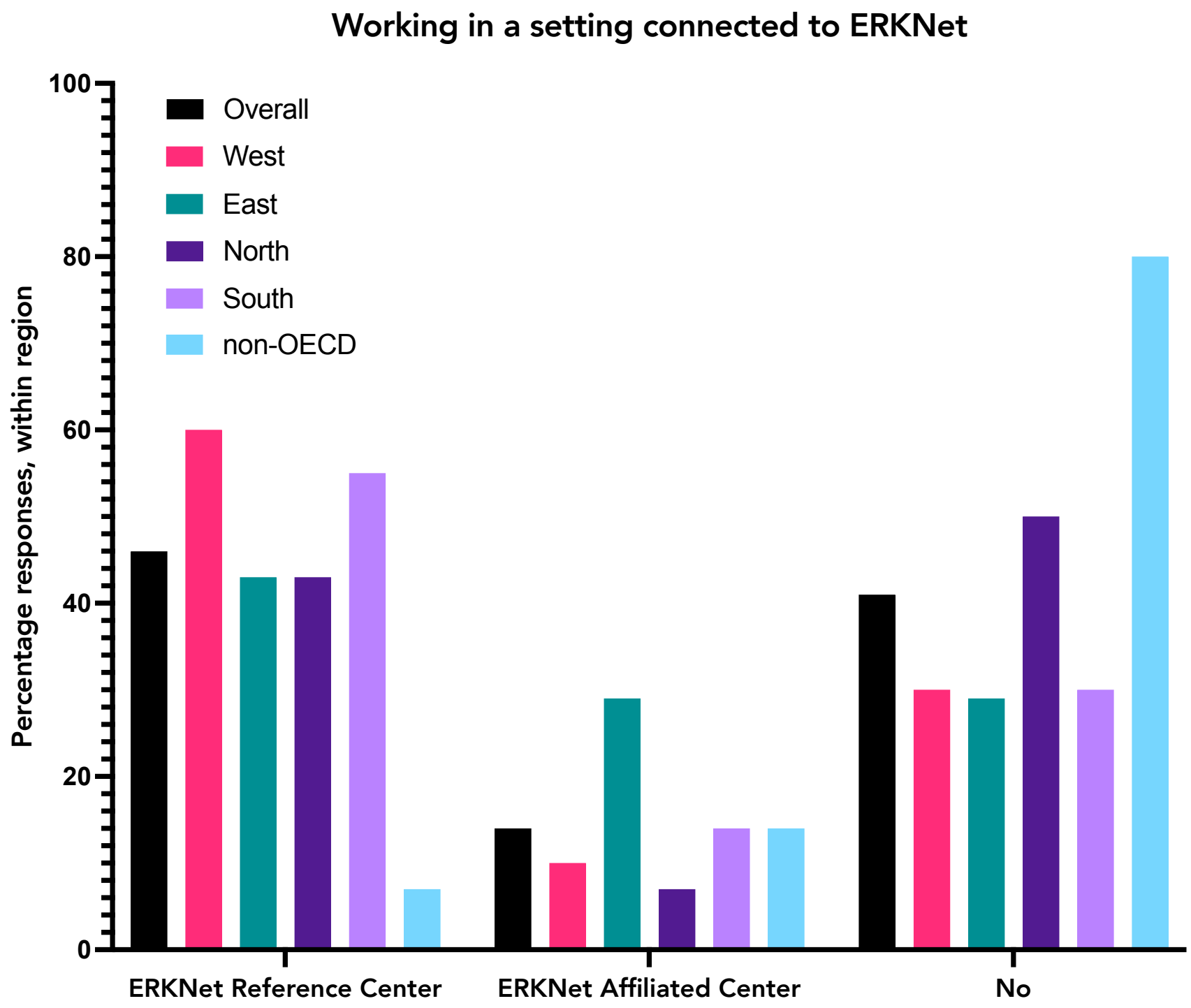

Supplementary Figure S4A. Survey participants working in a setting connected to ERKNet, overall and stratified per European region. Total N = 231, West N = 89, East N = 28, North N = 14, South N = 56, non-OECD N = 44.

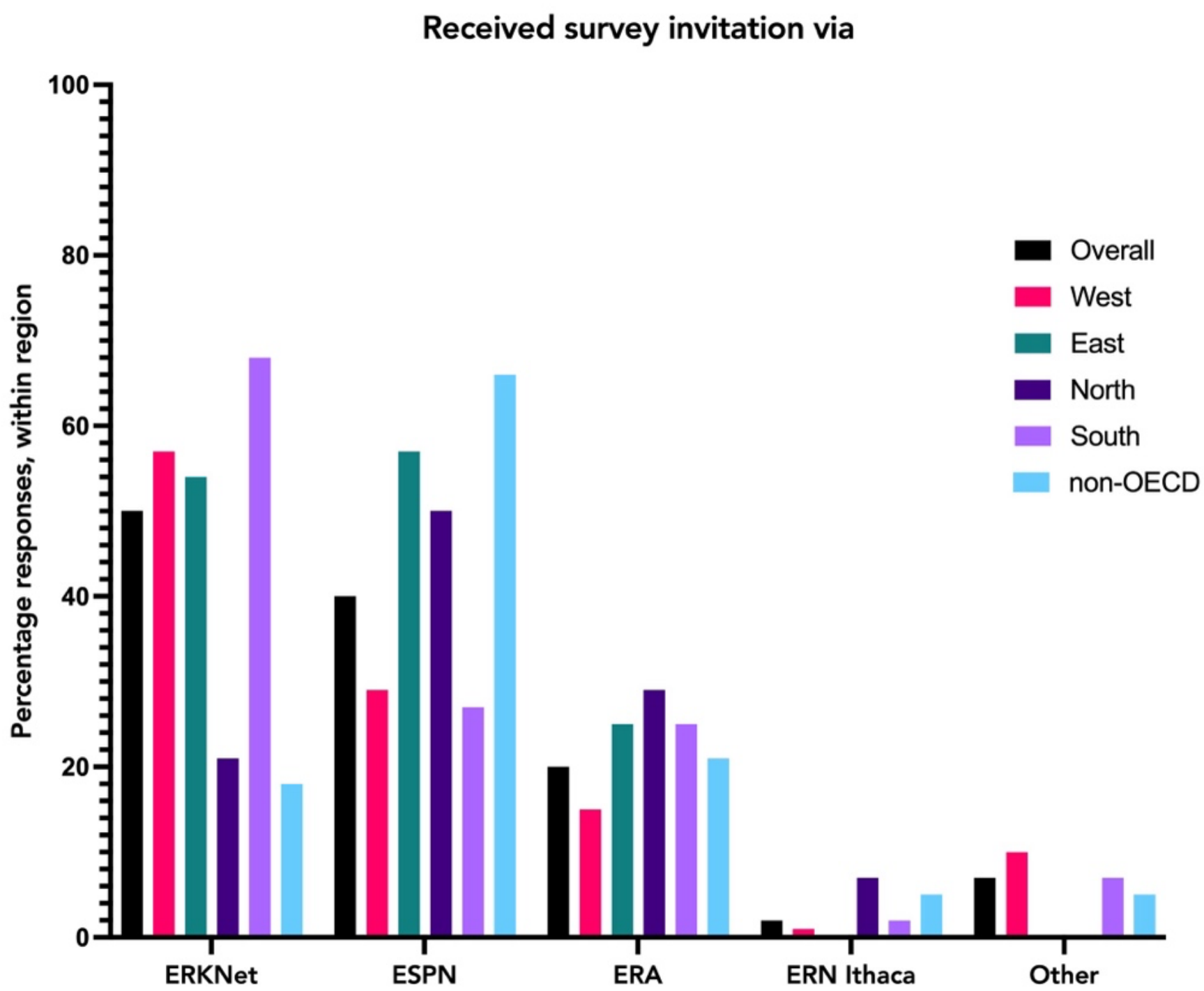

Supplementary Figure S4B. Survey participants receiving invitation via: overall and stratified per European region. Total N = 231, West N = 89, East N = 28, North N = 14, South N = 56, non-OECD N = 44.

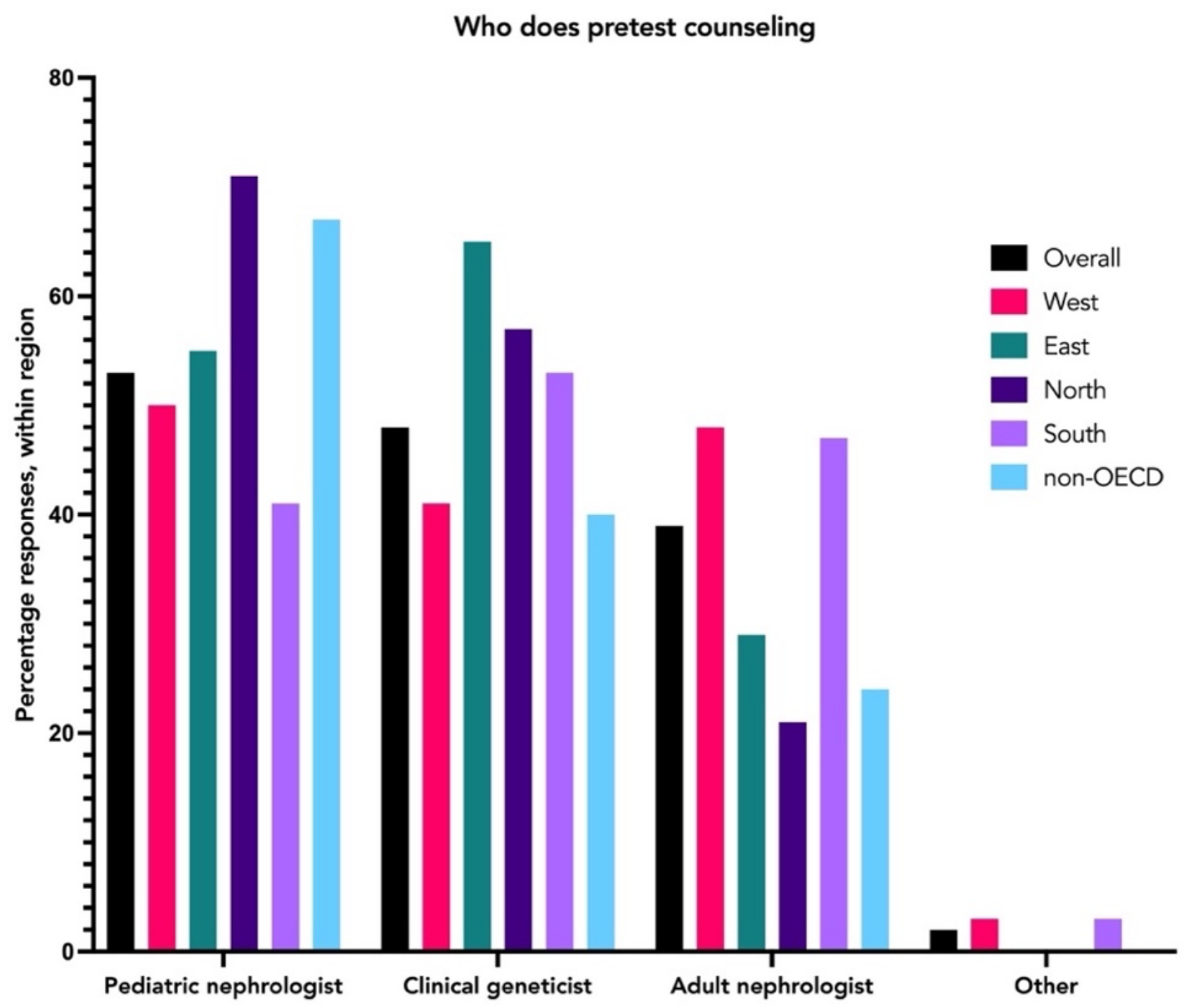

Supplementary Figure S5A. Who does pretest counseling, multiple options allowed. Shown for all survey participants and stratified per region. Total N = 240, West N = 92, East N = 31, North N = 14, South N = 58, non-OECD N = 45.

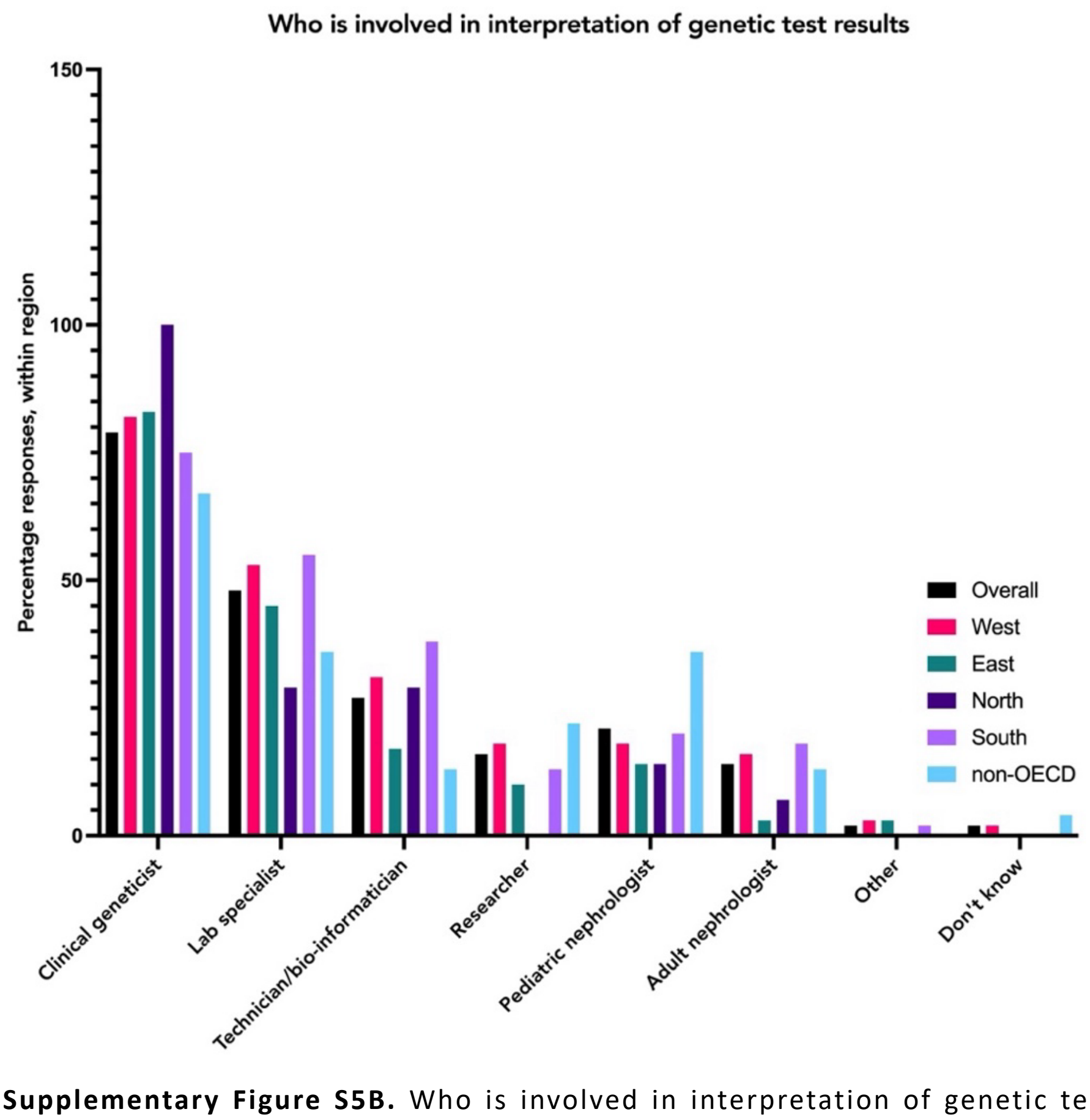

Supplementary Figure S5B. Who is involved in interpretation of genetic test results, multiple options allowed. Shown for all survey participants and stratified per region. Total N = 234, West N = 90, East N = 29, North N = 14, South N = 56, non-OECD N = 45.

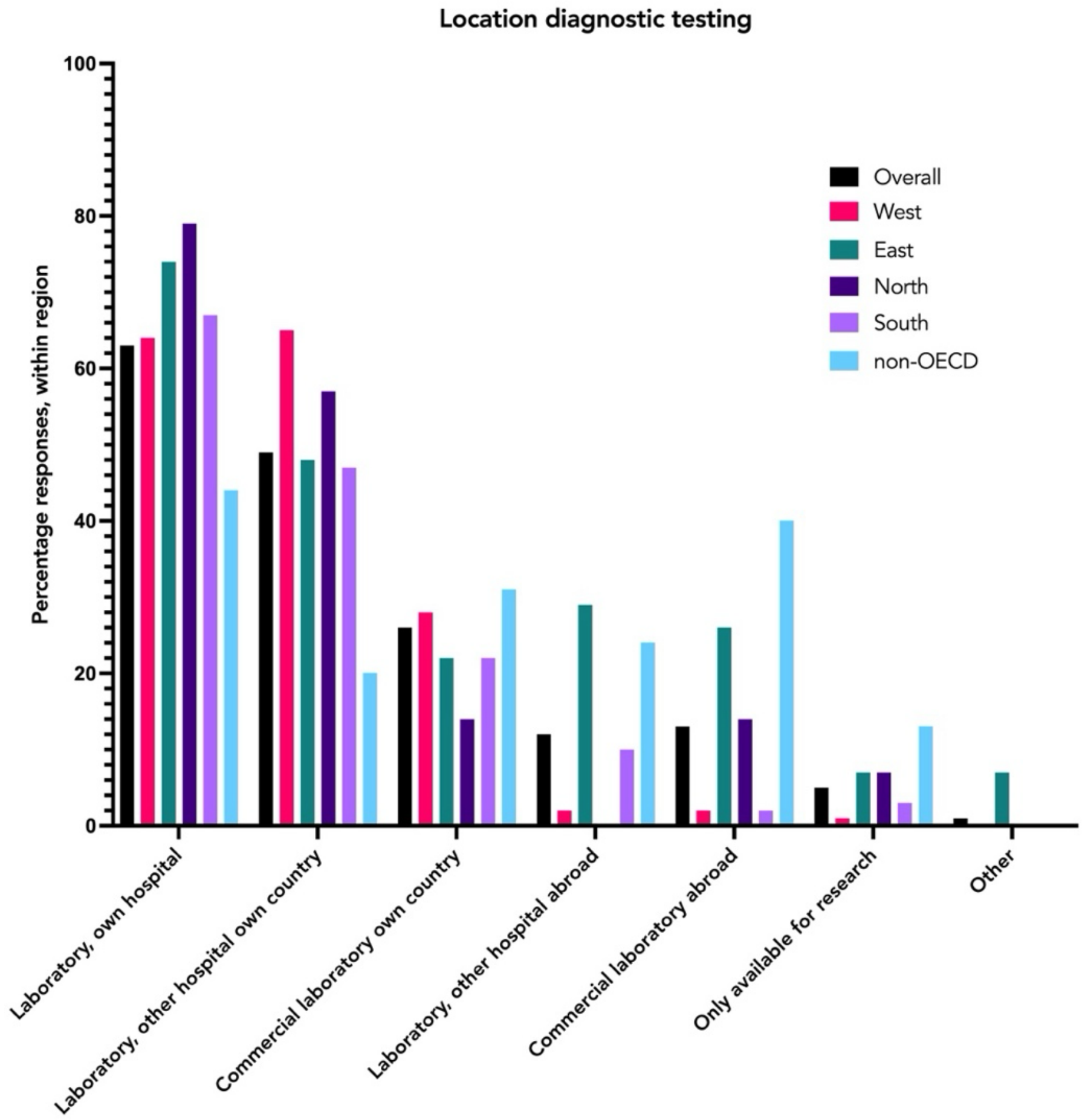

Supplementary Figure S5C. Locations diagnostic testing for patients with suspected hereditary kidney disease, multiple options allowed. Displayed for all survey participants (overall) and stratified per European region. Total N = 239, West N = 91, East N = 31, North N = 14, South N = 58, non-OECD N = 45.

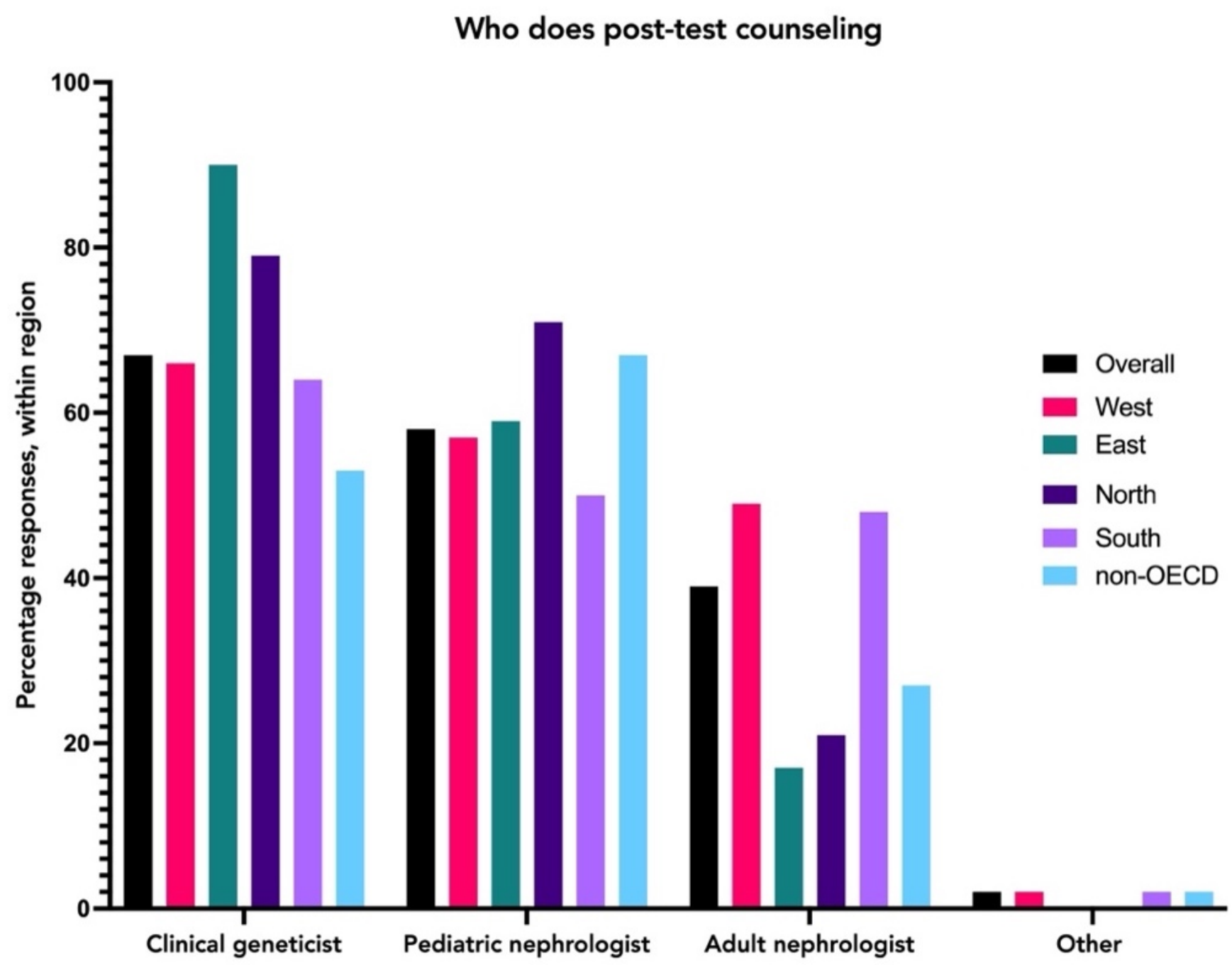

Supplementary Figure S5D. Who does posttest counseling, multiple options allowed. Shown for all survey participants and stratified per region. Total N = 233, West N = 89, East N = 29, North N = 14, South N = 56, non-OECD N = 45.

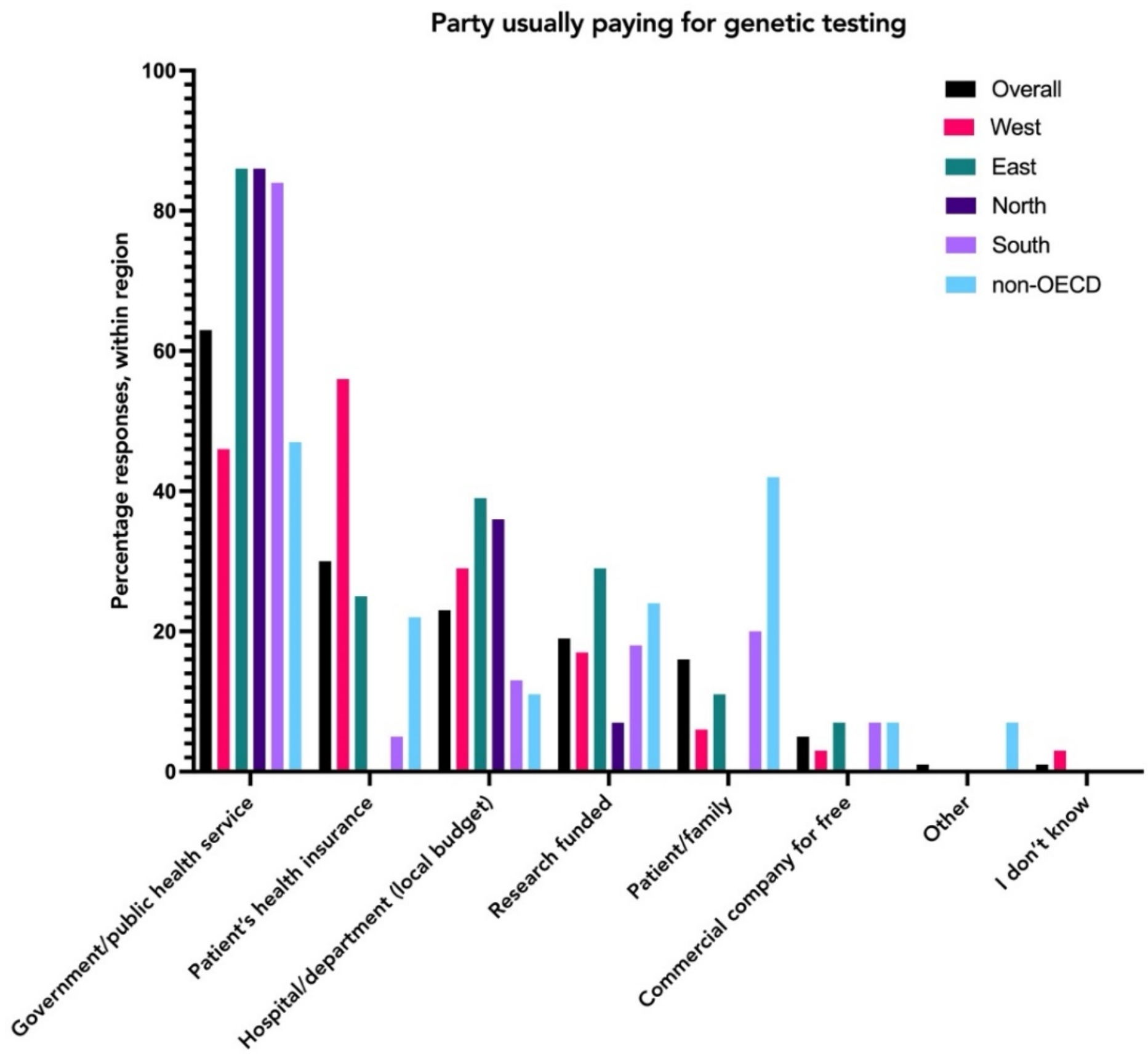

Supplementary Figure S6. Party usually paying for genetic testing, multiple options allowed. Displayed for all survey participants and per European region. Total N = 232, West N = 89, East N = 28, North N = 14, South N = 56, non-OECD N = 45.

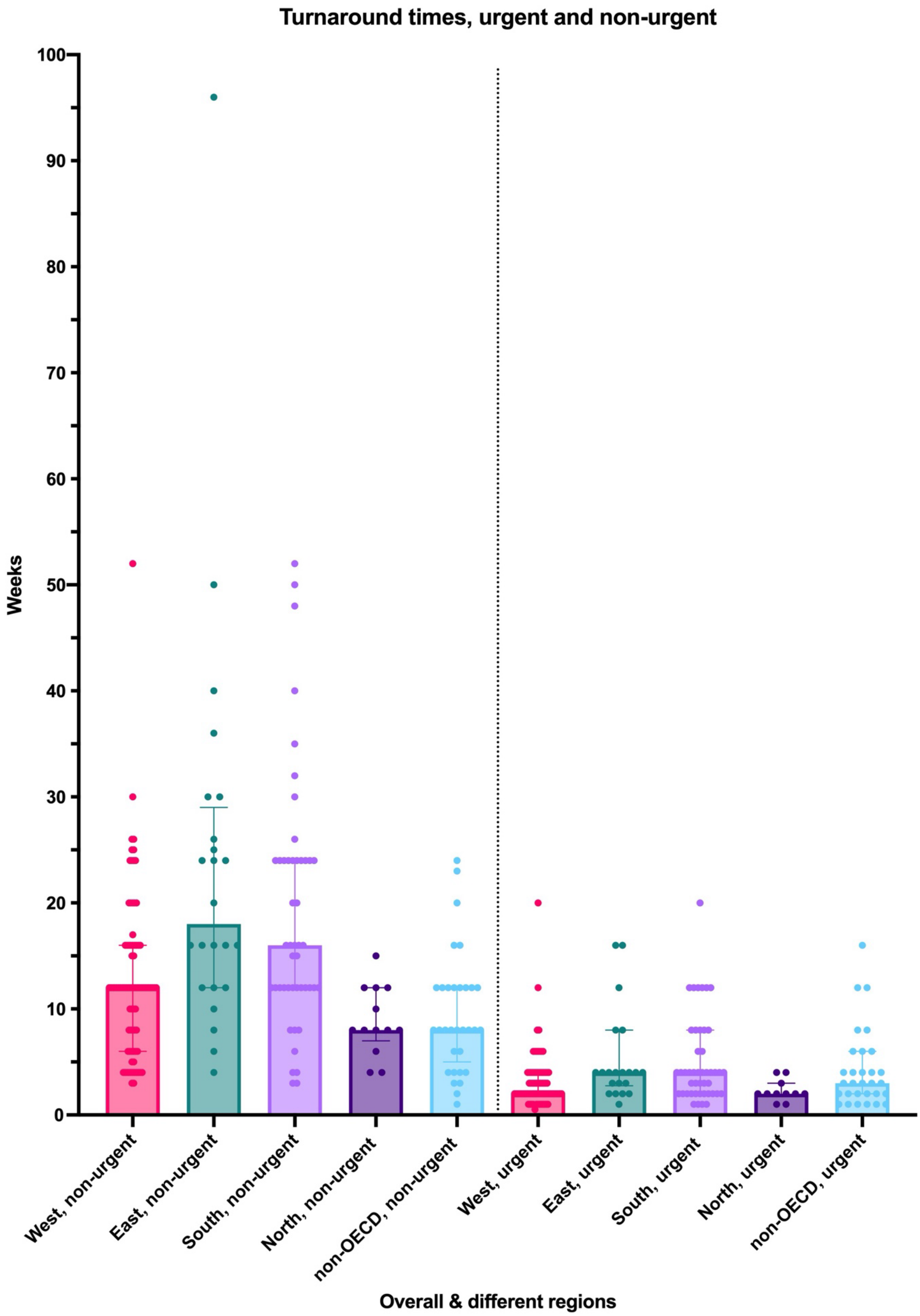

Supplementary Figure S7. Turnaround times for genetic test results, for urgent and non-urgent situations per European region. West: non-urgent n = 82, urgent = n 80. East: non-urgent n = 24, urgent n = 21. South: non-urgent n = 47, urgent n = 46. North: non-urgent n = 13, urgent n = 11. Non-OECD non-urgent n = 33, urgent n = 30.

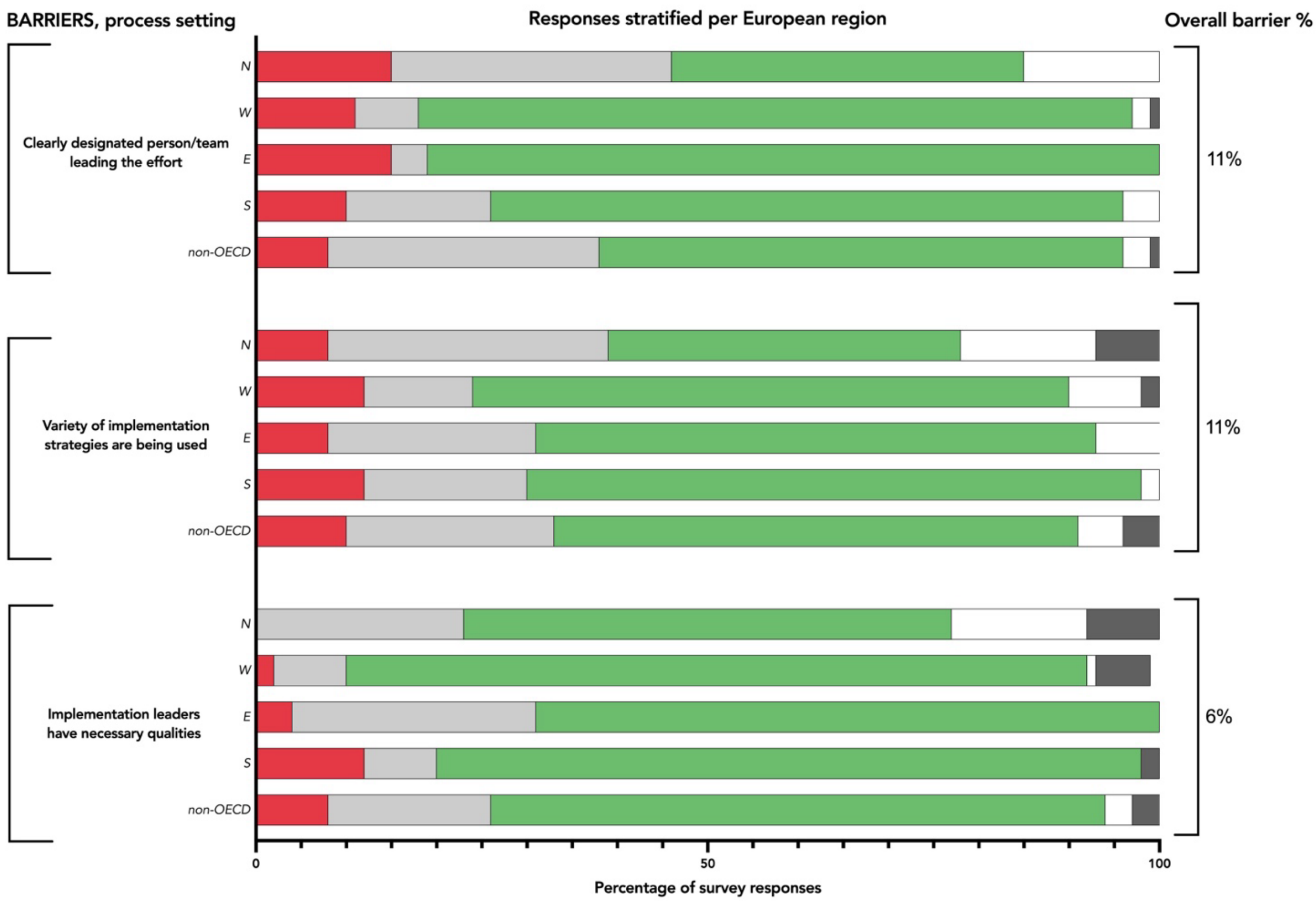

Supplementary Figure S8. Responses to the 'process setting' barrier statements regarding the implementation of genetic testing for suspected hereditary kidney disease, stratified in 5 European regions: West (N = 85), East (N = 26), South (N = 50), North (N = 13) and non-OECD countries (N = 40). Responses are categorized as red (disagreed, indicating a barrier), light grey (neutral), green (agreed, indicating no barrier, white (I don't know) and dark grey (NA).

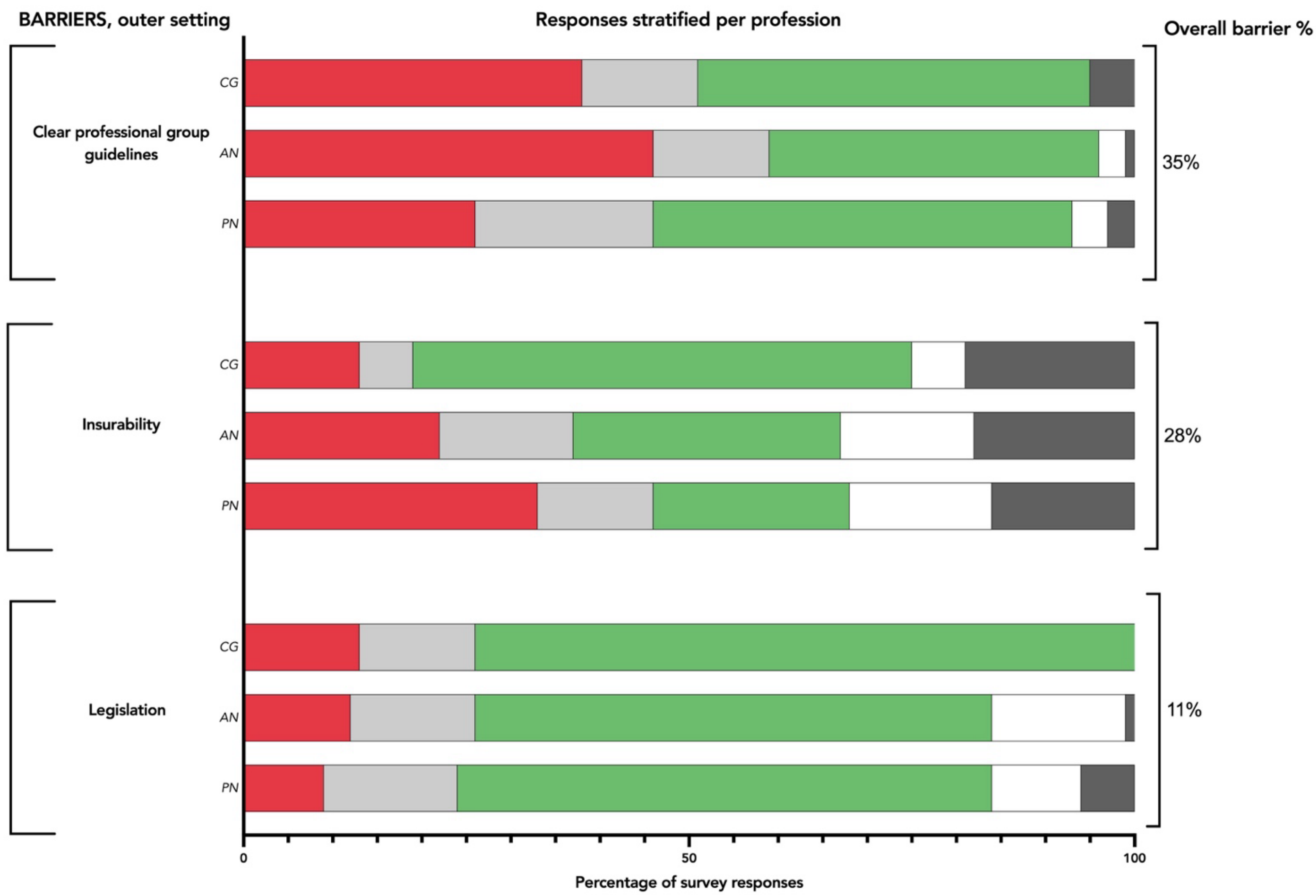

Supplementary Figure S9A. Responses to the 'outer setting' barrier statements regarding the implementation of genetic testing for suspected hereditary kidney disease, stratified for different professions. Pediatric nephrologists (pn), adult nephrologists (an) and clinical geneticists (cg). Responses are categorized as red (disagreed, indicating a barrier), light grey (neutral), green (agreed, indicating no barrier, white (I don't know) and dark grey (NA).

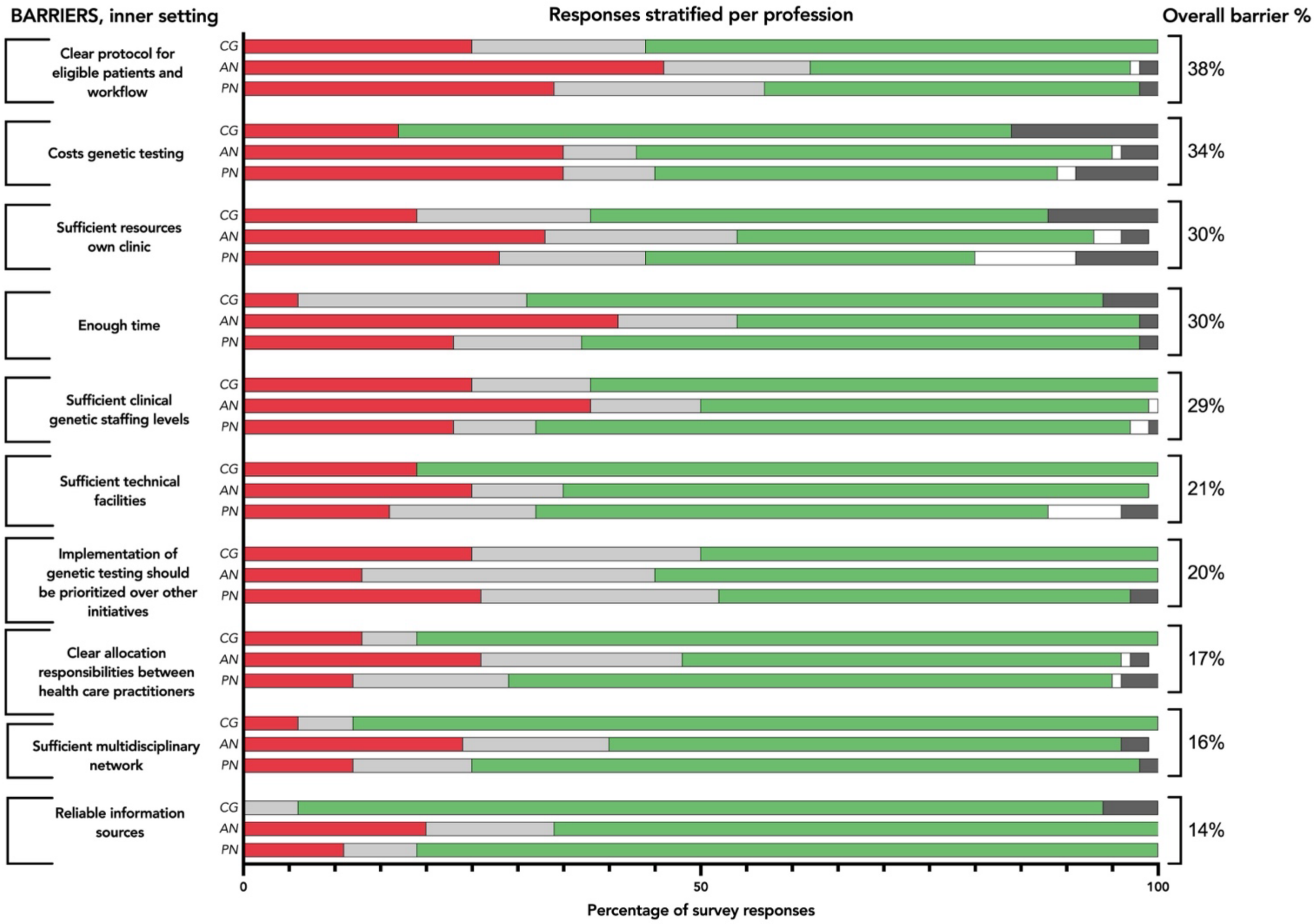

Supplementary Figure S9B. Responses to the 'inner setting' barrier statements regarding the implementation of genetic testing for suspected hereditary kidney disease, stratified for different professions. Pediatric nephrologists (pn), adult nephrologists (an) and clinical geneticists (cg). Responses are categorized as red (disagreed, indicating a barrier), light grey (neutral), green (agreed, indicating no barrier, white (I don't know) and dark grey (NA).

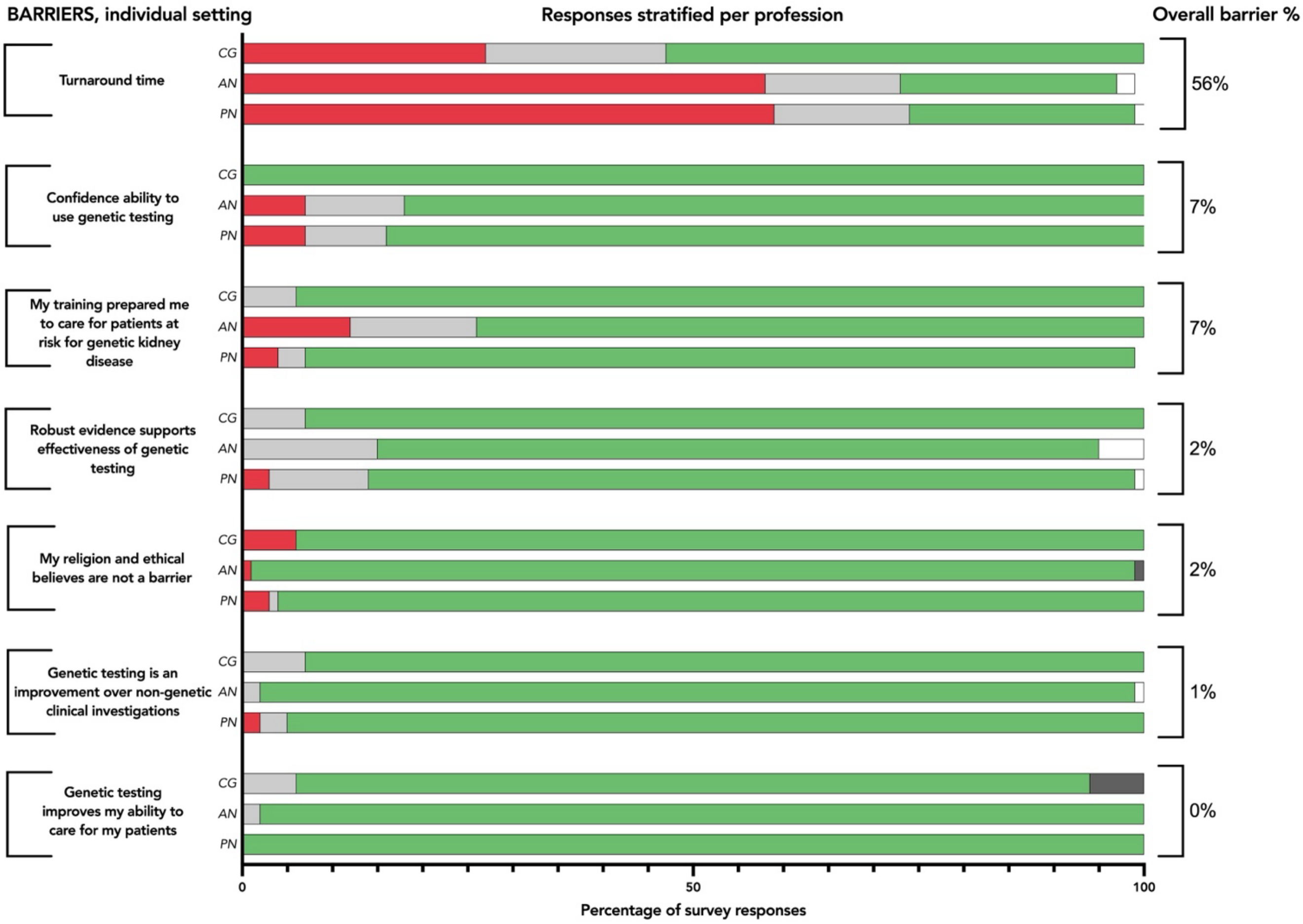

Supplementary Figure S9C. Responses to the 'individual setting' barrier statements regarding the implementation of genetic testing for suspected hereditary kidney disease, stratified for different professions. Pediatric nephrologists (pn), adult nephrologists (an) and clinical geneticists (cg). Responses are categorized as red (disagreed, indicating a barrier), light grey (neutral), green (agreed, indicating no barrier, white (I don't know) and dark grey (NA).

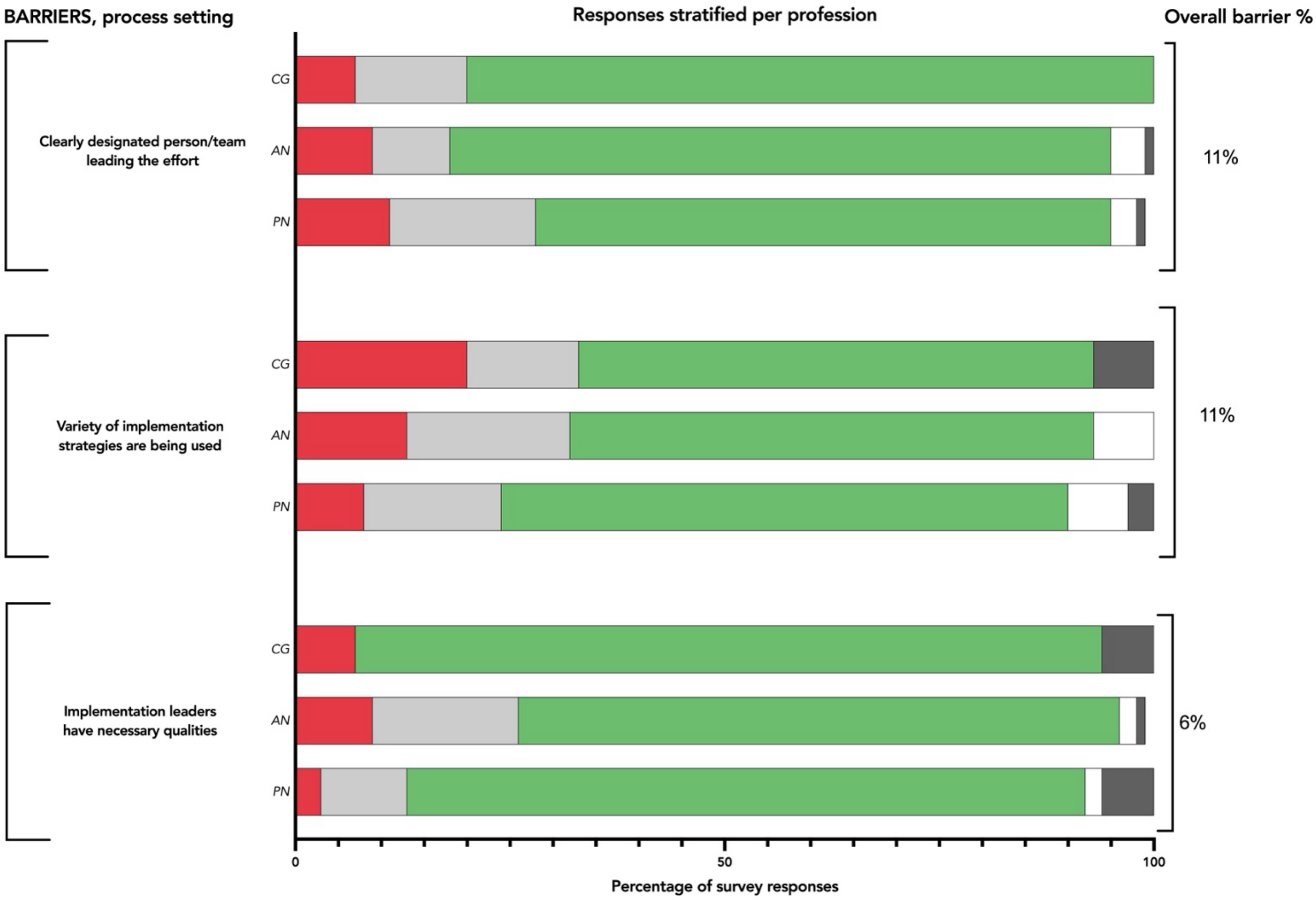

Supplementary Figure S9D. Responses to the 'process setting' barrier statements regarding the implementation of genetic testing for suspected hereditary kidney disease, stratified for different professions. Pediatric nephrologists (pn), adult nephrologists (an) and clinical geneticists (cg). Responses are categorized as red (disagreed, indicating a barrier), light grey (neutral), green (agreed, indicating no barrier, white (I don't know) and dark grey (NA).

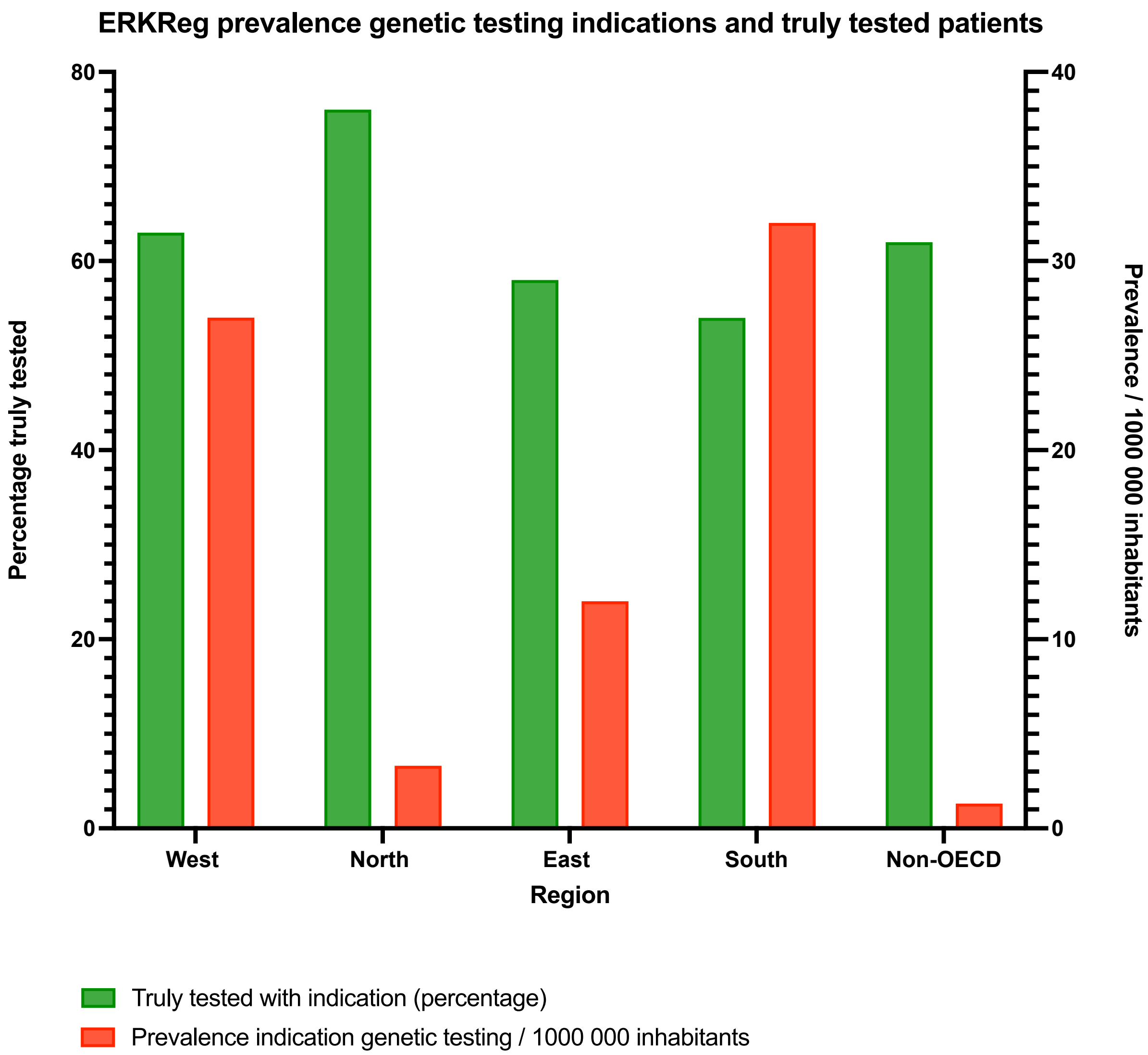

Supplementary Figure S10. ERKReg prevalence genetic testing indications per 1000 000 inhabitants per European region and percentage truly tested patients with an indication for genetic testing.

Supplementary Table S1. European Rare Kidney disease Registry (ERKReg) data, list of included diagnoses and genetic testing indications yes/no.

AA amyloidosis	No strict genetic testing indication
Acquired thrombotic thrombocytopenic purpura (TTP)	No strict genetic testing indication
AL amyloidosis	No strict genetic testing indication
Anti-glomerular basement membrane disease	No strict genetic testing indication
Atypical hemolytic uremic syndrome (aHUS) with anti-factor H antibodies	No strict genetic testing indication
Atypical hemolytic uremic syndrome (aHUS), not further specified	Genetic testing indication
Autoimmune hepatitis	No strict genetic testing indication
C3 glomerulopathy secondary to MGRS	No strict genetic testing indication
Childhood-onset systemic lupus erythematosus (SLE)	No strict genetic testing indication
Childhood-onset systemic lupus erythematosus associated TMA (SLE)	No strict genetic testing indication
Congenital membranous nephropathy due to maternal anti-neutral endopeptidase alloimmunization	No strict genetic testing indication
CREST syndrome	No strict genetic testing indication
Cryoglobulinemic vasculitis	No strict genetic testing indication
De novo thrombotic microangiopathy after kidney transplantation	No strict genetic testing indication
Dense deposit disease	No strict genetic testing indication
Diffuse cutaneous systemic sclerosis	No strict genetic testing indication
Drug-related renal tubular dysgenesis	No strict genetic testing indication
Eosinophilic granulomatosis with polyangiitis	No strict genetic testing indication
Fanconi syndrome, Cisplatin induced	No strict genetic testing indication
Fanconi syndrome, induced by other drug	No strict genetic testing indication
Fanconi syndrome, lead induced	No strict genetic testing indication
Fanconi syndrome, monoclonal Ig light chain-associated	No strict genetic testing indication
Granulomatosis with polyangiitis (Wegener)	No strict genetic testing indication
Heavy chain deposition disease	No strict genetic testing indication
Hypercalciuria, idiopathic	Genetic testing indication
Hypomagnesaemia, drug-induced	No strict genetic testing indication
Idiopathic multidrug-resistant nephrotic syndrome	No strict genetic testing indication
Idiopathic non-lupus full-house nephropathy	No strict genetic testing indication
Idiopathic steroid-resistant nephrotic syndrome with sensitivity to second-line immunouppressive therapy	No strict genetic testing indication
Idiopathic steroid-sensitive nephrotic syndrome	No strict genetic testing indication
Idiopathic steroid-sensitive nephrotic syndrome with secondary steroid resistance	No strict genetic testing indication
IgA nephropathy	No strict genetic testing indication
IgA vasculitis (Henoch Schonlein nephritis, PSH)	No strict genetic testing indication
IgG4-related kidney disease	No strict genetic testing indication
Immunoglobulin-mediated membranoproliferative glomerulonephritis (MPGN)	No strict genetic testing indication
Immunotactoid glomerulopathy	No strict genetic testing indication
Juvenile dermatomyositis	No strict genetic testing indication
Light and heavy chain deposition disease	No strict genetic testing indication
Light chain deposition disease	No strict genetic testing indication
Limited cutaneous systemic sclerosis	No strict genetic testing indication
Limited systemic sclerosis	No strict genetic testing indication
Membranous glomerulonephritis	No strict genetic testing indication
Microscopic polyangiitis	No strict genetic testing indication
Mixed connective tissue disease	No strict genetic testing indication
NA	No strict genetic testing indication
Nephrotic syndrome, syndromic, not otherwise specified	Genetic testing indication
Non-amyloid fibrillary glomerulopathy	No strict genetic testing indication
Non-amyloid monoclonal immunoglobulin deposition disease	No strict genetic testing indication
Other autoimmune dRTA	No strict genetic testing indication
Pauci-immune glomerulonephritis with ANCA	No strict genetic testing indication

Pauci-immune glomerulonephritis without ANCA	No strict genetic testing indication
Polyarteritis nodosa	No strict genetic testing indication
Primary biliary cholangitis	No strict genetic testing indication
Primary localized amyloidosis	No strict genetic testing indication
Primary systemic amyloidosis	No strict genetic testing indication
Proliferative glomerulonephritis with monoclonal immune deposits (PGNMID)	No strict genetic testing indication
Renal artery stenosis, congenital	No strict genetic testing indication
Renal tubular dysgenesis due to twin-twin transfusion	No strict genetic testing indication
Sarcoidosis	No strict genetic testing indication
Shiga toxin-associated hemolytic uremic syndrome (STEC-HUS)	No strict genetic testing indication
Sjogren Syndrome	No strict genetic testing indication
Streptoccous pneumoniae-associated hemolytic uremic syndrome (HUS)	No strict genetic testing indication
Systemic Lupus Erythematosus (SLE)	No strict genetic testing indication
Systemic Lupus Erythematosus associated TMA (SLE)	No strict genetic testing indication
Takayasu arteritis	No strict genetic testing indication
Tubulointerstitial nephritis and uveitis syndrome	No strict genetic testing indication
AApoAI amyloidosis	Genetic testing indication
Adenine phosphoribosyltransferase deficiency	Genetic testing indication
AFib amyloidosis	Genetic testing indication
Alagille syndrome	Genetic testing indication
Alport syndrome, autosomal dominant	Genetic testing indication
Alport syndrome, autosomal recessive	Genetic testing indication
Alport syndrome, digenic	Genetic testing indication
Alport syndrome, X-linked	Genetic testing indication
Alport syndrome, X-linked with diffuse leiomyomatosis	Genetic testing indication
Alstr√∂m Syndrome	Genetic testing indication
ALys amyloidosis	Genetic testing indication
Amelogenesis imperfecta-nephrocalcinosis syndrome	Genetic testing indication
Anterior urethral valve	No strict genetic testing indication
Apparent mineralocorticoid excess	Genetic testing indication
Arthrogryposis-renal dysfunction-cholestasis (ARC) syndrome	Genetic testing indication
Atresia of urethra	No strict genetic testing indication
Atypical hemolytic uremic syndrome (aHUS) due to Methylcobalamin deficiency, type CblC	Genetic testing indication
Atypical hemolytic uremic syndrome (aHUS) with complement gene abnormality	Genetic testing indication
Atypical hemolytic uremic syndrome (aHUS) with DGKE deficiency	Genetic testing indication
Autoimmune interstitial lung disease-arthritis syndrome	Genetic testing indication
Autosomal dominant polycystic kidney disease (ADPKD)	Genetic testing indication
Autosomal dominant distal renal tubular acidosis (dRTA)	Genetic testing indication
Autosomal dominant hypocalcemia	Genetic testing indication
Autosomal dominant hypophosphatemic rickets	Genetic testing indication
Autosomal dominant intermediate Charcot-Marie-Tooth disease type E	Genetic testing indication
Autosomal dominant polycystic kidney disease type 1 with tuberous sclerosis (ADPKD-TSC)	Genetic testing indication
Autosomal dominant primary hypomagnesemia with hypocalciuria	Genetic testing indication
Autosomal dominant proximal renal tubular acidosis	Genetic testing indication
Autosomal recessive distal renal tubular acidosis (dRTA)	Genetic testing indication
Autosomal recessive hypophosphatemic rickets	Genetic testing indication
Autosomal recessive infantile hypercalcemia	Genetic testing indication
Autosomal recessive polycystic kidney disease (ARPKD)	Genetic testing indication
Autosomal recessive proximal renal tubular acidosis	Genetic testing indication
Bardet-Biedl syndrome	Genetic testing indication
Bardet-Biedl syndrome (renal / urinary tract malformation)	Genetic testing indication
Bartter syndrome	Genetic testing indication

Beckwith-Wiedemann syndrome	Genetic testing indication
Bilateral multicystic dysplastic kidney	Genetic testing indication
Bladder exstrophy	No strict genetic testing indication
BOR (branchio-oto-renal) syndrome	Genetic testing indication
Brachydactyly-arterial hypertension syndrome	Genetic testing indication
C3 glomerulonephritis	Genetic testing indication
Cat-eye syndrome	Genetic testing indication
Caudal regression sequence	No strict genetic testing indication
Cloacal exstrophy	No strict genetic testing indication
Collagenopathy, not further specified	Genetic testing indication
Congenital hydronephrosis	No strict genetic testing indication
Congenital nephrotic syndrome-interstitial lung disease-epidermolysis bullosa syndrome	Genetic testing indication
Congenital nephrotic syndrome, Finnish type	Genetic testing indication
Congenital nephrotic syndrome, no genetic cause specified	Genetic testing indication
Congenital primary megaureter, nonrefluxing and unobstructed form	No strict genetic testing indication
Congenital primary megaureter, obstructed form	No strict genetic testing indication
Congenital primary megaureter, refluxing and obstructed	No strict genetic testing indication
Congenital primary megaureter, refluxing form	No strict genetic testing indication
Congenital thrombotic thrombocytopenic purpura (TTP)	Genetic testing indication
Cranioectodermal dysplasia (Sensenbrenner syndrome)	Genetic testing indication
Cystinuria	Genetic testing indication
Dent disease type 1 (CLCN5-related)	Genetic testing indication
Dent disease type 2 (OCRL-related)	Genetic testing indication
Denys-Drash syndrome	Genetic testing indication
Di George syndrome (22q11.2 deletion)	Genetic testing indication
Distal renal tubular acidosis with hemolytic anemia	Genetic testing indication
Donnai-Barrow syndrome	Genetic testing indication
EAST syndrome	Genetic testing indication
Ellis van Creveld syndrome	Genetic testing indication
Epispadias	No strict genetic testing indication
Fabry disease	Genetic testing indication
Familial hyperaldosteronism type I (glucocorticoid-sensitive hypertension)	Genetic testing indication
Familial hyperaldosteronism type III	Genetic testing indication
Familial Mediterranean fever	Genetic testing indication
Familial primary hypomagnesemia with hypercalciuria and nephrocalcinosis with severe ocular involvement	Genetic testing indication
Familial primary hypomagnesemia with hypercalciuria and nephrocalcinosis without severe ocular involvement	Genetic testing indication
Familial primary hypomagnesemia with normocalciuria and normocalcemia	Genetic testing indication
Familial renal glucosuria	Genetic testing indication
Familial steroid-resistant nephrotic syndrome with adrenal insufficiency	Genetic testing indication
Fanconi syndrome, primary	Genetic testing indication
Fibronectin glomerulopathy	Genetic testing indication
Fraser syndrome	Genetic testing indication
Frasier syndrome	Genetic testing indication
Galactosemia	Genetic testing indication
Galloway-Mowat syndrome	Genetic testing indication
Generalized pseudohypoaldosteronism type 1	Genetic testing indication
Genetic steroid-resistant nephrotic syndrome (SRNS)	Genetic testing indication
Gitelman syndrome	Genetic testing indication
Gitelman-like syndrome	Genetic testing indication
Glycogen storage disease due to glucose-6-phosphatase deficiency	Genetic testing indication
Glycogen storage disease due to GLUT2 deficiency	Genetic testing indication
HANAC syndrome	Genetic testing indication
HDR (Hypoparathyroidism-deafness-renal disease) syndrome	Genetic testing indication

Hereditary amyloidosis with primary renal involement	Genetic testing indication
Hereditary hypophosphatemic rickets with hypercalciuria	Genetic testing indication
Hereditary renal hypouricemia	Genetic testing indication
HNF1B-related autosomal dominant tubulointerstitial kidney disease (ADTKD)	Genetic testing indication
Hypocomplemententemic urticarial vasculitis	Genetic testing indication
Hypokalemic alkalosis, CLDN10 associated	Genetic testing indication
Hypophosphatemia, dominant, with nephrolithiasis or osteoporosis	Genetic testing indication
Hypotonia-cystinuria syndrome	Genetic testing indication
Hypoxanthine-guanine phosphoribosyltransferase deficiency	Genetic testing indication
Imerslund-Gr√§sbeck syndrome	Genetic testing indication
Infantile nephronophthisis	Genetic testing indication
Infantile nephropathic cystinosis	Genetic testing indication
Jeune syndrome (ciliopathies)	Genetic testing indication
Jeune syndrome (renal / urinary tract malformation)	Genetic testing indication
Joubert syndrome with oculorenal defect	Genetic testing indication
Joubert syndrome with renal defect	Genetic testing indication
Juvenile nephronophthisis	Genetic testing indication
Juvenile nephropathic cystinosis	Genetic testing indication
Kabuki Syndrome	Genetic testing indication
Kallmann syndrome	Genetic testing indication
Karyomegalic interstitial nephritis	Genetic testing indication
Late-onset nephronophthisis	Genetic testing indication
Leigh syndrome with nephrotic syndrome	Genetic testing indication
Lethal fetal cerebrorenogenitourinary agenesis/hypoplasia syndrome	Genetic testing indication
Liddle Syndrome	Genetic testing indication
Lipoprotein glomerulopathy	Genetic testing indication
Lowe syndrome	Genetic testing indication
Lysinuric protein intolerance	Genetic testing indication
Mayer-Rokitansky-K	Genetic testing indication
Meckel syndrome (ciliopathies)	Genetic testing indication
Medullary sponge kidney	Genetic testing indication
Megacystis-megaureter syndrome	Genetic testing indication
Methylmalonic acidemia, Vitamin B12-responsive	Genetic testing indication
Methylmalonic acidemia, Vitamin B12-unresponsive	Genetic testing indication
Microscopic (including familial) hematuria	Genetic testing indication
MUC1-related autosomal dominant tubulointerstitial kidney disease (ADTKD)	Genetic testing indication
Multicentric carpo-tarsal osteolysis with or without nephropathy	Genetic testing indication
MYH9-related disease	Genetic testing indication
Nail-patella syndrome	Genetic testing indication
Nephrogenic diabetes insipidus	Genetic testing indication
Nephrogenic syndrome of inappropriate antidiuresis	Genetic testing indication
Nephrotic syndrome-deafness-pretibial epidermolysis bullosa syndrome	Genetic testing indication
Nephrotic syndrome, familial, steroid-resistant (SRNS), with sensorineural deafness	Genetic testing indication
Neurofibromatosis type 1	Genetic testing indication
Neurogenic bladder, congenital or acquired	No strict genetic testing indication
Noonan syndrome	Genetic testing indication
Ochoa syndrome	Genetic testing indication
OFD (Orofaciodigital) syndrome type 1	Genetic testing indication
Oligomeganephronia	No strict genetic testing indication
Osteopetrosis with renal tubular acidosis	Genetic testing indication
Pallister-Hall syndrome	Genetic testing indication
Pierson syndrome	Genetic testing indication
Posterior urethral valve (PUV)	No strict genetic testing indication

Primary hyperoxaluria type 1 (PH1)	Genetic testing indication
Primary hyperoxaluria type 2 (PH2)	Genetic testing indication
Primary hyperoxaluria type 3 (PH3)	Genetic testing indication
Primary hypomagnesemia with secondary hypocalcemia	Genetic testing indication
Prune belly syndrome	Genetic testing indication
Pseudohypoaldosteronism type 2	Genetic testing indication
RCAD (Renal cysts and diabetes) syndrome	Genetic testing indication
REN-related autosomal dominant tubulointerstitial kidney disease (ADTKD, FJHN type 1)	Genetic testing indication
Renal agenesis, bilateral	No strict genetic testing indication
Renal agenesis, unilateral	No strict genetic testing indication
Renal coloboma syndrome	Genetic testing indication
Renal dysplasia, bilateral	No strict genetic testing indication
Renal dysplasia, unilateral	No strict genetic testing indication
Renal hypoplasia, bilateral	No strict genetic testing indication
Renal hypoplasia, unilateral	No strict genetic testing indication
Renal nutcracker syndrome	Genetic testing indication
Renal pseudohypoaldosteronism type 1	Genetic testing indication
Renal tubular dysgenesis of genetic origin	Genetic testing indication
Renal-hepatic-pancreatic dysplasia	Genetic testing indication
Rubinstein-Taybi syndrome	Genetic testing indication
Saldino-Mainzer (conorenal) syndrome	Genetic testing indication
Schimke immuno-osseous dysplasia	Genetic testing indication
Senior-Boichis syndrome	Genetic testing indication
Senior-Loken syndrome	Genetic testing indication
Severe oculo-renal-cerebellar syndrome	Genetic testing indication
Simpson-Golabi-Behmel syndrome	Genetic testing indication
Smith-Lemli-Opitz syndrome	Genetic testing indication
Townes-Brocks syndrome	Genetic testing indication
Tuberous sclerosis complex (TSC)	Genetic testing indication
Tubulopathy due to mitochondrial oxidative phosphorylation disorder	Genetic testing indication
Turner syndrome	Genetic testing indication
UMOD-related autosomal dominant tubulointerstitial kidney disease (ADTKD)	Genetic testing indication
Unilateral multicystic dysplastic kidney	No strict genetic testing indication
Ureteropelvic junction (UPJ) obstruction (bilateral or in solitary kidney)	No strict genetic testing indication
VACTERL/VATER association	Genetic testing indication
Vesicoureteric reflux (VUR), familial	No strict genetic testing indication
Vesicoureteric reflux (VUR), high-grade	No strict genetic testing indication
Von Hippel-Lindau disease (vHL)	Genetic testing indication
Williams syndrome	Genetic testing indication
X-linked hypophosphatemic rickets (XLHR)	Genetic testing indication
Xanthinuria type II	Genetic testing indication

Supplementary Table S2. Overview countries of origin survey participants, including stratification into European regions.

Region, n survey responses per region	Country, n survey response
**West, n = 92**	Austria, n = 4
	Belgium, n = 11
	France, n = 26
	Germany, n = 23
	Republic of Ireland, n = 2
	Switzerland, n = 2
	The Netherlands, n = 18
	United Kingdom, n = 6
**East, n = 31**	Czech Republic, n = 3
	Estonia, n = 5
	Hungary, n = 1
	Latvia, n = 1
	Lithuania, n = 3
	Poland, n = 14
	Slovakia, n = 1
	Slovenia, n = 3
**South, n = 58**	Greece, n = 8
	Italy, n = 23
	Portugal, n = 6
	Spain, n = 21
**North, n = 14**	Denmark, n = 5
	Finland, n = 2
	Norway, n = 3
	Sweden, n = 4
**Non-OECD, n = 45**	Albania, n = 3
	Armenia, n = 1
	Belarus, n = 2
	Bulgaria, n = 3
	Croatia, n = 4
	Cyprus, n = 1
	Kazakhstan, n = 2
	Malta, n = 3
	Montenegro, n = 1
	North-Macedonia, n = 1
	Romania, n = 4
	Russia, n = 2
	Serbia, n = 4
	Turkey, n = 12
	Ukraine, n = 2

Table S3. Logistic aspects, for total survey sample and stratified per European region.

Variable	Total cohort<br>N = 240	West<br>N = 92	East<br>N = 31	South<br>N = 58	North<br>N = 14	Non-OECD<br>N = 45
**Performance pretest counseling, in your patients**						
• Pediatric nephrologist	127/240 (53%)	46 (50%)	17 (55%)	24 (41%)	10 (71%)	30 (67%)
• Adult nephrologist	94/240 (39%)	44 (48%)	9 (29%)	27 (47%)	3 (21%)	11 (24%)
• Clinical geneticist	115/240 (48%)	38 (41%)	20 (65%)	31 (53%)	8 (57%)	18 (40%)
• Other	5/240 (2%)	3 (3%)	0	2 (3%)	0	0
**Location diagnostic testing**						
• Laboratory, own hospital	151/239 (63%)	58/91 (64%)	23 (74%)	39 (67%)	11 (79%)	20 (44%)
• Laboratory, other hospital own country	118/239 (49%)	59/91 (65%)	15 (48%)	27 (47%)	8 (57%)	9 (20%)
• Commercial laboratory own country	63/239 (26%)	25/91 (28%)	9 (22%)	13 (22%)	2 (14%)	14 (31%)
• Laboratory, other hospital abroad	28/239 (12%)	2/91 (2%)	9 (29%)	6 (10%)	0	11 (24%)
• Commercial laboratory abroad	31/239 (13%)	2/91 (2%)	8 (26%)	1 (2%)	2 (14%)	18 (40%)
• Only available for research	12/239 (5%)	1/91 (1%)	2 (7%)	2 (3%)	1 (7%)	6 (13%)
• Other	2/239 (1%)	0	2 (7%)	0	0	0
**Performance interpretation sequencing data and results generating**						
• Technician/bio-informatician	64/234 (27%)	28/90 (31%)	5/29 (17%)	21/56 (38%)	4/14 (29%)	6/45 (13%)
• Lab specialist	112/234 (48%)	48/90 (53%)	13/29 (45%)	31/56 (55%)	4/14 (29%)	16/45 (36%)
• Clinical geneticist	184/234 (79%)	74/90 (82%)	24/29 (83%)	42/56 (75%)	14/14 (100%)	30/45 (67%)
• Researcher	36/234 (16%)	16/90 (18%)	3/29 (10%)	7/56 (13%)	0	10/45 (22%)
• Nephrologist	32/234 (14%)	14/90 (16%)	1/29 (3%)	10/56 (18%)	1/14 (7%)	6/45 (13%)
• Pediatric nephrologist	49/234 (21%)	16/90 (18%)	4/29 (14%)	11/56 (20%)	2/14 (14%)	16/45 (36%)
• Don't know	5/234 (2%)	3/90 (3%)	0	0	0	2/45 (4%)
• Other	4/234 (2%)	2/90 (2%)	1/29 (3%)	1/56 (2%)	0	0
**Performance post-test counseling, in your patients**						
• Pediatric nephrologist	136/233 (58%)	51/89 (57%)	17/29 (59%)	28/56 (50%)	10/14 (71%)	30/45 (67%)
• Adult nephrologist	91/233 (39%)	44/89 (49%)	5/29 (17%)	27/56 (48%)	3/14 (21%)	12/45 (27%)
• Clinical geneticist	156/233 (67%)	59/89 (66%)	26/29 (90%)	36/56 (64%)	11/14 (79%)	24/45 (53%)
• Other	4/233 (2%)	2/89 (2%)	0	1/56 (2%)	0	1/45 (2%)
**Difference funding in types of genetic tests**						
• Yes	48/233 (21%)	18/89 (20%)	12/29 (41%)	6/56 (11%)	0	12/45 (27%)
• No	133/233 (57%)	49/89 (55%)	13/29 (45%)	38/56 (68%)	12/14 (86%)	21/45 (47%)
• Don't know	52/233 (22%)	22/89 (25%)	4/29 (14%)	12/56 (21%)	2/14 (14%)	12/45 (27%)

**Which party usually pays for genetic testing**						
• Patient/family	38/232 (16%)	5/89 (6%)	3/28 (11%)	11/56 (20%)	0	19/45 (42%)
• Patient's health insurance	70/232 (30%)	50/89 (56%)	7/28 (25%)	3/56 (5%)	0	10/45 (22%)
• Government/public health service	147/232 (63%)	41/89 (46%)	24/28 (86%)	47/56 (84%)	12/14 (86%)	21/45 (47%)
• Hospital/department (local budget)	54/232 (23%)	26/89 (29%)	11/28 (39%)	7/56 (13%)	5/14 (36%)	5/45 (11%)
• Commercial company for free	12/232 (5%)	3/89 (3%)	2/28 (7%)	4/56 (7%)	0	3/45 (7%)
• Research funded	45/232 (19%)	15/89 (17%)	8/28 (29%)	10/56 (18%)	1/14 (7%)	11/45 (24%)
• I don't know	3/232 (1%)	3/89 (3%)	0	0	0	0
• Other	3/232 (1%)	0	0	0	0	3/45 (7%)
**How often do patients struggle with costs of genetic testing**						
• (Almost) always	10/232 (4%)	0	0	3/56 (5%)	0	7/45 (16%)
• Often	11/232 (5%)	1/89 (1%)	1/28 (4%)	2/56 (4%)	0	7/45 (16%)
• Fifty-fifty	10/232 (4%)	0	0	2/56 (4%)	0	8/45 (18%)
• Sometimes	39/232 (17%)	19/89 (21%)	9/28 (32%)	4/56 (7%)	0	7/45 (16%)
• (Almost) never	162/232 (70%)	69/89 (78%)	18/28 (64%)	45/56 (80%)	14/14 (100%)	16/45 (36%)
**Received survey via**						
• ERA	47/231 (20%)	13/89 (15%)	7/28 (25%)	14/56 (25%)	4/14 (29%)	9/44 (21%)
• ESPN	93/231 (40%)	26/89 (29%)	16/28 (57%)	15/56 (27%)	7/14 (50%)	29/44 (66%)
• ERKNet	115/231 (50%)	51/89 (57%)	15/28 (54%)	38/56 (68%)	3/14 (21%)	8/44 (18%)
• ERN Ithaca	5/231 (2%)	1/89 (1%)	0	1/56 (2%)	1/14 (7%)	2/44 (5%)
• Other	15/231 (7%)	9/89 (10%)	0	4/56 (7%)	0	2/44 (5%)

Supplementary Table S4. Barrier statements and responses from all survey participants.

Barriers to implementation of genetic testing in daily practice	Strongly disagree/disagree	Neutral	Agree/strongly agree	Don't know	NA
Costs of genetic testing are a barrier*	111/231 (48%)	21/231 (9%)	78/231 (34%)	3/231 (1%)	18/231 (8%)
**Outer setting**					
Insurance for individuals with genetic diagnosis may prevent further implementation*	61/221 (28%)	31/221 (14%)	61/221 (28%)	33/221 (15%)	35/221 (16%)
Legislation and regulations allow further implementation	24/220 (11%)	34/220 (16%)	128/220 (58%)	25/220 (11%)	9/220 (4%)
There are clear professional group guidelines and recommendations for genetic testing in kidney disease in my country	77/221 (35%)	38/221 (17%)	93/221 (42%)	8/221 (4%)	5/221 (2%)
**Inner setting**					
I have enough time to facilitate genetic testing	65/220 (30%)	31/220 (14%)	118/220 (54%)	0/220 (0%)	6/220 (3%)
I can find/use reliable sources of information to apply for genetic testing	31/220 (14%)	19/220 (9%)	168/220 (76%)	0/220 (0%)	2/220 (1%)
Sufficient clinical genetic staffing levels for patients with suspected hereditary kidney disease	63/219 (29%)	23/219 (11%)	127/219 (58%)	3/219 (1%)	3/219 (1%)
Clear protocol for eligible patients and workflow to obtain genetic testing	84/219 (38%)	43/219 (20%)	85/219 (39%)	1/219 (0.5%)	6/219 (3%)
Sufficient multidisciplinary network to facilitate genetic testing in patients with suspected hereditary kidney disease	36/219 (16%)	30/219 (14%)	146/219 (67%)	0/219 (0%)	7/219 (3%)
Clear allocation of responsibilities between health care practitioners	38/219 (17%)	40/219 (18%)	130/219 (59%)	2/219 (1%)	9/219 (4%)
Further implementation of genetic testing should be prioritized over other initiatives to improve care for CKD patients	43/219 (20%)	65/219 (30%)	107/219 (49%)	0/219 (0%)	4/219 (2%)
Technical facilities are sufficient	45/219 (21%)	28/219 (13%)	130/219 (59%)	9/219 (4%)	7/219 (3%)
My clinic's financial resources are sufficient to facilitate further implementation of genetic testing	65/219 (30%)	38/219 (17%)	84/219 (38%)	15/219 (7%)	17/219 (8%)
**Individual characteristics**					
I am confident in my ability to use the results of genetic testing	14/217 (7%)	21/217 (10%)	181/217 (83%)	0/217 (0%)	1/217 (0.5%)
My training has prepared me to look after patients at high risk for hereditary kidney disease	16/217 (7%)	16/217 (7%)	184/217 (85%)	0/217 (0%)	1/217 (0.5%)
My religion and ethical believes are not a barrier for further implementation of genetic testing	4/217 (2%)	1/217 (0.5%)	210/217 (97%)	0/217 (0%)	2/217 (1%)
Genetic testing improves my ability to care for my patients	0/217 (0%)	3/217 (1%)	211/217 (97%)	0/217 (0%)	3/217 (1%)
I only request genetic diagnostics when there are clinical implications involved	68/217 (31%)	23/217 (11%)	122/217 (56%)	0/217 (0%)	4/217 (2%)
There is robust evidence to support the effectiveness of genetic testing	4/216 (2%)	26/216 (12%)	181/216 (84%)	5/216 (2%)	0/216 (0%)
Genetic testing is an improvement over non-genetic clinical investigations for suspected hereditary kidney disease	2/216 (1%)	6/216 (3%)	206/216 (95%)	1/216 (0.5%)	1/216 (0.5%)
Turnaround time is a barrier for further implementation of genetic testing in the clinical workflow*, **	57/216 (26%)	35/216 (16%)	121/216 (56%)	3/216 (1%)	0/216 (0%)
**Process**					
Clearly designated person or team leads the effort to continue further implementation of genetic testing into clinical practice	23/214 (11%)	31/214 (15%)	151/214 (71%)	7/214 (3%)	2/214 (1%)
Variety of strategies are being used to enable staff to continue further implementation of genetic testing	23/214 (11%)	38/214 (18%)	134/214 (63%)	14/214 (7%)	5/214 (2%)
Implementation leaders have the necessary qualities and skills to successfully incorporate genetic testing into clinical practice	12/214 (6%)	28/214 (13%)	161/214 (75%)	4/214 (2%)	9/214 (4%)

The order of barriers was adjusted for the figures to facilitate interpretation, ranked from the most to the least frequently reported barrier.

*=turned around agree/disagree options in the graphs to improve visual interpretation of barriers (red bars).

Supplementary Table S5. Barrier statements and responses from Western countries.

Barriers to implementation of genetic testing in daily practice	Strongly disagree/disagree	Neutral	Agree/strongly agree	Don't know	NA
Costs of genetic testing*	52/88 (59%)	9/88 (10%)	16/88 (18%)	2/88 (2%)	9/88 (10%)
**Outer setting**					
Insurance for individuals with genetic diagnosis may prevent further implementation*	29/87 (33%)	12/87 (14%)	31/87 (36%)	9/87 (10%)	6/87 (7%)
Legislation and regulations allow further implementation	5/86 (6%)	13/86 (15%)	54/86 (63%)	9/86 (11%)	5/86 (6%)
There are clear professional group guidelines and recommendations for genetic testing in kidney disease in my country	21/86 (24%)	12/86 (14%)	50/86 (58%)	3/86 (4%)	0
**Inner setting**					
I have enough time to facilitate genetic testing	19/86 (22%)	11/86 (13%)	54/86 (63%)	0	2/86 (2%)
I can find/use reliable sources of information to apply for genetic testing	7/86 (8%)	7/86 (8%)	70/86 (81%)	0	2/86 (2%)
Sufficient clinical genetic staffing levels for patients with suspected hereditary kidney disease	18/86 (21%)	5/86 (6%)	61/86 (71%)	1/86 (1%)	1/86 (1%)
Clear protocol for eligible patients and workflow to obtain genetic testing	30/86 (35%)	17/86 (20%)	38/86 (44%)	0	1/86 (1%)
Sufficient multidisciplinary network to facilitate genetic testing in patients with suspected hereditary kidney disease	7/86 (8%)	6/86 (7%)	71/86 (83%)	0	2/86 (2%)
Clear allocation of responsibilities between health care practitioners	10/86 (12%)	15/86 (17%)	57/86 (66%)	1/86 (1%)	3/86 (4%)
Further implementation of genetic testing should be prioritized over other initiatives to improve care for CKD patients	21/86 (24%)	31/86 (36%)	32/86 (37%)	0	2/86 (2%)
Technical facilities are sufficient	15/86 (17%)	11/86 (13%)	56/86 (65%)	1/86 (1%)	3/86 (4%)
My clinic's financial resources are sufficient to facilitate further implementation of genetic testing	21/86 (24%)	14/86 (16%)	35/86 (41%)	8/86 (9%)	8/86 (9%)
**Individual characteristics**					
I am confident in my ability to use the results of genetic testing	1/85 (1%)	5/85 (6%)	78/85 (92%)	0	1/85 (1%)
My training has prepared me to look after patients at high risk for hereditary kidney disease	5/85 (6%)	5/85 (6%)	74/85 (87%)	0	1/85 (1%)
My religion and ethical believes are not a barrier for further implementation of genetic testing	1/85 (1%)	0	83/85 (98%)	0	1/85 (1%)
Genetic testing improves my ability to care for my patients	0	0	83/85 (98%)	0	2/85 (2%)
I only request genetic diagnostics when there are clinical implications involved	30/85 (35%)	11/85 (13%)	42/85 (49%)	0	2/85 (2%)
There is robust evidence to support the effectiveness of genetic testing	1/85 (1%)	8/85 (9%)	74/85 (87%)	2/85 (2%)	0
Genetic testing is an improvement over non-genetic clinical investigations for suspected hereditary kidney disease	0	2/85 (2%)	83/85 (98%)	0	0
Turnaround time is a barrier for further implementation of genetic testing in the clinical workflow*, **	31/85 (37%)	6/85 (7%)	48/85 (57%)	0	0
**Process**					
Clearly designated person or team leads the effort to continue further implementation of genetic testing into clinical practice	9/85 (11%)	7/85 (7%)	67/85 (79%)	2/85 (2%)	1/85 (1%)
Variety of strategies are being used to enable staff to continue further implementation of genetic testing	10/85 (12%)	10/85 (12%)	56/85 (66%)	7/85 (8%)	2/85 (2%)
Implementation leaders have the necessary qualities and skills to successfully incorporate genetic testing into clinical practice	2/85 (2%)	7/85 (8%)	70/85 (82%)	1/85 (1%)	5/85 (6%)

The order of barriers was adjusted for the figures to facilitate interpretation, ranked from the most to the least frequently reported barrier.

*=turned around agree/disagree options in the graphs to improve visual interpretation of barriers (red bars).

Supplementary Table S6. Barrier statements and responses from Eastern countries.

**Barriers to implementation of genetic testing in daily practice**	**Strongly disagree/disagree**	**Neutral**	**Agree/strongly agree**	**Don't know**	**NA**
Costs of genetic testing*	15/28 (54%)	6/28 (21%)	6/28 (21%)	1/28 (4%)	0
**Outer setting**					
Insurance for individuals with genetic diagnosis may prevent further implementation*	9/26 (35%)	5/26 (19%)	7/26 (27%)	4/26 (15%)	1/26 (4%)
Legislation and regulations allow further implementation	3/26 (12%)	3/26 (12%)	16/26 (62%)	3/26 (12%)	1/26 (4%)
There are clear professional group guidelines and recommendations for genetic testing in kidney disease in my country	11/27 (41%)	6/27 (22%)	10/27 (37%)	0	0
**Inner setting**					
I have enough time to facilitate genetic testing	11/26 (42%)	2/26 (8%)	13/26 (50%)	0	0
I can find/use reliable sources of information to apply for genetic testing	3/26 (12%)	3/26 (12%)	20/26 (77%)	0	0
Sufficient clinical genetic staffing levels for patients with suspected hereditary kidney disease	8/26 (31%)	3/26 (12%)	15/26 (58%)	0	0
Clear protocol for eligible patients and workflow to obtain genetic testing	7/26 (27%)	10/26 (39%)	9/26 (35%)	0	0
Sufficient multidisciplinary network to facilitate genetic testing in patients with suspected hereditary kidney disease	4/26 (15%)	5/26 (19%)	17/26 (65%)	0	0
Clear allocation of responsibilities between health care practitioners	6/26 (23%)	4/26 (15%)	16/26 (62%)	0	0
Further implementation of genetic testing should be prioritized over other initiatives to improve care for CKD patients	3/26 (12%)	7/26 (27%)	16/26 (62%)	0	0
Technical facilities are sufficient	3/26 (12%)	5/26 (19%)	14/26 (54%)	4/26 (15%)	0
My clinic's financial resources are sufficient to facilitate further implementation of genetic testing	7/26 (27%)	8/26 (31%)	9/26 (35%)	1/26 (4%)	1/26 (4%)
**Individual characteristics**					
I am confident in my ability to use the results of genetic testing	3/26 (12%)	3/26 (12%)	20/26 (77%)	0	0
My training has prepared me to look after patients at high risk for hereditary kidney disease	4/26 (15%)	3/26 (12%)	19/26 (73%)	0	0
My religion and ethical believes are not a barrier for further implementation of genetic testing	1/26 (4%)	0	25/26 (96%)	0	0
Genetic testing improves my ability to care for my patients	0	0	26/26 (100%)	0	0
I only request genetic diagnostics when there are clinical implications involved	8/26 (31%)	1/26 (4%)	17/26 (65%)	0	0
There is robust evidence to support the effectiveness of genetic testing	0	2/26 (8%)	24/26 (92%)	0	0
Genetic testing is an improvement over non-genetic clinical investigations for suspected hereditary kidney disease	1/26 (4%)	0	24/26 (92%)	0	1/26 (4%)
Turnaround time is a barrier for further implementation of genetic testing in the clinical workflow*, **	3/26 (12%)	5/26 (19%)	17/26 (65%)	1/26 (4%)	0
**Process**					
Clearly designated person or team leads the effort to continue further implementation of genetic testing into clinical practice	4/26 (15%)	1/26 (4%)	21/26 (81%)	0	0
Variety of strategies are being used to enable staff to continue further implementation of genetic testing	2/26 (8%)	6/26 (23%)	16/26 (62%)	2/26 (8%)	0
Implementation leaders have the necessary qualities and skills to successfully incorporate genetic testing into clinical practice	1/26 (4%)	7/26 (27%)	18/26 (69%)	0	0

The order of barriers was adjusted for the figures to facilitate interpretation, ranked from the most to the least frequently reported barrier.

*=turned around agree/disagree options in the graphs to improve visual interpretation of barriers (red bars).

Supplementary Table S7. Barrier statements and responses from Southern countries

**Barriers to implementation of genetic testing in daily practice**	**Strongly disagree/disagree**	**Neutral**	**Agree/strongly agree**	**Don't know**	**NA**
Costs of genetic testing*	25/56 (45%)	2/56 (4%)	21/56 (38%)	0	8/56 (14%)
**Outer setting**					
Insurance for individuals with genetic diagnosis may prevent further implementation*	9/52 (17%)	7/52 (14%)	10/52 (19%)	6/52 (12%)	20/52 (39%)
Legislation and regulations allow further implementation	7/52 (14%)	7/52 (14%)	30/52 (58%)	6/52 (12%)	2/52 (4%)
There are clear professional group guidelines and recommendations for genetic testing in kidney disease in my country	20/52 (39%)	10/52 (19%)	19/52 (37%)	2/52 (4%)	1/52 (2%)
**Inner setting**					
I have enough time to facilitate genetic testing	20/52 (39%)	7/52 (14%)	24/52 (46%)	0	1/52 (2%)
I can find/use reliable sources of information to apply for genetic testing	11/52 (21%)	4/52 (8%)	37/52 (71%)	0	0
Sufficient clinical genetic staffing levels for patients with suspected hereditary kidney disease	20/51 (39%)	7/51 (14%)	24/51 (47%)	0	0
Clear protocol for eligible patients and workflow to obtain genetic testing	18/51 (35%)	9/51 (18%)	23/51 (45%)	0	1/51 (2%)
Sufficient multidisciplinary network to facilitate genetic testing in patients with suspected hereditary kidney disease	11/51 (22%)	8/51 (16%)	32/51 (63%)	0	0
Clear allocation of responsibilities between health care practitioners	10/51 (20%)	10/51 (20%)	31/51 (61%)	0	0
Further implementation of genetic testing should be prioritized over other initiatives to improve care for CKD patients	7/51 (14%)	17/51 (33%)	27/51 (53%)	0	0
Technical facilities are sufficient	12/51 (24%)	5/51 (10%)	32/51 (63%)	2/51 (4%)	0
My clinic's financial resources are sufficient to facilitate further implementation of genetic testing	15/51 (29%)	8/51 (16%)	21/51 (41%)	4/51 (8%)	3/51 (6%)
**Individual characteristics**					
I am confident in my ability to use the results of genetic testing	7/51 (14%)	5/51 (10%)	39/51 (77%)	0	0
My training has prepared me to look after patients at high risk for hereditary kidney disease	4/51 (8%)	4/51 (8%)	43/51 (84%)	0	0
My religion and ethical believes are not a barrier for further implementation of genetic testing	1/51 (2%)	1/51 (2%)	48/51 (94%)	0	1/51 (2%)
Genetic testing improves my ability to care for my patients	0	0	50/51 (98%)	0	1/51 (2%)
I only request genetic diagnostics when there are clinical implications involved	18/51 (35%)	7/51 (14%)	25/51 (49%)	0	1/51 (2%)
There is robust evidence to support the effectiveness of genetic testing	1/51 (2%)	7/51 (14%)	42/51 (82%)	1/51 (2%)	0
Genetic testing is an improvement over non-genetic clinical investigations for suspected hereditary kidney disease	1/51 (2%)	1/51 (2%)	49/51 (96%)	0	0
Turnaround time is a barrier for further implementation of genetic testing in the clinical workflow*, **	5/51 (10%)	13/51 (26%)	32/51 (63%)	1/51 (2%)	0
**Process**					
Clearly designated person or team leads the effort to continue further implementation of genetic testing into clinical practice	5/50 (10%)	8/50 (16%)	35/50 (70%)	2/50 (4%)	0
Variety of strategies are being used to enable staff to continue further implementation of genetic testing	6/50 (12%)	9/50 (18%)	34/50 (68%)	1/50 (2%)	0
Implementation leaders have the necessary qualities and skills to successfully incorporate genetic testing into clinical practice	6/50 (12%)	4/50 (8%)	39/50 (78%)	0	1/50 (2%)

The order of barriers was adjusted for the figures to facilitate interpretation, ranked from the most to the least frequently reported barrier.

*=turned around agree/disagree options in the graphs to improve visual interpretation of barriers (red bars).

Supplementary Table S8. Barrier statements and responses from Northern countries.

Barriers to implementation of genetic testing in daily practice	Strongly disagree/disagree	Neutral	Agree/strongly agree	Don't know	NA
Costs of genetic testing are a barrier*	10/14 (71%)	2/14 (14%)	2/14 (14%)	0	0
**Outer setting**					
Insurance for individuals with genetic diagnosis may prevent further implementation*	6/14 (43%)	1/14 (7%)	1/14 (7%)	2/14 (14%)	4/14 (29%)
Legislation and regulations allow further implementation	2/14 (14%)	3/14 (21%)	8/14 (57%)	1/14 (7%)	0
There are clear professional group guidelines and recommendations for genetic testing in kidney disease in my country	6/14 (43%)	2/14 (14%)	6/14 (43%)	0	0
**Inner setting**					
I have enough time to facilitate genetic testing	4/14 (29%)	2/14 (14%)	8/14 (57%)	0	0
I can find/use reliable sources of information to apply for genetic testing	3/14 (21%)	1/14 (7%)	10/14 (71%)	0	0
Sufficient clinical genetic staffing levels for patients with suspected hereditary kidney disease	1/14 (7%)	0	13/14 (93%)	0	0
Clear protocol for eligible patients and workflow to obtain genetic testing	7/14 (50%)	3/14 (21%)	3/14 (21%)	1/14 (7%)	0
Sufficient multidisciplinary network to facilitate genetic testing in patients with suspected hereditary kidney disease	2/14 (14%)	4/14 (29%)	8/14 (57%)	0	0
Clear allocation of responsibilities between health care practitioners	1/14 (7%)	3/14 (21%)	10/14 (71%)	0	0
Further implementation of genetic testing should be prioritized over other initiatives to improve care for CKD patients	8/14 (57%)	3/14 (21%)	3/14 (21%)	0	0
Technical facilities are sufficient	0	1/14 (7%)	13/14 (93%)	0	0
My clinic's financial resources are sufficient to facilitate further implementation of genetic testing	2/14 (14%)	1/14 (7%)	11/14 (79%)	0	0
**Individual characteristics**					
I am confident in my ability to use the results of genetic testing	0	3/14 (21%)	11/14 (79%)	0	0
My training has prepared me to look after patients at high risk for hereditary kidney disease	0	1/14 (7%)	13/14 (93%)	0	0
My religion and ethical believes are not a barrier for further implementation of genetic testing	0	0	14/14 (100%)	0	0
Genetic testing improves my ability to care for my patients	0	1/14 (7%)	13/14 (93%)	0	0
I only request genetic diagnostics when there are clinical implications involved	2/14 (14%)	2/14 (14%)	10/14 (71%)	0	0
There is robust evidence to support the effectiveness of genetic testing	1/14 (7%)	5/14 (36%)	7/14 (50%)	1/14 (7%)	0
Genetic testing is an improvement over non-genetic clinical investigations for suspected hereditary kidney disease	0	1/14 (7%)	12/14 (86%)	1/14 (7%)	0
Turnaround time is a barrier for further implementation of genetic testing in the clinical workflow*, **	7/14 (50%)	3/14 (21%)	4/14 (29%)	0	0
**Process**					
Clearly designated person or team leads the effort to continue further implementation of genetic testing into clinical practice	2/13 (15%)	4/13 (31%)	5/13 (39%)	2/13 (15%)	0
Variety of strategies are being used to enable staff to continue further implementation of genetic testing	1/13 (8%)	4/13 (31%)	5/13 (39%)	2/13 (15%)	1/13 (8%)
Implementation leaders have the necessary qualities and skills to successfully incorporate genetic testing into clinical practice	0	3/13 (23%)	7/13 (54%)	2/13 (15%)	1/13 (8%)

The order of barriers was adjusted for the figures to facilitate interpretation, ranked from the most to the least frequently reported barrier.

*=turned around agree/disagree options in the graphs to improve visual interpretation of barriers (red bars).

Supplementary Table S9. Barrier statements and responses from non-OECD countries.

Barriers to implementation of genetic testing in daily practice	Strongly disagree/disagree	Neutral	Agree/strongly agree	Don't know	NA
Costs of genetic testing are a barrier*	10/45 (22%)	1/45 (2%)	33/45 (73%)	0	1/45 (2%)
**Outer setting**					
Insurance for individuals with genetic diagnosis may prevent further implementation*	8/42 (19%)	6/42 (14%)	12/42 (29%)	12/42 (29%)	4/42 (10%)
Legislation and regulations allow further implementation	7/42 (17%)	8/42 (19%)	20/42 (48%)	6/42 (14%)	1/42 (2%)
There are clear professional group guidelines and recommendations for genetic testing in kidney disease in my country	19/42 (45%)	8/42 (19%)	8/42 (19%)	3/42 (7%)	4/42 (10%)
**Inner setting**					
I have enough time to facilitate genetic testing	11/42 (26%)	9/42 (21%)	19/42 (45%)	0	3/42 (7%)
I can find/use reliable sources of information to apply for genetic testing	7/42 (17%)	4/42 (10%)	31/42 (74%)	0	0
Sufficient clinical genetic staffing levels for patients with suspected hereditary kidney disease	16/42 (38%)	8/42 (19%)	14/42 (33%)	2/42 (5%)	2/42 (5%)
Clear protocol for eligible patients and workflow to obtain genetic testing	22/42 (52%)	4/42 (10%)	12/42 (29%)	0	4/42 (10%)
Sufficient multidisciplinary network to facilitate genetic testing in patients with suspected hereditary kidney disease	12/42 (29%)	7/42 (17%)	18/42 (43%)	0	5/42 (12%)
Clear allocation of responsibilities between health care practitioners	11/42 (26%)	8/42 (19%)	16/42 (38%)	1/42 (2%)	6/42 (14%)
Further implementation of genetic testing should be prioritized over other initiatives to improve care for CKD patients	4/42 (10%)	7/42 (17%)	29/42 (69%)	0	2/42 (5%)
Technical facilities are sufficient	15/42 (36%)	6/42 (14%)	15/42 (36%)	2/42 (5%)	4/42 (10%)
My clinic's financial resources are sufficient to facilitate further implementation of genetic testing	20/42 (48%)	7/42 (17%)	8/42 (19%)	2/42 (5%)	5/42 (12%)
**Individual characteristics**					
I am confident in my ability to use the results of genetic testing	3/41 (7%)	5/41 (12%)	33/41 (81%)	0	0
My training has prepared me to look after patients at high risk for hereditary kidney disease	3/41 (7%)	3/41 (7%)	35/41 (85%)	0	0
My religion and ethical believes are not a barrier for further implementation of genetic testing	1/41 (2%)	0	40/41 (98%)	0	0
Genetic testing improves my ability to care for my patients	0	2/41 (5%)	39/41 (95%)	0	0
I only request genetic diagnostics when there are clinical implications involved	10/41 (24%)	2/41 (5%)	28/41 (68%)	0	1/41 (2%)
There is robust evidence to support the effectiveness of genetic testing	1/40 (3%)	4/40 (10%)	34/40 (85%)	1/40 (3%)	0
Genetic testing is an improvement over non-genetic clinical investigations for suspected hereditary kidney disease	0	2/40 (5%)	38/40 (95%)	0	0
Turnaround time is a barrier for further implementation of genetic testing in the clinical workflow*	11/40 (28%)	8/40 (20%)	20/40 (50%)	1/40 (3%)	0
**Process**					
Clearly designated person or team leads the effort to continue further implementation of genetic testing into clinical practice	3/40 (8%)	12/40 (30%)	23/40 (58%)	1/40 (3%)	1/40 (3%)
Variety of strategies are being used to enable staff to continue further implementation of genetic testing	4/40 (10%)	9/40 (23%)	23/40 (58%)	2/40 (5%)	2/40 (5%)
Implementation leaders have the necessary qualities and skills to successfully incorporate genetic testing into clinical practice	3/40 (8%)	7/40 (18%)	27/40 (68%)	1/40 (3%)	2/40 (5%)

The order of barriers was adjusted for the figures to facilitate interpretation, ranked from the most to the least frequently reported barrier.

*=turned around agree/disagree options in the graphs to improve visual interpretation of barriers (red bars).

Supplementary Table S10. Added value of genetic testing for different suspected CKD diagnoses according to survey participants.

Individual characteristics, added value genetic testing to different diagnoses	Very unimportant/unimportant	Neutral	Important/very important	Don't know	NA
**Adult care**					
Glomerulopathies	3/106 (3%)	5/106 (5%)	97/106 (92%)	1/106 (1%)	0
CAKUT	4/106 (4%)	22/106 (21%)	76/106 (72%)	4/106 (4%)	0
ADPKD	3/106 (3%)	9/106 (9%)	94/106 (89%)	0	0
ARPKD	5/106 (5%)	7/106 (7%)	93/106 (88%)	1/106 (1%)	0
Nephronophthisis	1/106 (1%)	3/106 (3%)	98/106 (92%)	1/106 (1%)	3/106 (3%)
Other ciliopathies	1/106 (1%)	5/106 (5%)	99/106 (93%)	1/106 (1%)	0
Tubulopathies	1/106 (1%)	4/106 (4%)	101/106 (95%)	0	0
Thrombotic microangiopathies	2/106 (2%)	4/106 (4%)	100/106 (94%)	0	0
Metabolic nephropathies	5/106 (5%)	8/106 (8%)	92/106 (87%)	1/106 (1%)	0
Rare causes of hypertension	6/106 (6%)	21/106 (20%)	76/106 (72%)	3/106 (3%)	0
CKD of unknown etiology (CKDx)	0	6/106 (6%)	99/106 (93%)	1/106 (1%)	0
**Pediatric care**					
Glomerulopathies	1/135 (1%)	1/135 (1%)	132/135 (98%)	1/135 (1%)	0
CAKUT	9/135 (7%)	35/135 (26%)	90/135 (67%)	1/135 (1%)	0
ADPKD	5/135 (4%)	20/135 (15%)	108/135 (80%)	1/135 (1%)	1/135 (1%)
ARPKD	0	7/135 (5%)	127/135 (94%)	1/135 (1%)	0
Nephronophthisis	0	2/135 (2%)	132/135 (98%)	1/135 (1%)	0
Other ciliopathies	0	2/135 (2%)	132/135 (98%)	1/135 (1%)	0
Tubulopathies	1/135 (1%)	1/135 (1%)	132/135 (98%)	1/135 (1%)	0
Thrombotic microangiopathies	0	4/135 (3%)	130/135 (96%)	1/135 (1%)	0
Metabolic nephropathies	1/135 (1%)	1/135 (1%)	131/135 (97%)	2/135 (2%)	0
Rare causes of hypertension	0	17/135 (13%)	116/135 (86%)	2/135 (2%)	0
CKD of unknown etiology (CKDx)	0	6/135 (4%)	128/135 (95%)	1/135 (1%)	0
**Prenatal care**					
CAKUT	12/51 (24%)	17/51 (33%)	21/51 (41%)	1/51 (2%)	0
Ciliopathies	1/51 (2%)	7/51 (14%)	41/51 (80%)	1/51 (2%)	0

CAKUT = Congenital Anomalies of the Kidney and Urinary Tract, ADPKD = Autosomal Dominant Polycystic Kidney Disease, ARPKD = Autosomal Recessive Polycystic Kidney Disease, CKD = chronic kidney disease.

Supplementary Table S11. Logistic regression subgroup analyses: OECD-regions.

Predictor	Barrier experience (n barriers/total n variable)	OR [95% CI]
Univariable analyses		
**Profession**		
Pediatric nephrologist	38/94 (40%)	0.8 [0.5, 1.5]
Adult nephrologist	36/78 (46%)	1.3 [0.7, 2.3]
Clinical geneticist	4/15 (27%)	0.5 [0.1, 1.5]
**Type of care**		
**Pediatric**	**36/101 (36%)**	**0.5 [0.3, 0.9]**
Adult	46/97 (47%)	1.5 [0.8, 2.7]
Prenatal	21/50 (42%)	0.9 [0.5, 1.8]
**Location diagnostic testing**		
Own hospital	49/125 (39%)	0.6 [0.3, 1.2]
Other hospital own country	48/103 (47%)	1.4 [0.8, 2.5]
**Commercial lab own country**	**26/46 (57%)**	**2.1 [1.1, 4.1]**
Commercial lab other country	7/12 (58%)	1.9 [0.6, 6.4]
**Lab other hospital other country**	**12/16 (75%)**	**4.5 [1.4, 14.6]**
Only through research	5/6 (83%)	7.0 [0.8, 61.4]
**Region**		
West	34/87 (39%)	0.7 [0.4, 1.3]
**East**	**17/27 (63%)**	**2.6 [1.1, 6.1]**
South	23/56 (41%)	0.9 [0.5, 1.7]
Nordic	5/14 (36%)	0.7 [0.2, 2.2]
**Which party usually pays for genetic testing**		
**Patient/family**	**15/19 (79%)**	**5.9 [1.9, 18.6]**
Health insurance	27/60 (45%)	1.1 [0.6, 2.1]
Public health service	54/123 (44%)	1.1 [0.6, 2.1]
**Hospital / local budget**	**30/48 (63%)**	**3.0 [1.5, 5.8]**
Commercial company for free	4/8 (50%)	1.3 [0.3, 5.6]
**Research funded**	**23/34 (68%)**	**3.5 [1.6, 7.7]**
ERKnet member/ affiliated	52/127 (41%)	0.8 [0.4, 1.4]
EU-member, yes	76/173 (44%)	2.1 [0.5, 8.1]
Multivariable analyses		
**Type of care, pediatric**	**36/101 (36%)**	**0.4 [0.2, 0.9]**
Location diagnostic testing, commercial lab own country	26/46 (57%)	1.6 [0.8, 3.5]
Location diagnostic testing, lab other hospital other country	12/16 (75%)	2.5 [0.6, 9.9]
Region, East	17/27 (63%)	2.1 [0.8, 5.6]
**Which party usually pays for genetic testing, patient/family**	**15/19 (79%)**	**5.9 [1.7, 20.3]**
**Hospital / local budget**	**30/48 (63%)**	**2.2 [1.0, 4.8]**
**Research funded**	**23/34 (68%)**	**3.5 [1.5, 8.4]**

Supplementary Table S12. Barrier statements and responses from pediatric nephrologists.

**Barriers to implementation of genetic testing in daily practice**	**Strongly disagree/disagree**	**Neutral**	**Agree/strongly agree**	**Don't know**	**NA**
Costs of genetic testing are a barrier*	56/126 (44%)	13/126 (10%)	44/126 (35%)	2/126 (2%)	11/126 (9%)
**Outer setting**					
Insurance for individuals with genetic diagnosis may prevent further implementation*	27/121 (22%)	16/121 (13%)	40/121 (33%)	19/121 (16%)	19/121 (16%)
Legislation and regulations allow further implementation	11/120 (9%)	18/120 (15%)	72/120 (60%)	12/120 (10%)	7/120 (6%)
There are clear professional group guidelines and recommendations for genetic testing in kidney disease in my country	32/121 (26%)	24/121 (20%)	57/121 (47%)	5/121 (4%)	3/121 (3%)
**Inner setting**					
I have enough time to facilitate genetic testing	28/120 (23%)	17/120 (14%)	73/120 (61%)	0	2/120 (2%)
I can find/use reliable sources of information to apply for genetic testing	13/120 (11%)	10/120 (8%)	97/120 (81%)	0	0
Sufficient clinical genetic staffing levels for patients with suspected hereditary kidney disease	27/119 (23%)	11/119 (9%)	77/119 (65%)	2/119 (2%)	2/119 (2%)
Clear protocol for eligible patients and workflow to obtain genetic testing	40/119 (34%)	27/119 (23%)	49/119 (41%)	0	3/119 (3%)
Sufficient multidisciplinary network to facilitate genetic testing in patients with suspected hereditary kidney disease	14/119 (12%)	15/119 (13%)	87/119 (73%)	0	3/119 (3%)
Clear allocation of responsibilities between health care practitioners	14/119 (12%)	20/119 (17%)	78/119 (66%)	1/119 (1%)	6/119 (5%)
Further implementation of genetic testing should be prioritized over other initiatives to improve care for CKD patients	31/119 (26%)	31/119 (26%)	54/119 (45%)	0	3/119 (3%)
Technical facilities are sufficient	19/119 (16%)	19/119 (16%)	66/119 (56%)	9/119 (8%)	6/119 (5%)
My clinic's financial resources are sufficient to facilitate further implementation of genetic testing	33/119 (28%)	19/119 (16%)	43/119 (36%)	13/119 (11%)	11/119 (9%)
**Individual characteristics**					
I am confident in my ability to use the results of genetic testing	8/118 (7%)	10/118 (9%)	100/118 (85%)	0	0
My training has prepared me to look after patients at high risk for hereditary kidney disease	5/118 (4%)	4/118 (3%)	109/118 (92%)	0	0
My religion and ethical believes are not a barrier for further implementation of genetic testing	3/118 (3%)	1/118 (1%)	113/118 (96%)	0	1/118 (1%)
Genetic testing improves my ability to care for my patients	0	0	118/118 (100%)	0	0
I only request genetic diagnostics when there are clinical implications involved	31/118 (26%)	11/118 (9%)	75/118 (64%)	0	1/118 (1%)
There is robust evidence to support the effectiveness of genetic testing	4/118 (3%)	13/118 (11%)	100/118 (85%)	1/118 (1%)	0
Genetic testing is an improvement over non-genetic clinical investigations for suspected hereditary kidney disease	2/118 (2%)	3/118 (3%)	112/118 (95%)	0	1/118 (1%)
Turnaround time is a barrier for further implementation of genetic testing in the clinical workflow*, **	29/118 (25%)	18/118 (15%)	69/118 (59%)	2/118 (2%)	0
**Process**					
Clearly designated person or team leads the effort to continue further implementation of genetic testing into clinical practice	13/116 (11%)	20/116 (17%)	78/116 (67%)	4/116 (3%)	1/116 (1%)
Variety of strategies are being used to enable staff to continue further implementation of genetic testing	9/116 (8%)	19/116 (16%)	77/116 (66%)	8/116 (7%)	3/116 (3%)
Implementation leaders have the necessary qualities and skills to successfully incorporate genetic testing into clinical practice	3/116 (3%)	12/116 (10%)	92/116 (79%)	2/116 (2%)	7/116 (6%)

The order of barriers was adjusted for the figures to facilitate interpretation, ranked from the most to the least frequently reported barrier.

Supplementary Table S13. Barrier statements and responses from adult nephrologists.

**Barriers to implementation of genetic testing in daily practice**	**Strongly disagree/disagree**	**Neutral**	**Agree/strongly agree**	**Don't know**	**NA**
Costs of genetic testing are a barrier*	47/91 (52%)	7/91 (8%)	32/91 (35%)	1/91 (1%)	4/91 (4%)
**Outer setting**					
Insurance for individuals with genetic diagnosis may prevent further implementation*	26/87 (30%)	13/87 (15%)	19/87 (22%)	13/87 (15%)	16/87 (18%)
Legislation and regulations allow further implementation	10/87 (12%)	12/87 (14%)	50/87 (58%)	13/87 (15%)	2/87 (2%)
There are clear professional group guidelines and recommendations for genetic testing in kidney disease in my country	40/87 (46%)	11/87 (13%)	32/87 (37%)	3/87 (3%)	1/87 (1%)
**Inner setting**					
I have enough time to facilitate genetic testing	36/87 (41%)	11/87 (13%)	38/87 (44%)	0	2/87 (2%)
I can find/use reliable sources of information to apply for genetic testing	17/87 (20%)	12/87 (14%)	58/87 (67%)	0	0
Sufficient clinical genetic staffing levels for patients with suspected hereditary kidney disease	33/87 (38%)	10/87 (12%)	43/87 (49%)	1/87 (1%)	0
Clear protocol for eligible patients and workflow to obtain genetic testing	40/87 (46%)	14/87 (16%)	30/87 (35%)	1/87 (1%)	2/87 (2%)
Sufficient multidisciplinary network to facilitate genetic testing in patients with suspected hereditary kidney disease	21/87 (24%)	14/87 (16%)	49/87 (56%)	0	3/87 (3%)
Clear allocation of responsibilities between health care practitioners	23/87 (26%)	19/87 (22%)	42/87 (48%)	1/87 (1%)	2/87 (2%)
Further implementation of genetic testing should be prioritized over other initiatives to improve care for CKD patients	11/87 (13%)	28/87 (32%)	48/87 (55%)	0	0
Technical facilities are sufficient	22/87 (25%)	9/87 (10%)	56/87 (64%)	0	0
My clinic's financial resources are sufficient to facilitate further implementation of genetic testing	29/87 (33%)	18/87 (21%)	34/87 (39%)	3/87 (3%)	3/87 (3%)
**Individual characteristics**					
I am confident in my ability to use the results of genetic testing	6/86 (7%)	9/86 (11%)	71/86 (83%)	0	0
My training has prepared me to look after patients at high risk for hereditary kidney disease	10/86 (12%)	12/86 (14%)	64/86 (74%)	0	0
My religion and ethical believes are not a barrier for further implementation of genetic testing	1/86 (1%)	0	84/86 (98%)	0	1/86 (1%)
Genetic testing improves my ability to care for my patients	0	2/86 (2%)	84/86 (98%)	0	0
I only request genetic diagnostics when there are clinical implications involved	29/86 (34%)	11/86 (13%)	45/86 (52%)	0	1/86 (1%)
There is robust evidence to support the effectiveness of genetic testing	0	13/86 (15%)	69/86 (80%)	4/86 (5%)	0
Genetic testing is an improvement over non-genetic clinical investigations for suspected hereditary kidney disease	0	2/86 (2%)	83/86 (97%)	1/86 (1%)	0
Turnaround time is a barrier for further implementation of genetic testing in the clinical workflow*, **	21/86 (24%)	13/86 (15%)	50/86 (58%)	2/86 (2%)	0
**Process**					
Clearly designated person or team leads the effort to continue further implementation of genetic testing into clinical practice	8/86 (9%)	8/86 (9%)	66/86 (77%)	3/86 (4%)	1/86 (1%)
Variety of strategies are being used to enable staff to continue further implementation of genetic testing	11/86 (13%)	16/86 (19%)	52/86 (61%)	6/86 (7%)	1/86 (1%)
Implementation leaders have the necessary qualities and skills to successfully incorporate genetic testing into clinical practice	8/86 (9%)	15/86 (17%)	60/86 (70%)	2/86 (2%)	1/86 (1%)

The order of barriers was adjusted for the figures to facilitate interpretation, ranked from the most to the least frequently reported barrier.

Supplementary Table S14. Barrier statements and responses from clinical geneticists.

**Barriers to implementation of genetic testing in daily practice**	**Strongly disagree/disagree**	**Neutral**	**Agree/strongly agree**	**Don't know**	**NA**
Costs of genetic testing are a barrier*	12/18 (67%)	0	3/18 (17%)	0	3/18 (17%)
**Outer setting**					
Insurance for individuals with genetic diagnosis may prevent further implementation*	9/16 (56%)	1/16 (6%)	2/16 (13%)	1/16 (6%)	3/16 (19%)
Legislation and regulations allow further implementation	2/16 (13%)	2/16 (13%)	12/16 (75%)	0	0
There are clear professional group guidelines and recommendations for genetic testing in kidney disease in my country	6/16 (38%)	2/16 (13%)	7/16 (44%)	0	1/16 (6%)
**Inner setting**					
I have enough time to facilitate genetic testing	1/16 (6%)	4/16 (25%)	10/16 (63%)	0	1/16 (6%)
I can find/use reliable sources of information to apply for genetic testing	0	1/16 (6%)	14/16 (88%)	0	1/16 (6%)
Sufficient clinical genetic staffing levels for patients with suspected hereditary kidney disease	4/16 (25%)	2/16 (13%)	10/16 (63%)	0	0
Clear protocol for eligible patients and workflow to obtain genetic testing	4/16 (25%)	3/16 (19%)	9/16 (56%)	0	0
Sufficient multidisciplinary network to facilitate genetic testing in patients with suspected hereditary kidney disease	1/16 (6%)	1/16 (6%)	14/16 (88%)	0	0
Clear allocation of responsibilities between health care practitioners	2/16 (13%)	1/16 (6%)	13/16 (81%)	0	0
Further implementation of genetic testing should be prioritized over other initiatives to improve care for CKD patients	4/16 (25%)	4/16 (25%)	8/16 (50%)	0	0
Technical facilities are sufficient	3/16 (19%)	0	13/16 (81%)	0	0
My clinic's financial resources are sufficient to facilitate further implementation of genetic testing	3/16 (19%)	3/16 (19%)	8/16 (50%)	0	2/16 (13%)
**Individual characteristics**					
I am confident in my ability to use the results of genetic testing	0	0	16/16 (100%)	0	0
My training has prepared me to look after patients at high risk for hereditary kidney disease	0	1/16 (6%)	15/16 (94%)	0	0
My religion and ethical believes are not a barrier for further implementation of genetic testing	1/16 (6%)	0	15/16 (94%)	0	0
Genetic testing improves my ability to care for my patients	0	1/16 (6%)	14/16 (88%)	0	1/16 (6%)
I only request genetic diagnostics when there are clinical implications involved	8/16 (50%)	1/16 (6%)	7/16 (44%)	0	0
There is robust evidence to support the effectiveness of genetic testing	0	1/15 (7%)	14/15 (93%)	0	0
Genetic testing is an improvement over non-genetic clinical investigations for suspected hereditary kidney disease	0	1/15 (7%)	14/15 (93%)	0	0
Turnaround time is a barrier for further implementation of genetic testing in the clinical workflow*, **	8/15 (53%)	3/15 (20%)	4/15 (27%)	0	0
**Process**					
Clearly designated person or team leads the effort to continue further implementation of genetic testing into clinical practice	1/15 (7%)	2/15 (13%)	12/15 (80%)	0	0
Variety of strategies are being used to enable staff to continue further implementation of genetic testing	3/15 (20%)	2/15 (13%)	9/15 (60%)	0	1/15 (7%)
Implementation leaders have the necessary qualities and skills to successfully incorporate genetic testing into clinical practice	1/15 (7%)	0	13/15 (87%)		1/15 (7%)

The order of barriers was adjusted for the figures to facilitate interpretation, ranked from the most to the least frequently reported barrier.

Supplementary Table S15. European Rare Kidney disease Registry (ERKReg) data, details on included genetically tested individuals.

Population-adjusted prevalence of patients with an indication for genetic testing per country (prevalence per 1,000,000 inhabitants) was calculated by dividing the number of patients with a genetic testing indication by the total population of each country. Assuming similar disease prevalence across Europe and an optimally functioning reference network, both the population-adjusted numbers and the proportion of indicated patients tested should be comparable between countries.

Country	Country size*	Patients with indication for genetic testing	Of whom truly tested	Population-adjusted prevalence patients with an indication for genetic testing **	Truly tested with indication (percentage)	Indication genetic testing rare ciliopathy°	Of whom truly tested°	Population-adjusted prevalence rare ciliopathy patients with an indication for genetic testing	Percentage truly tested rare ciliopathy patients
Austria	9158750	107	82	12	77	18	15	2	83
Belgium	11817096	1180	706	100	60	45	31	4	69
Croatia	3861967	14	13	4	93	2	2	1	100
Czech Republic	11817096	24	21	2	88	1	1	0	100
Denmark	5961249	21	20	4	95	4	4	1	100
Estonia	1374687	29	15	21	52	4	4	3	100
France	68467362	2985	1763	44	59	212	173	3	82
Germany	83456045	1229	847	15	69	179	131	2	73
Greece	10400720	22	9	2	41				
Hungary	9584627	23	19	2	83	6	6	1	100
Republic of Ireland	5351681	869	543	162	62	32	27	6	84
Italy	58971230	3233	1720	55	53	238	156	4	66
Latvia	1871882	13	6	7	46	1			
Lithuania	2885891	350	265	121	76	16	8	6	50
Netherlands	17942942	675	484	38	72	71	62	4	87
Norway	5550217	17	9	3	53	1	1	0	100
Poland	36620970	188	54	5	29	21	12	1	57
Portugal	10639726	74	39	7	53	6	5	1	83
Romania	19067576	163	95	9	58	1	1	0	100

Russia	146000000	12	5	0	42				
Serbia	6605168	40	18	6	45	4	4	1	100
Slovenia	2123949	164	81	77	49	12	9	6	75
Spain	48619695	760	454	16	60	51	39	1	76
Turkey	85372377	114	80	1	70	1		0	0
United Kingdom	69138192	120	82	2	68	12	4	0	33

*Eurostat. **Prevalence per 1000 000 inhabitants. ° Excluded ADPKD patients

Supplementary Table S16. European Rare Kidney disease Registry (ERKReg) data, details on included genetically tested individuals, per European Region.

**Regions**	**Total inhabitants region**	**Indication testing**	**Truly tested**	**Truly tested with indication (percentage)**	**Population-adjusted prevalence patients with an indication for genetic testing***
**West**	265332068	7165	4507	63	27
**North**	11511466	38	29	76	3
**East**	65362561	791	461	58	12
**South**	128631371	4089	2222	54	32
**Non-OECD**	260907088	343	211	62	1

*Prevalence per 1000 000 inhabitants.

Checklist for Reporting Of Survey Studies (CROSS)

Section/topic	Item	Item description	Reported on page #
**Title and abstract**			
Title and abstract	1a	State the word "survey" along with a commonly used term in title or abstract to introduce the study's design.	2
	1b	Provide an informative summary in the abstract, covering background, objectives, methods, findings/results, interpretation/discussion, and conclusions.	2,3
**Introduction**			
Background	2	Provide a background about the rationale of study, what has been previously done, and why this survey is needed.	4
Purpose/aim	3	Identify specific purposes, aims, goals, or objectives of the study.	5
**Methods**			
Study design	4	Specify the study design in the methods section with a commonly used term (e.g., cross-sectional or longitudinal).	5
Data collection methods	5a	Describe the questionnaire (e.g., number of sections, number of questions, number and names of instruments used).	5,6
	5b	Describe all questionnaire instruments that were used in the survey to measure particular concepts. Report target population, reported validity and reliability information, scoring/classification procedure, and reference links (if any).	6,7
	5c	Provide information on pretesting of the questionnaire, if performed (in the article or in an online supplement). Report the method of pretesting, number of times questionnaire was pre-tested, number and demographics of participants used for pretesting, and the level of similarity of demographics between pre-testing participants and sample population.	6
	5d	Questionnaire if possible, should be fully provided (in the article, or as appendices or as an online supplement).	5
Sample characteristics	6a	Describe the study population (i.e., background, locations, eligibility criteria for participant inclusion in survey, exclusion criteria).	7

	6b	Describe the sampling techniques used (e.g., single stage or multistage sampling, simple random sampling, stratified sampling, cluster sampling, convenience sampling). Specify the locations of sample participants whenever clustered sampling was applied.	7
	6c	Provide information on sample size, along with details of sample size calculation.	8,10
	6d	Describe how representative the sample is of the study population (or target population if possible), particularly for population-based surveys.	18,19
Survey administration	7a	Provide information on modes of questionnaire administration, including the type and number of contacts, the location where the survey was conducted (e.g., outpatient room or by use of online tools, such as SurveyMonkey).	6,7
	7b	Provide information of survey's time frame, such as periods of recruitment, exposure, and follow-up days.	7
	7c	Provide information on the entry process:<br>–>For non-web-based surveys, provide approaches to minimize human error in data entry.<br>–>For web-based surveys, provide approaches to prevent "multiple participation" of participants.	7
Study preparation	8	Describe any preparation process before conducting the survey (e.g., interviewers' training process, advertising the survey).	7
Ethical considerations	9a	Provide information on ethical approval for the survey if obtained, including informed consent, institutional review board [IRB] approval, Helsinki declaration, and good clinical practice [GCP] declaration (as appropriate).	5
	9b	Provide information about survey anonymity and confidentiality and describe what mechanisms were used to protect unauthorized access.	5
Statistical analysis	10a	Describe statistical methods and analytical approach. Report the statistical software that was used for data analysis.	8,9
	10b	Report any modification of variables used in the analysis, along with reference (if available).	8
	10c	Report details about how missing data was handled. Include rate of missing items, missing data mechanism (i.e., missing completely at random [MCAR], missing at random [MAR] or missing not at random [MNAR]) and methods used to deal with missing data (e.g., multiple imputation).	8

	10d	State how non-response error was addressed.	NA
	10e	For longitudinal surveys, state how loss to follow-up was addressed.	NA
	10f	Indicate whether any methods such as weighting of items or propensity scores have been used to adjust for non-representativeness of the sample.	NA
	10g	Describe any sensitivity analysis conducted.	NA
**Results**			
Respondent characteristics	11a	Report numbers of individuals at each stage of the study. Consider using a flow diagram, if possible.	10
	11b	Provide reasons for non-participation at each stage, if possible.	10
	11c	Report response rate, present the definition of response rate or the formula used to calculate response rate.	10
	11d	Provide information to define how unique visitors are determined. Report number of unique visitors along with relevant proportions (e.g., view proportion, participation proportion, completion proportion).	7
Descriptive results	12	Provide characteristics of study participants, as well as information on potential confounders and assessed outcomes.	10, 29, 30
Main findings	13a	Give unadjusted estimates and, if applicable, confounder-adjusted estimates along with 95% confidence intervals and p-values.	14, 15
	13b	For multivariable analysis, provide information on the model building process, model fit statistics, and model assumptions (as appropriate).	8, 14, 15
	13c	Provide details about any sensitivity analysis performed. If there are considerable amount of missing data, report sensitivity analyses comparing the results of complete cases with that of the imputed dataset (if possible).	NA
**Discussion**			
Limitations	14	Discuss the limitations of the study, considering sources of potential biases and imprecisions, such as non-representativeness of sample, study design, important	19, 20

		uncontrolled confounders.	
Interpretations	15	Give a cautious overall interpretation of results, based on potential biases and imprecisions and suggest areas for future research.	20, Table1
Generalizability	16	Discuss the external validity of the results.	19, 20
**Other sections**			
Role of funding source	17	State whether any funding organization has had any roles in the survey's design, implementation, and analysis.	22
Conflict of interest	18	Declare any potential conflict of interest.	21
Acknowledgements	19	Provide names of organizations/persons that are acknowledged along with their contribution to the research.	21, 22
